# Supplementary material for: Synthesis of 4,4′-(4-Formyl-1H-pyrazole-1,3-diyl)dibenzoic Acid Derivatives as Narrow Spectrum Antibiotics for the Potential Treatment of Acinetobacter Baumannii Infections
Source: Antibiotics (Basel). 2020 Sep 28;9(10):650. doi: 10.3390/antibiotics9100650 (PMC7601628; doi:10.3390/antibiotics9100650)
Supplement: Supplementary file 1 [file antibiotics-09-00650-s001.pdf]

Synthesis of 4,4'-(4-Formyl-1*H*-pyrazole-1,3-diyl)dibenzoic Acid Derivatives as Narrow Spectrum Antibiotics for Potential Treatment of *Acinetobacter baumannii* Infections

Delancey,[a] E; Allison,[a] D.; Raj [a], H. KC.; Gilmore,[b] D. F.; Fite, [c,d] T.; Basnakian, [c,d] A.G.; Alam*[a], M. A.

✉ Mohammad A. Alam

malam@astate.edu

[a] Department of Chemistry and Physics, College of Science and Mathematics, Arkansas State University, Jonesboro, AR 72467
[b] Department of Biological Sciences, College of Science and Mathematics, Arkansas State University, Jonesboro, AR 72467
[c] Department of Pharmacology and Toxicology, University of Arkansas for Medical Sciences, 4301 W. Markham St., Little Rock, AR 72205
[d] Central Arkansas Veterans Healthcare System, W. 7th St., Little Rock, AR 72205

4,4'-(4-formyl-1*H*-pyrazole-1,3-diyl)dibenzoic acid (4)

Yellow solid (3.02g 90%). ^{1}H NMR, 300 MHz (DMSO-d$_6$): δ 9.99 (s, 1H), 9.41 (s, 1H), 8.09-8.05 (m, 8H); ^{13}C NMR (75MHz, DMSO-d$_6$) δ = 189.6, 172.1, 171.6, 156.9, 146.5, 141.1, 140.2, 136.1, 134.6, 133.9, 127.9, 124.0.

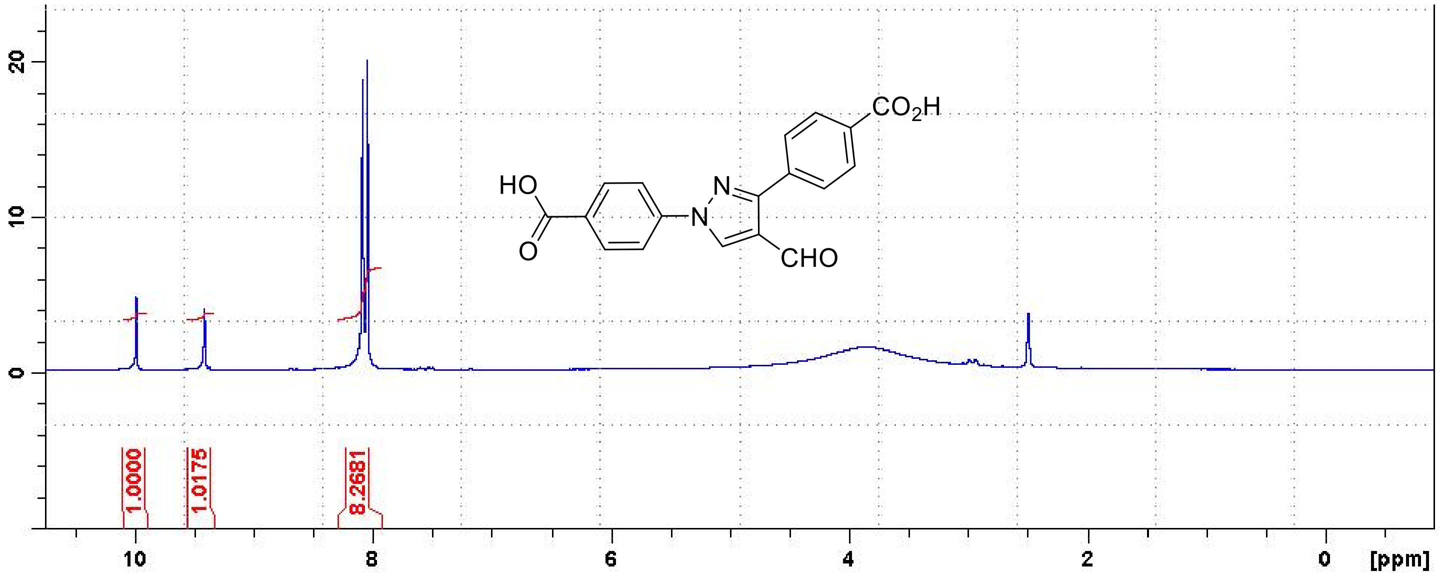

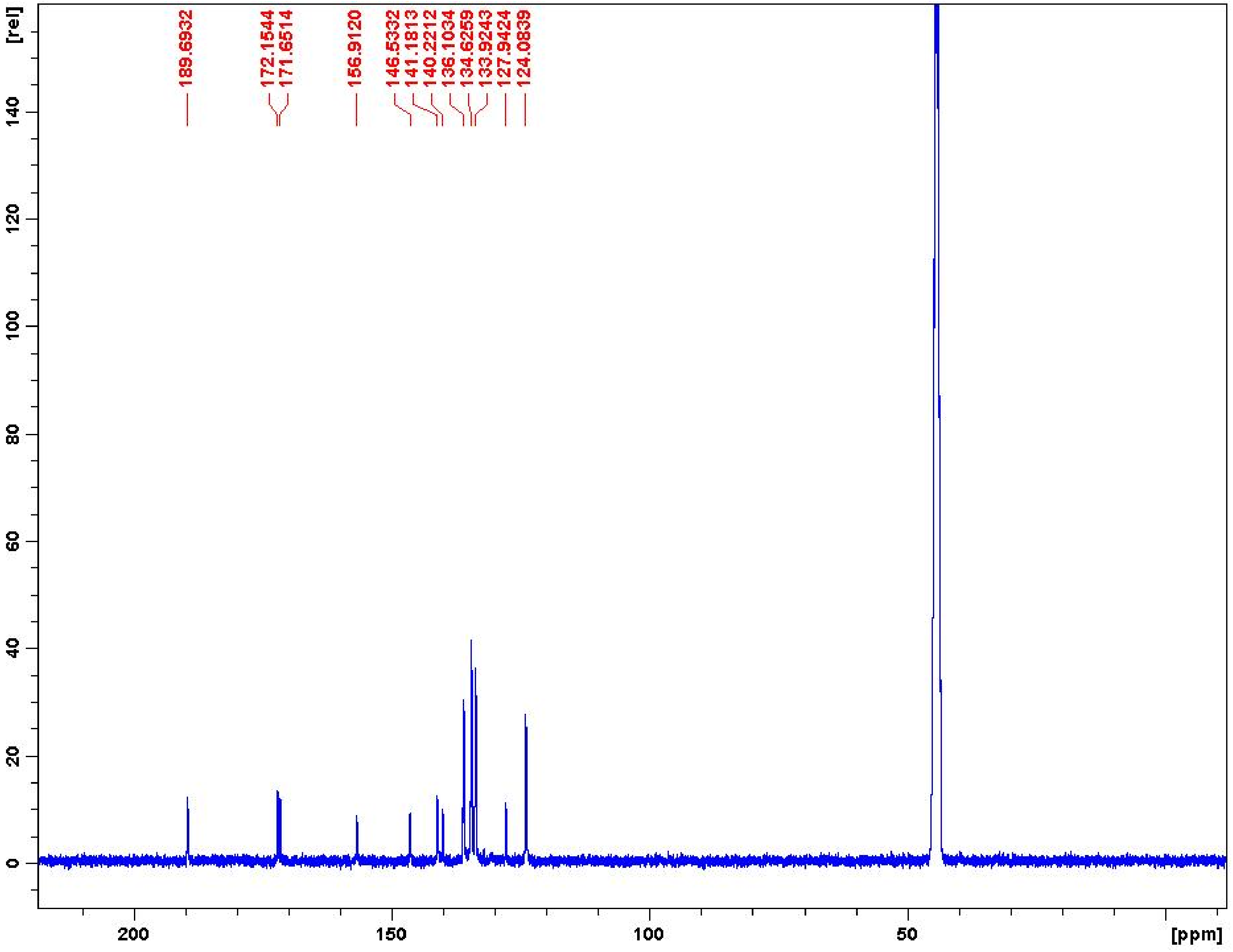

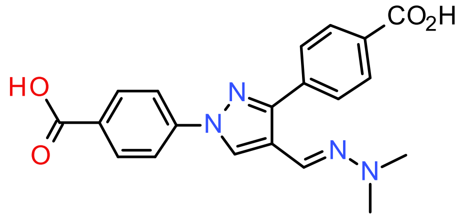

4-[1-(4-carboxyphenyl)-4-[(E)-(dimethylhydrazono)methyl]pyrazol-3-yl]benzoic acid (5)

Yellow solid (347 mg, 92%). ^{1}H NMR (300 MHz DMSO-d_6): 8.77 (s, 1H), 8.11-8.04 (m, 6H), 7.90 (d, J = 8.2 Hz, 2H), 7.29 (s, 1H), 2.88 (s, 6H); ^{13}C NMR (75 MHz DMSO-d_6): 167.5, 167.1, 149.9, 142.5, 137.2, 131.3, 130.7, 130.1, 128.8, 128.5, 126.4, 124.2, 120.9, 118.4, 42.8.

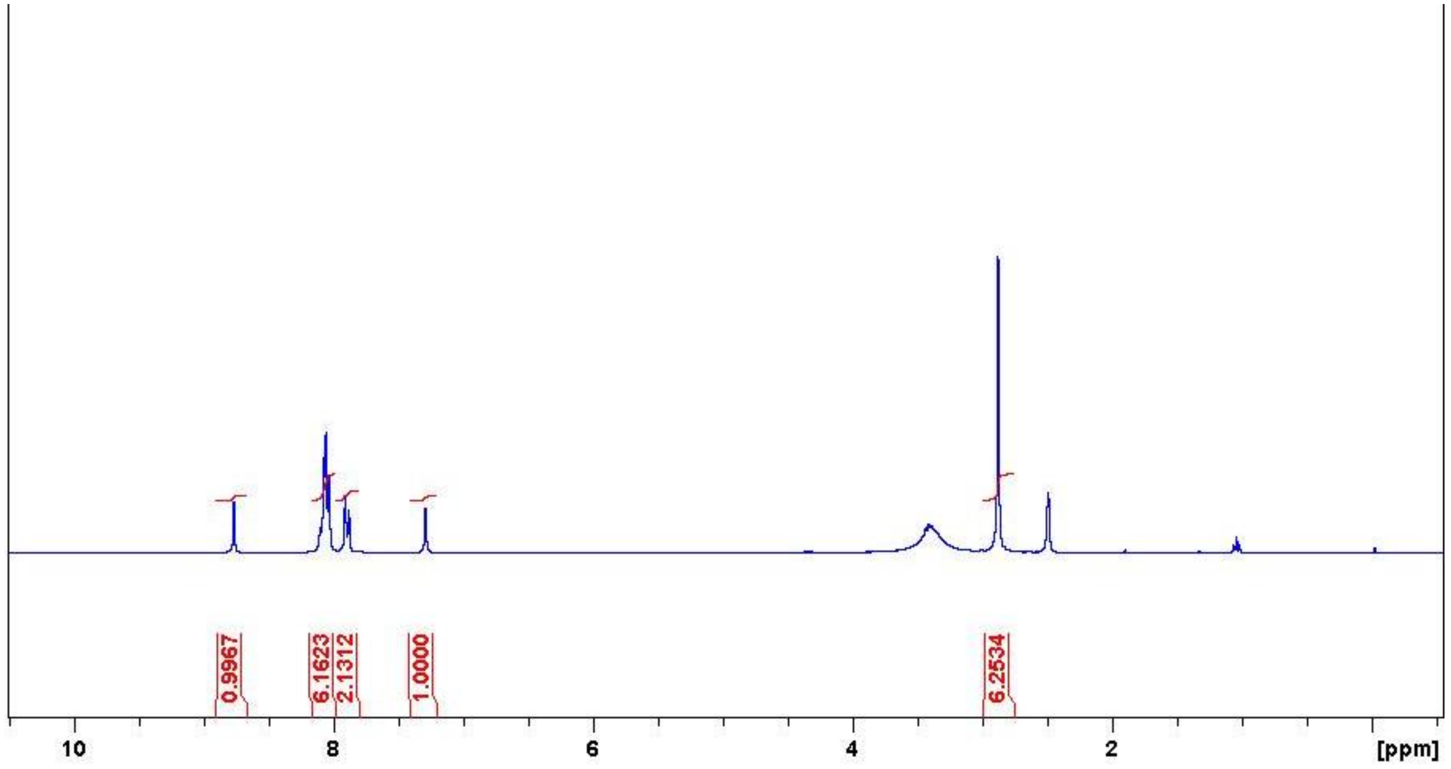
0.9967
6.1623
2.1312
1.0000
6.2534
10
8
6
4
2
[ppm]

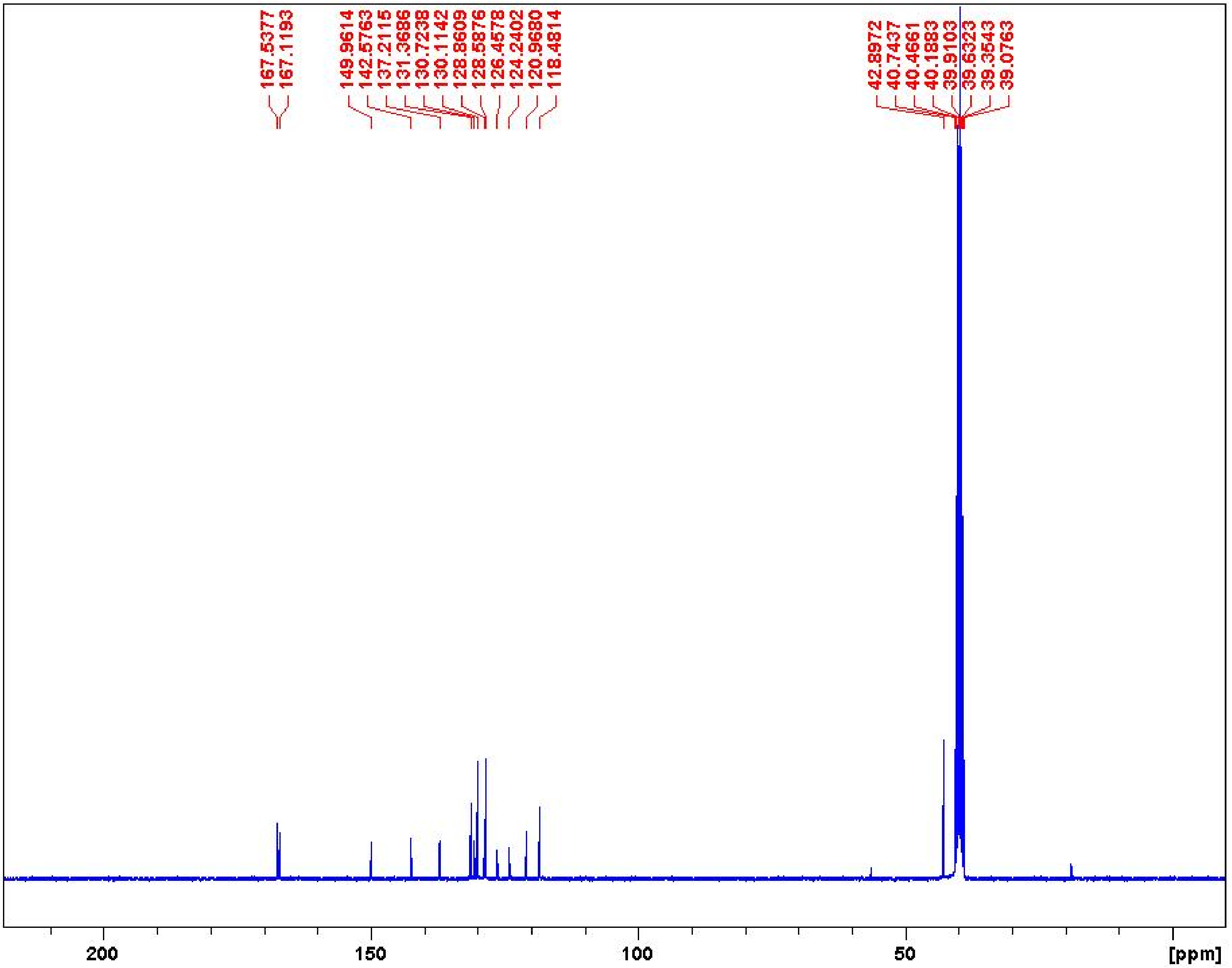

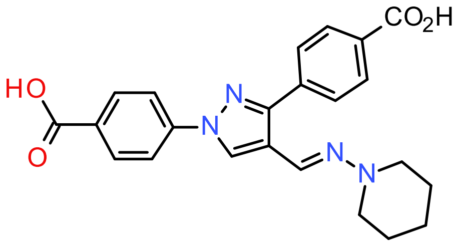

4-[1-(4-carboxyphenyl)-4-[(E)-1-piperidyliminomethyl]pyrazol-3-yl]benzoic acid (6)

Yellow solid, (376 mg, 91%). ^{1}H NMR 300 MHz (DMSO-d_6): 8.77 (s, 1H), 8.06-8.03 (m, 6H), 7.88 (d, J = 8.0 Hz, 2H), 7.59 (s, 1H), 3.05 (s, 4H), 1.63 (s, 4H), 1.46 (s, 2H); ^{13}C NMR (75MHz, DMSO-d_6) δ = 167.6, 167.2, 150.2, 142.4, 137.0, 131.3, 131.0, 130.0, 129.2, 128.6, 126.8, 126.1, 120.7, 118.4, 51.8, 25.0, 24.0.

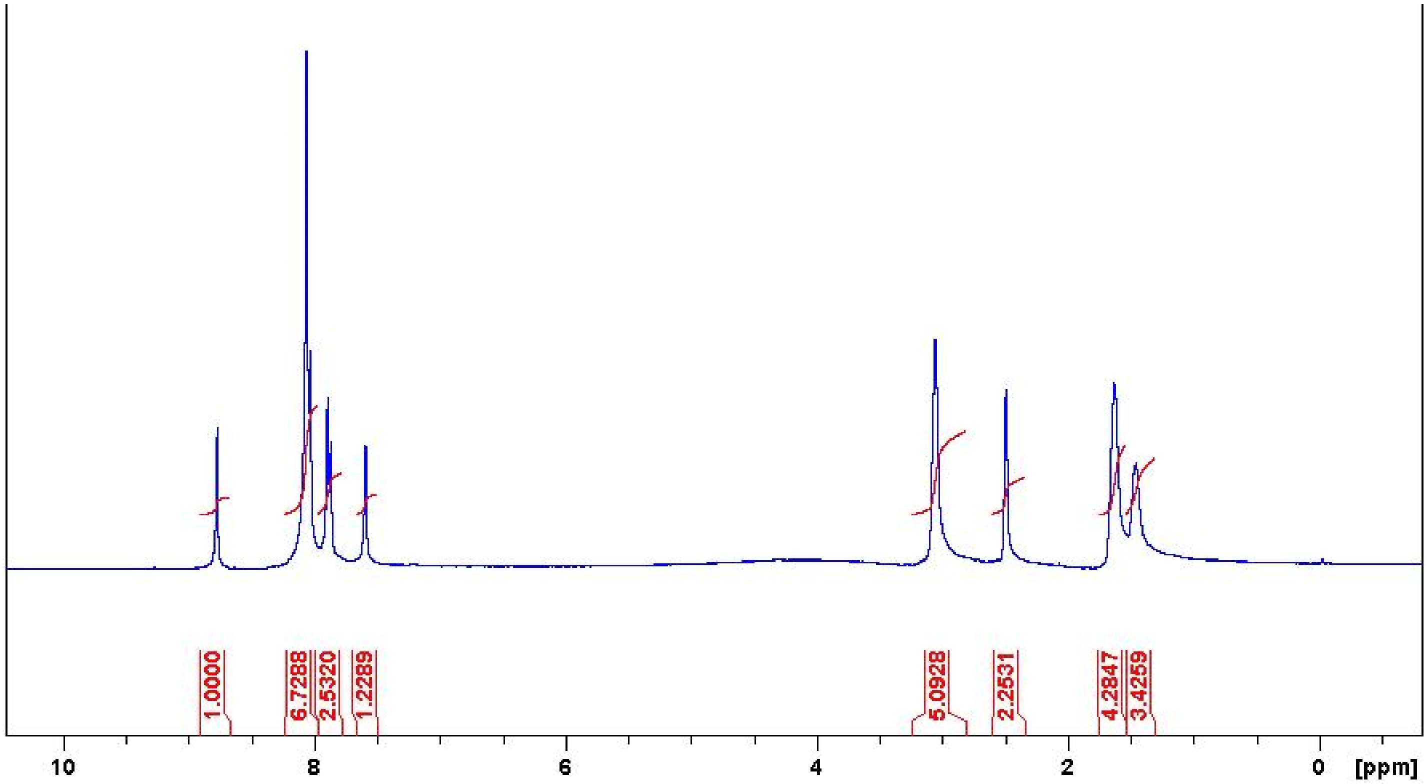
1.0000
6.7288
2.5320
1.2289
5.0928
2.2531
4.2847
3.4259
10
8
6
4
2
0
[ppm]

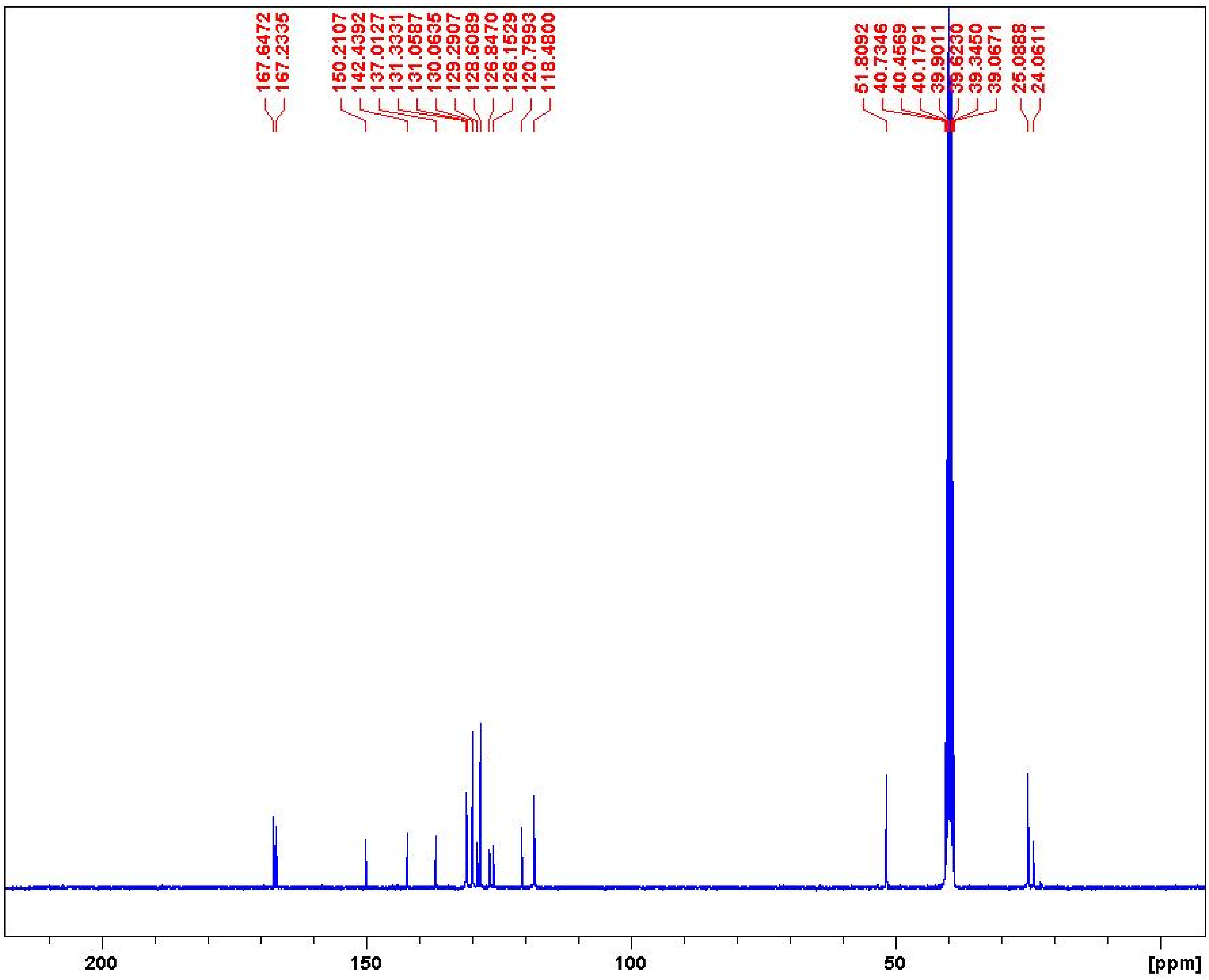

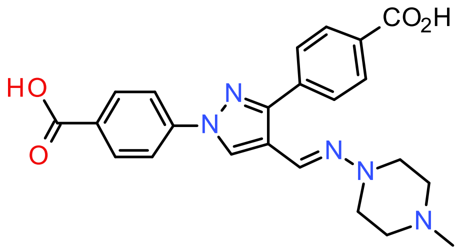

4,4'-{4-[(*E*)-(4-methylpiperazin-1-yl)imino)methyl]-1*H*-pyrazole-1,3-diyl}dibenzoic acid (7)

Yellow solid (402 mg, 93%). ^{1}H NMR 300 MHz (DMSO-d$_6$): δ 8.80 (s, 1H), 8.06 (s, 4H), 8.04 (d, J = 8.7 Hz, 2H), 7.88 (d, J = 8.3 Hz, 2H), 7.64 (s, 1H), 3.13 (br s, 4H), 2.60 (br s, 4H), 2.29 (s, 3H); ^{13}C NMR (75MHz, DMSO-d$_6$ + CDCl$_3$) δ = 167.8, 167.4, 150.3, 142.3, 136.7, 131.4, 131.3, 130.0, 129.7, 128.6, 128.0, 127.1, 120.3, 118.5, 53.9, 50.4, 45.4.

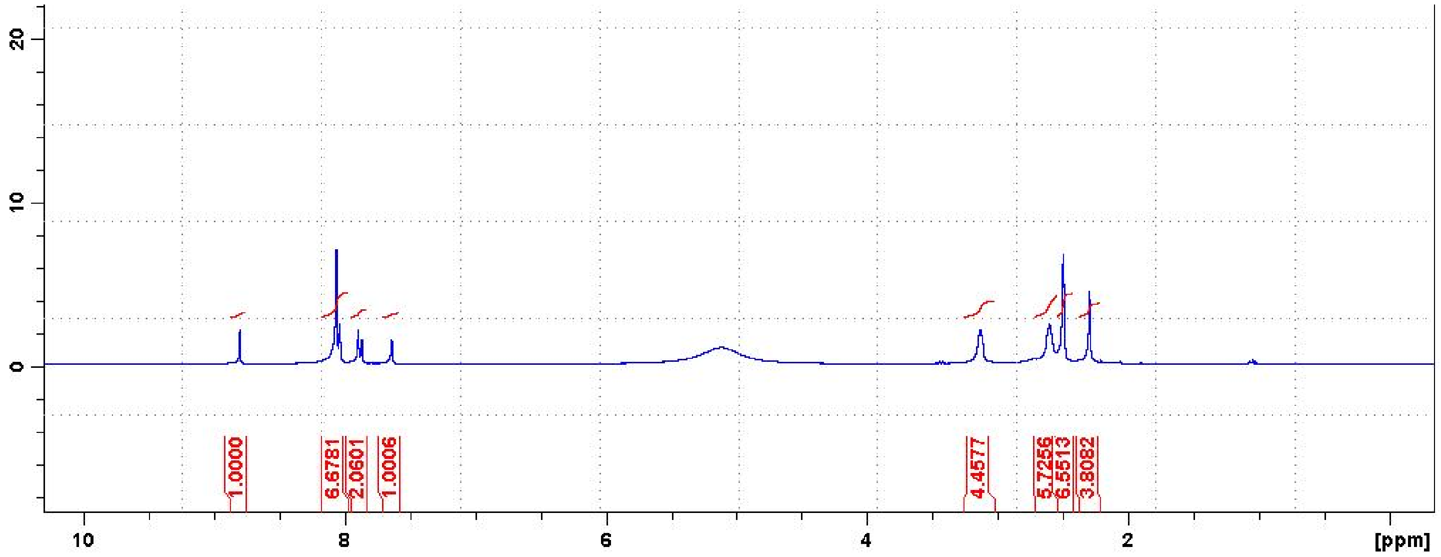

1.0000
6.6781
2.0601
1.0006
4.4577
5.7256
6.5513
3.8082
[ppm]

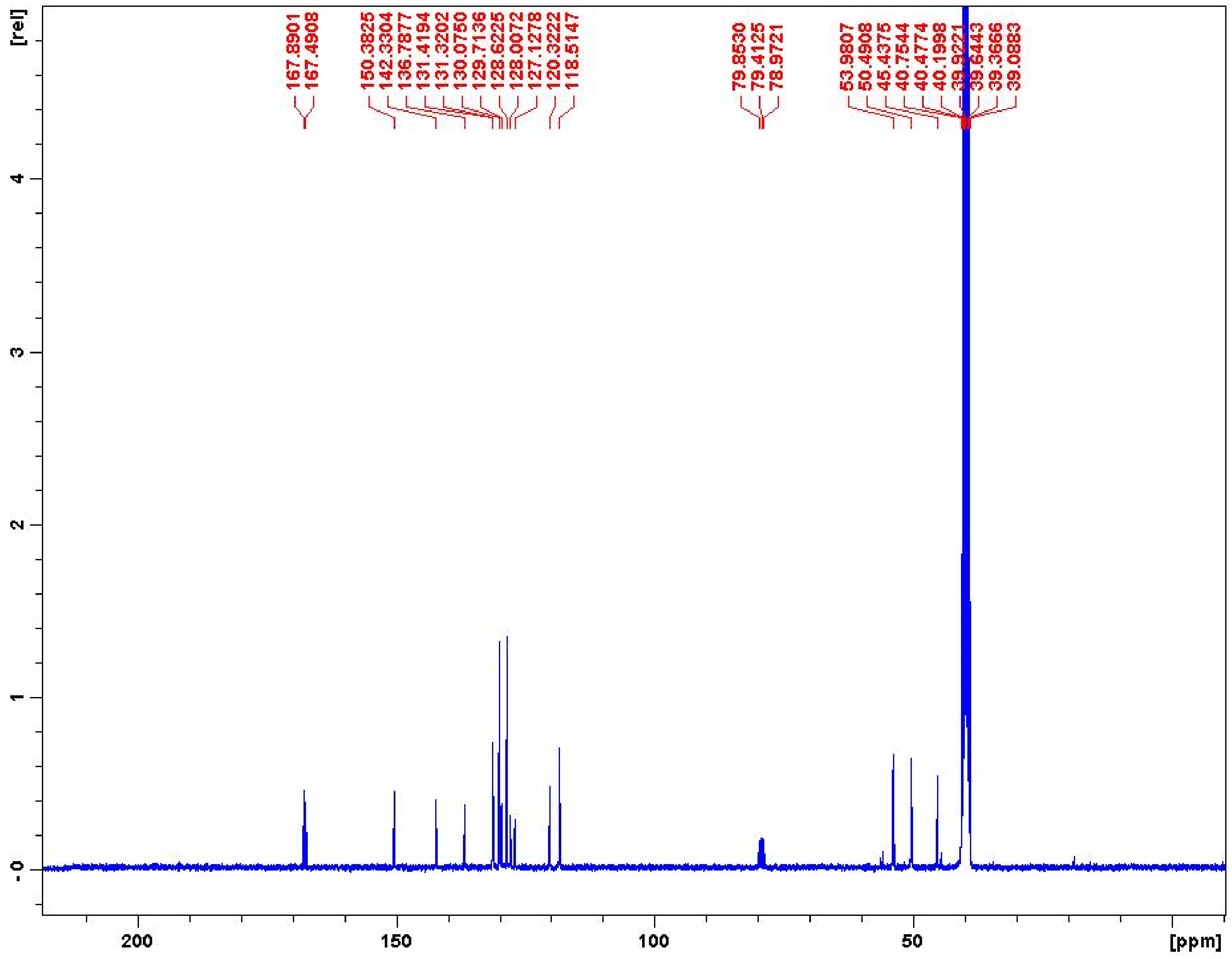

[rel]
167.8901
167.4908
150.3825
142.3304
136.7877
131.4194
131.3202
130.0750
129.7136
128.6225
128.0072
127.1278
120.3222
118.5147
79.8530
79.4125
78.9721
53.9807
50.4908
45.4375
40.7544
40.4774
40.1998
39.9221
39.6443
39.3666
39.0883
[ppm]

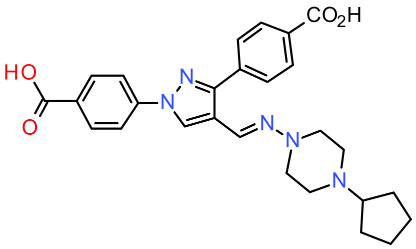

4-[1-(4-carboxyphenyl)-4-[(E)-(4-cyclopentylpiperazin-1-yl)iminomethyl]pyrazol-3-yl]benzoic acid (8)

Yellow solid (457 mg, 94%). ^{1}H NMR, 300 MHz (DMSO-d_6): 8.83 (s, 1H), 8.11-8.04 (m, 6H), 7.89 (d, J = 8. 1 Hz, 2H), 7.62 (s, 1H), 3.08 (s, 4H), 2.60 (s, 4H), 2.50 (s, 1H), 1.79 (s, 2H), 1.59-1.34 (m, 6H), ^{13}C NMR (75MHz, DMSO-d6) δ = 167.6, 167.1, 150.3, 142.4, 136.9, 131.3, 131.0, 130.1, 129.3, 128.6, 127.5, 127.0, 120.4, 118.5, 66.8, 51.2, 51.0, 30.3, 24.1.

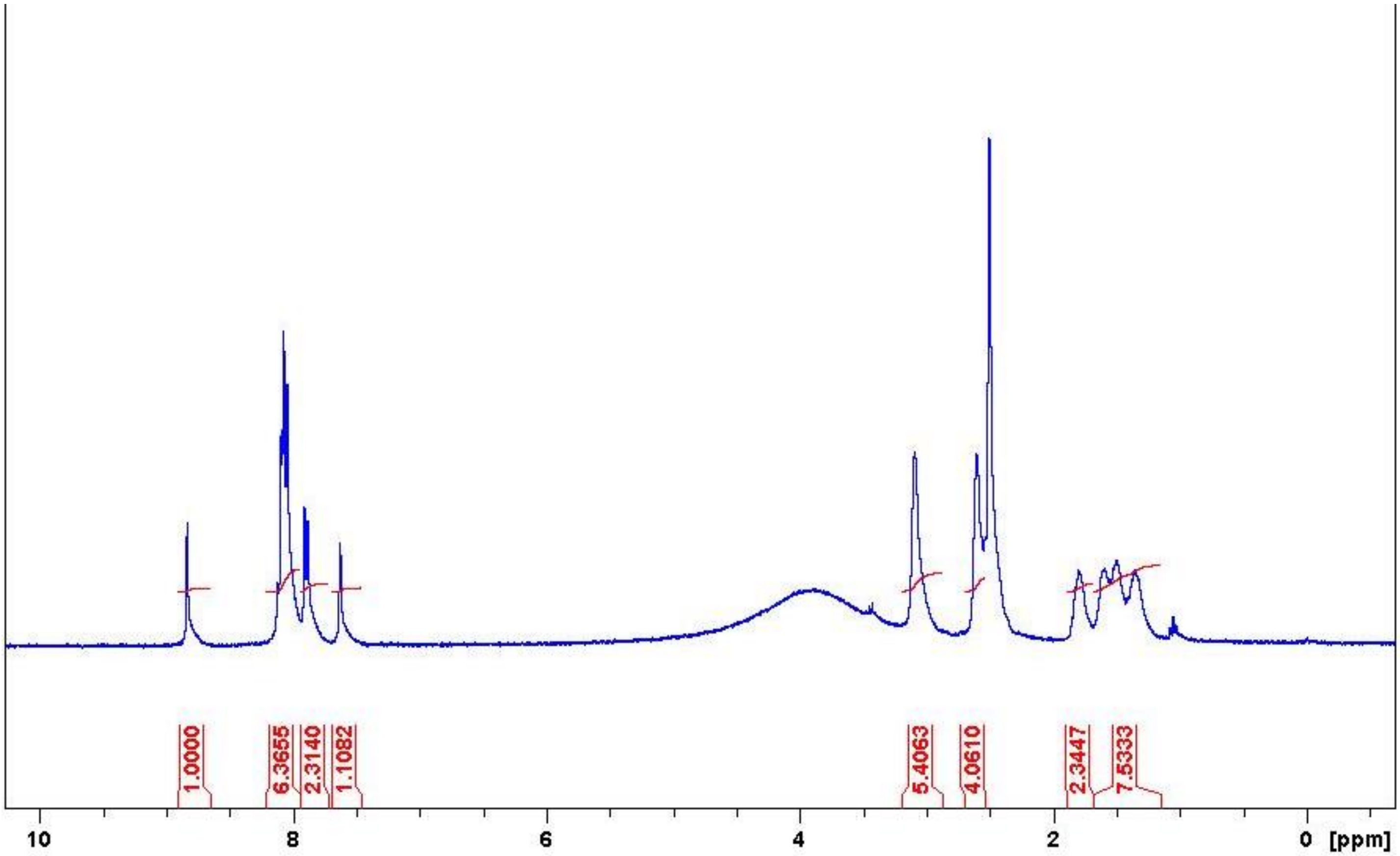

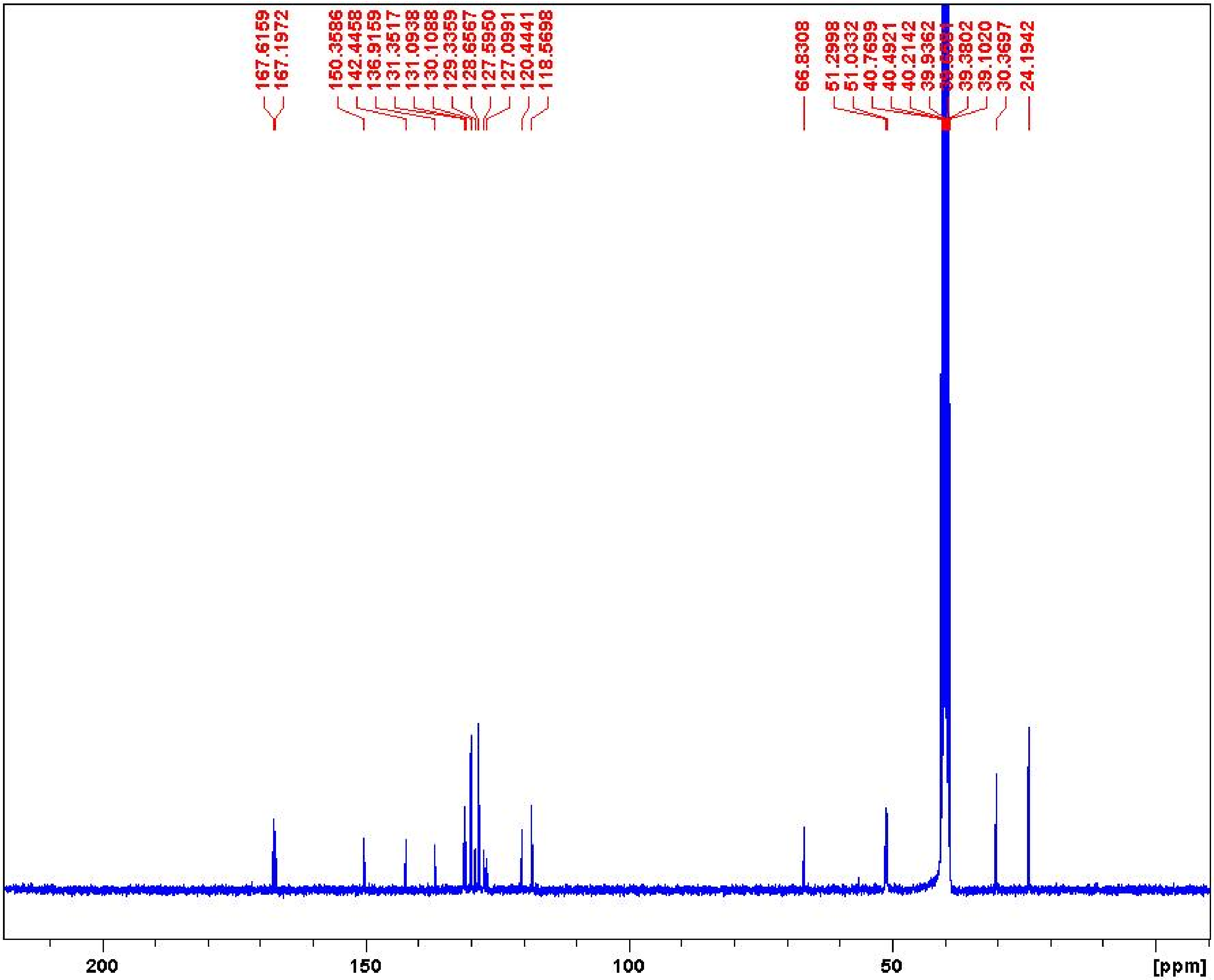

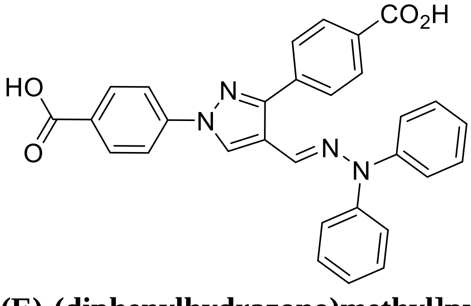

4-[1-(4-carboxyphenyl)-4-[(E)-(diphenylhydrazono)methyl]pyrazol-3-yl]benzoic acid (9)

Yellow solid (446 mg, 89%). ^{1}H NMR, 300 MHz (DMSO-d_6): δ 9.12 (s, 1H), 8.14-8.06 (m, 4H), 7.97 (d, J = 8.1 Hz, 2H), 7.70 (d, J = 8.1 Hz, 2H), 7.44 (t, J = 7.8 Hz, 4H), 7.28-7.14 (m, 7H); ^{13}C NMR (75MHz, DMSO-d6) δ = 167.4, 167.0, 150.5, 143.2, 142.4, 136.9, 131.3, 130.9, 130.4, 129.8, 129.1, 128.6, 128.0, 127.8, 125.0, 122.5, 119.5, 118.5.

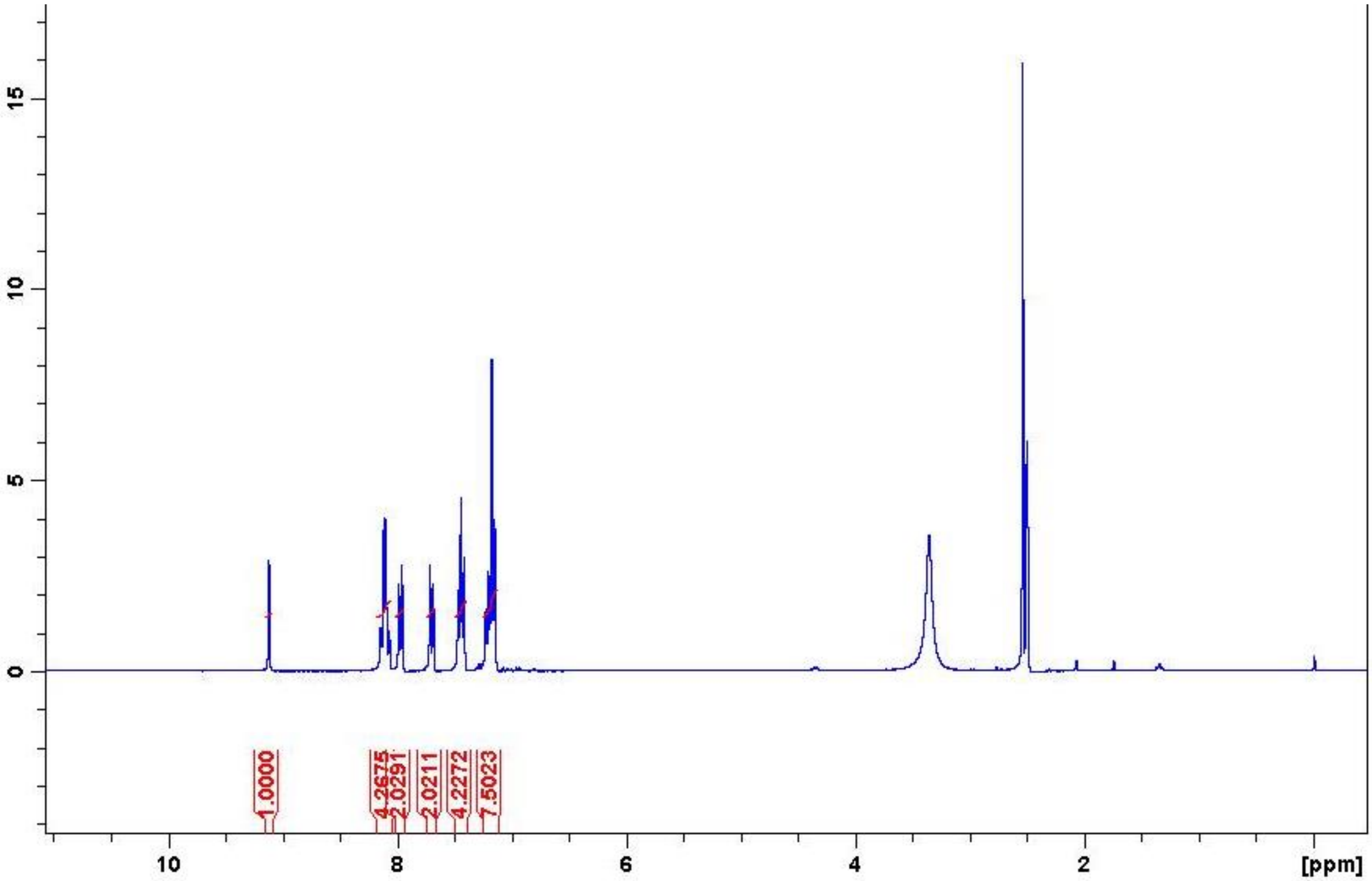

15
10
5
0
1.0000
4.2675
2.0291
2.0211
4.2272
7.5023
10
8
6
4
2
[ppm]

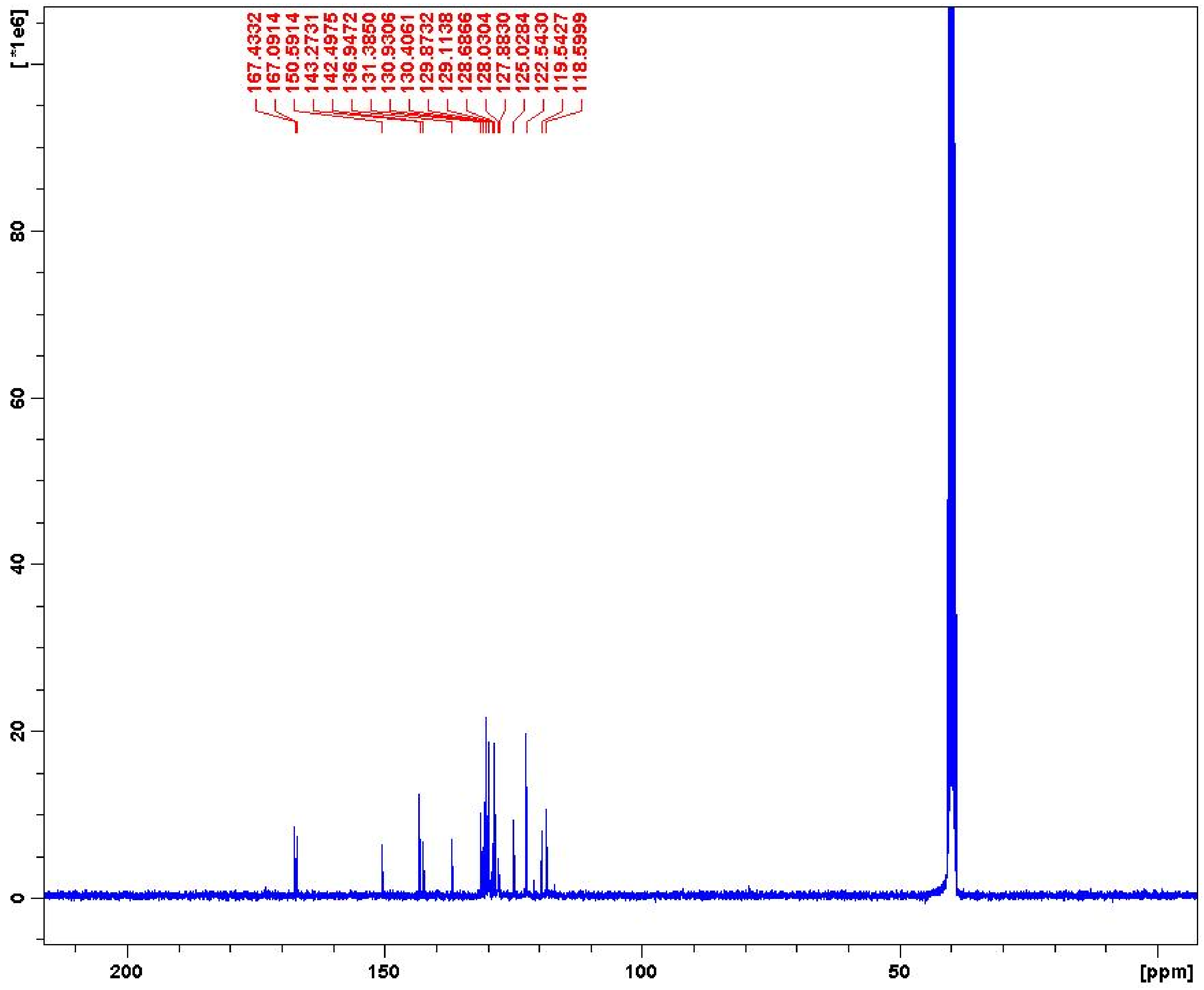

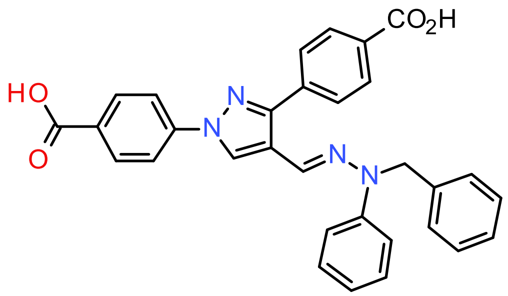

4-[4-[(E)-[benzyl(phenyl)hydrazono]methyl]-1-(4-carboxyphenyl)pyrazol-3-yl]benzoic acid (10)

Yellow solid (464 mg, 90%). (^{1}H NMR, 300 MHz (DMSO-d_6): δ 9.04 (s, 1H), 8.10-8.07 (m, 4H), 7.87 (d, J = 8.0 Hz, 2H), 7.47-7.35 (m, 9H), 7.30 (t, J = 7.9 Hz, 1H), 7.21 (d, J = 7.1 Hz, 2H), 6.91 (t, J = 7.2 Hz, 1H), 5.30 (s, 2H); ^{13}C NMR (75MHz, DMSO-d6) δ = 168.0, 167.7, 150.3, 147.7, 142.0, 136.5, 136.1, 132.5, 131.2, 130.0, 129.5, 129.3, 127.9, 127.5, 126.8, 126.6, 125.8, 120.5, 120.0, 118.4, 114.7, 48.9.

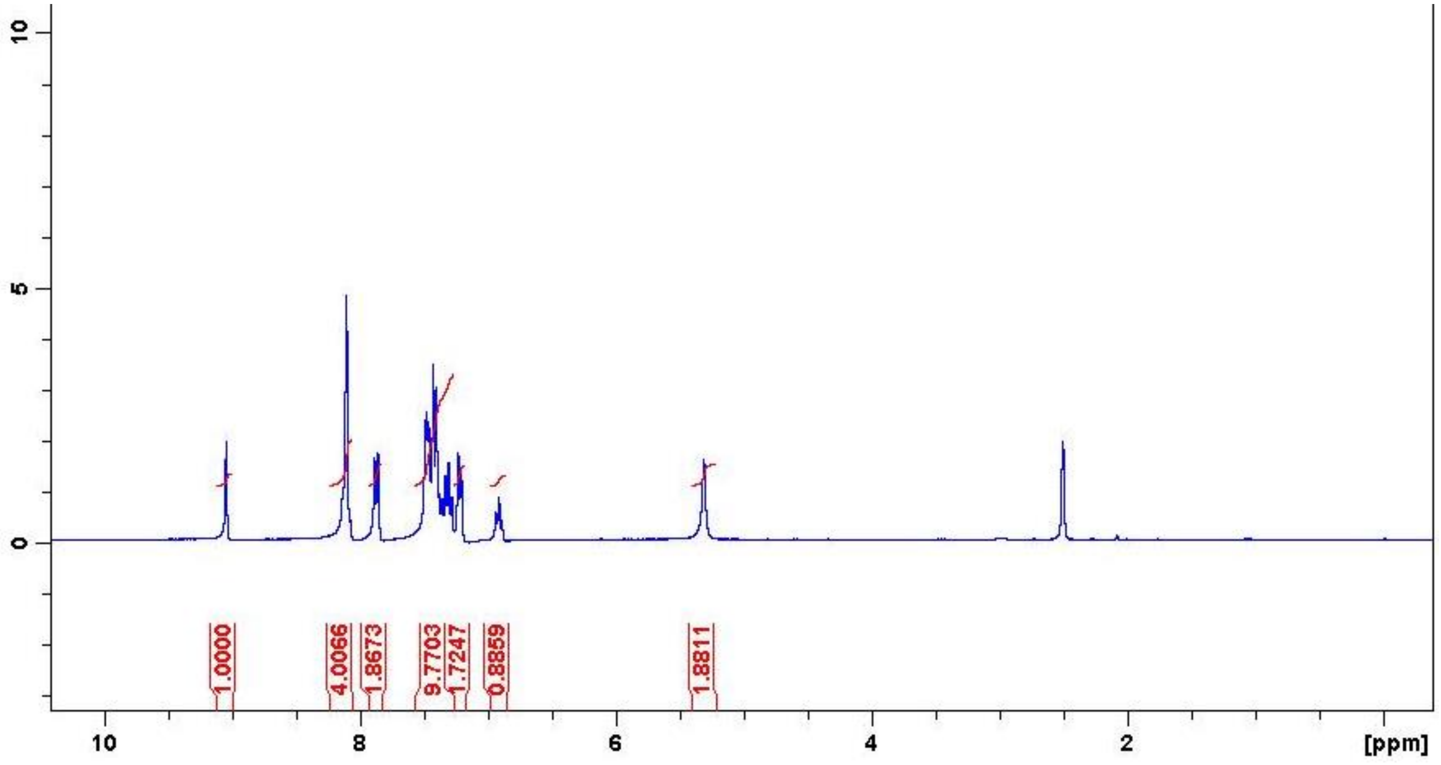
10
5
0
1.0000
4.0066
1.8673
9.7703
1.7247
0.8859
1.8811
10
8
6
4
2
[ppm]

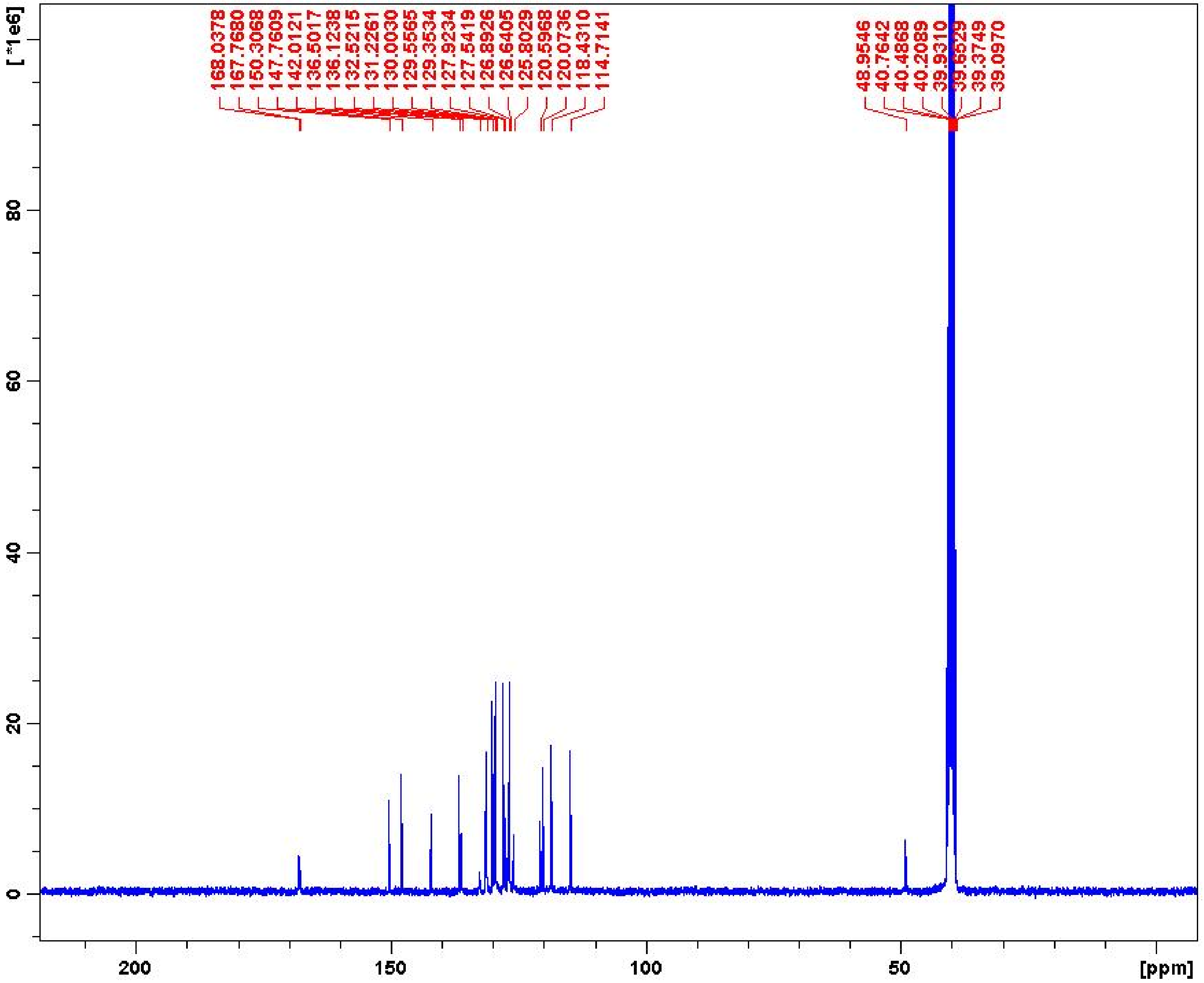

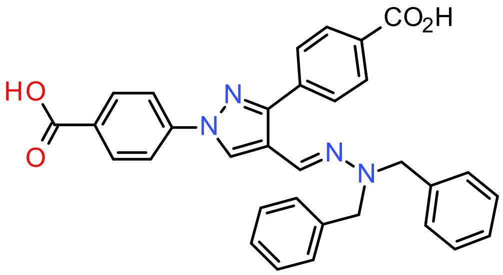

4-[1-(4-carboxyphenyl)-4-[(E)-(dibenzylhydrazono)methyl]pyrazol-3-yl]benzoic acid (11)

Yellow solid (487 mg, 92%). ^{1}H NMR, 300 MHz (DMSO-d_6): 8.79 (s, 1H), 8.11-8.03 (m, 4H), 7.82 (d, J = 8.1 Hz, 2H), 7.39-7.21 (m, 12H), 7.08 (s, 1H), 4.56 (s, 4H); ^{13}C NMR (75 MHz DMSO-d_6): 167.4, 167.1, 149.6, 142.5, 138.3, 136.8, 131.3, 130.4, 129.9, 129.0, 128.6, 127.9, 127.8, 127.5, 125.9, 123.9, 120.6, 118.5, 58.1.

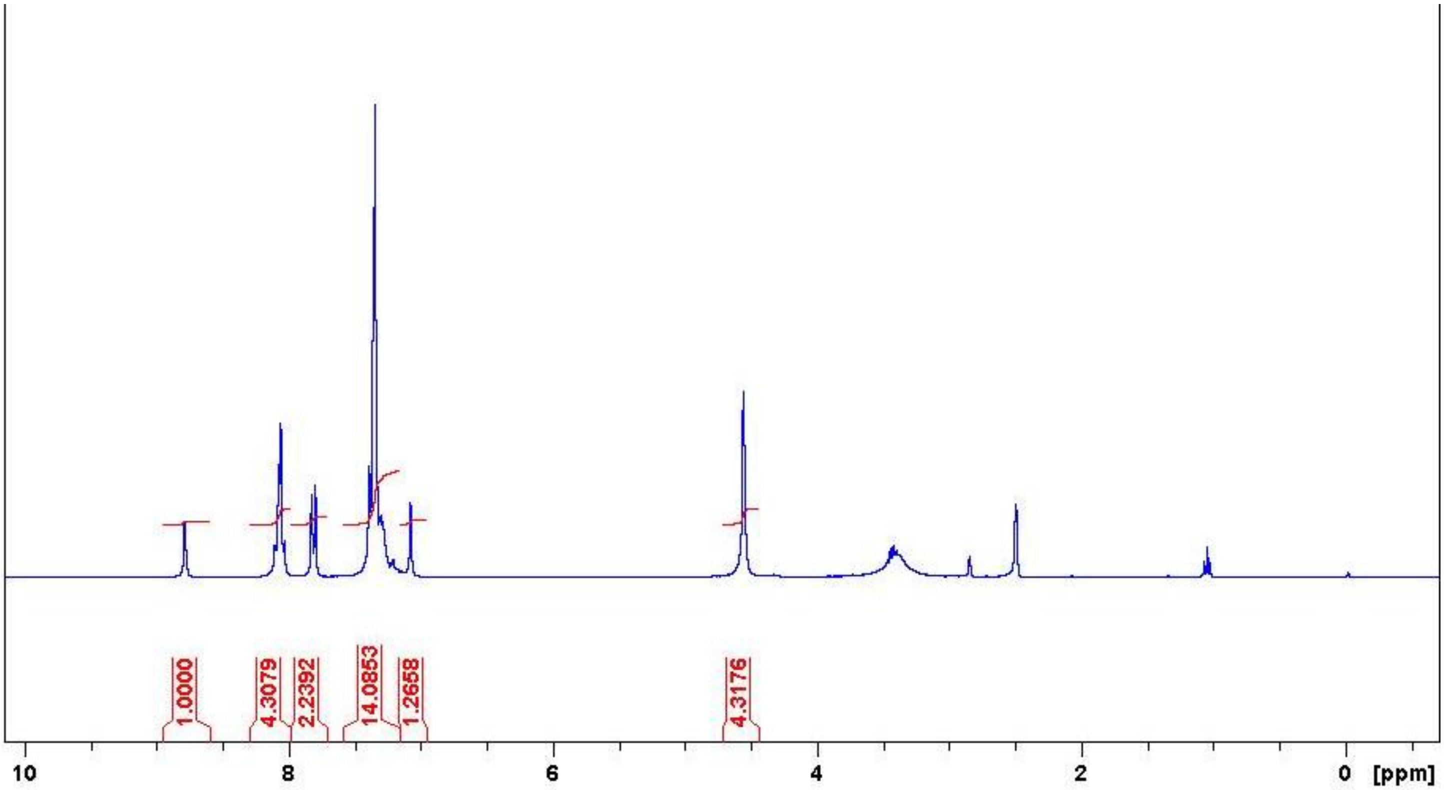

1.0000
4.3079
2.2392
14.0853
1.2658
4.3176
10
8
6
4
2
0
[ppm]

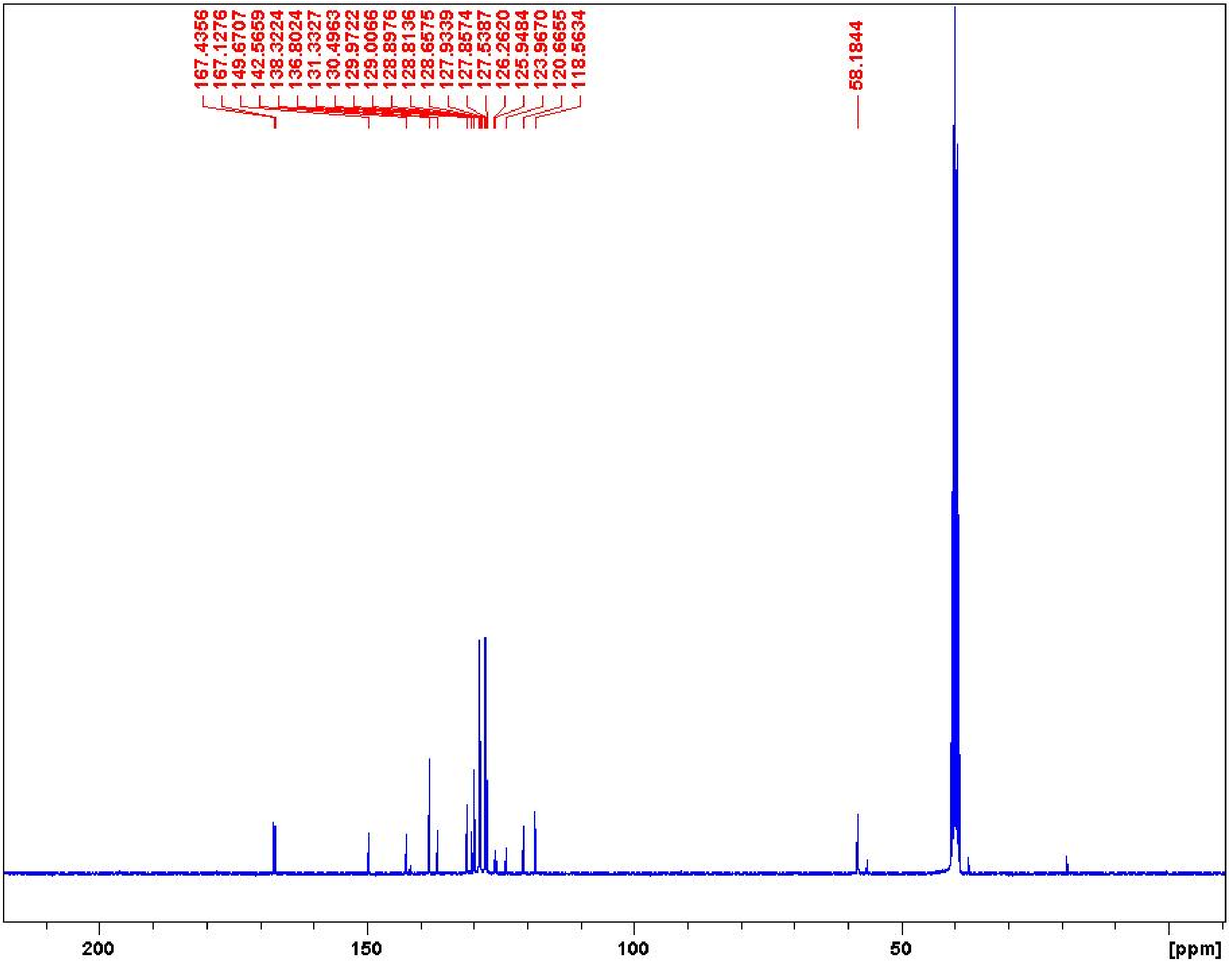

167.4356
167.1276
149.6707
142.5659
138.3224
136.8024
131.3327
130.4963
129.9722
129.0066
128.8976
128.8136
128.6575
127.9339
127.8574
127.5387
126.2620
125.9484
123.9670
120.6655
118.5634
58.1844
200
150
100
50
[ppm]

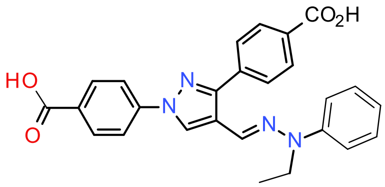

4-[1-(4-carboxyphenyl)-4-[(E)-[ethyl(phenyl)hydrazono]methyl]pyrazol-3-yl]benzoic acid (12)

Yellow solid (399 mg, 88%). ^{1}H NMR, 300 MHz (DMSO-d_6): 9.04 (s, 1H), 8.17-8.07 (m, 6H), 7.91 (m, 2H), 7.69 (s, 1H), 7.32-7.21 (m, 4H), 6.85 (t, 1H), 4.02 (q, J = 6.5 Hz, 2H), 1.13 (t, J = 6.6 Hz, 3H); ^{13}C NMR (75 MHz DMSO-d_6): 167.5, 167.1, 150.4, 146.5, 142.5, 137.3, 131.3, 130.8, 130.1, 129.4, 128.9, 128.8, 127.3, 123.7, 120.8, 120.1, 118.5, 114.4, 38.7, 9.9.

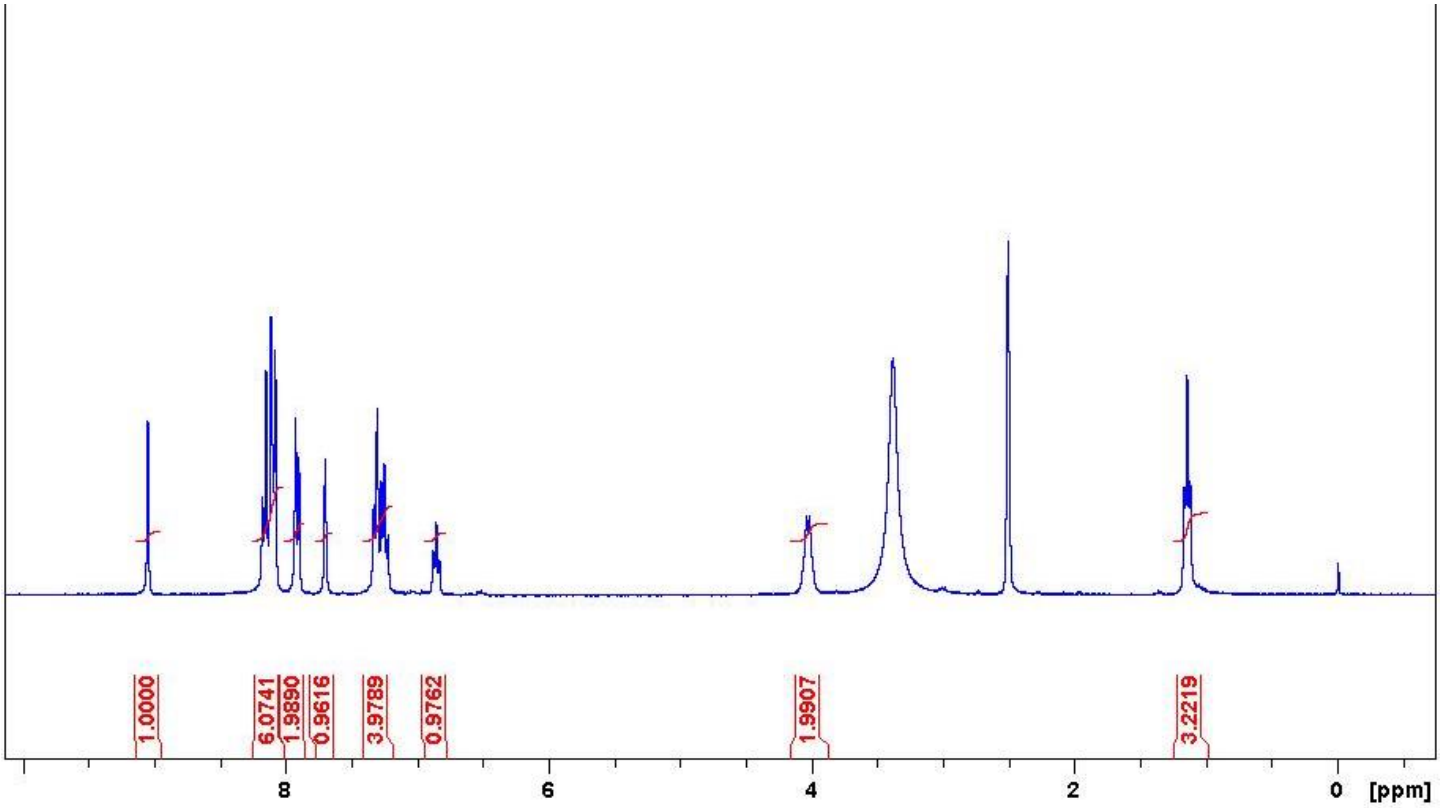

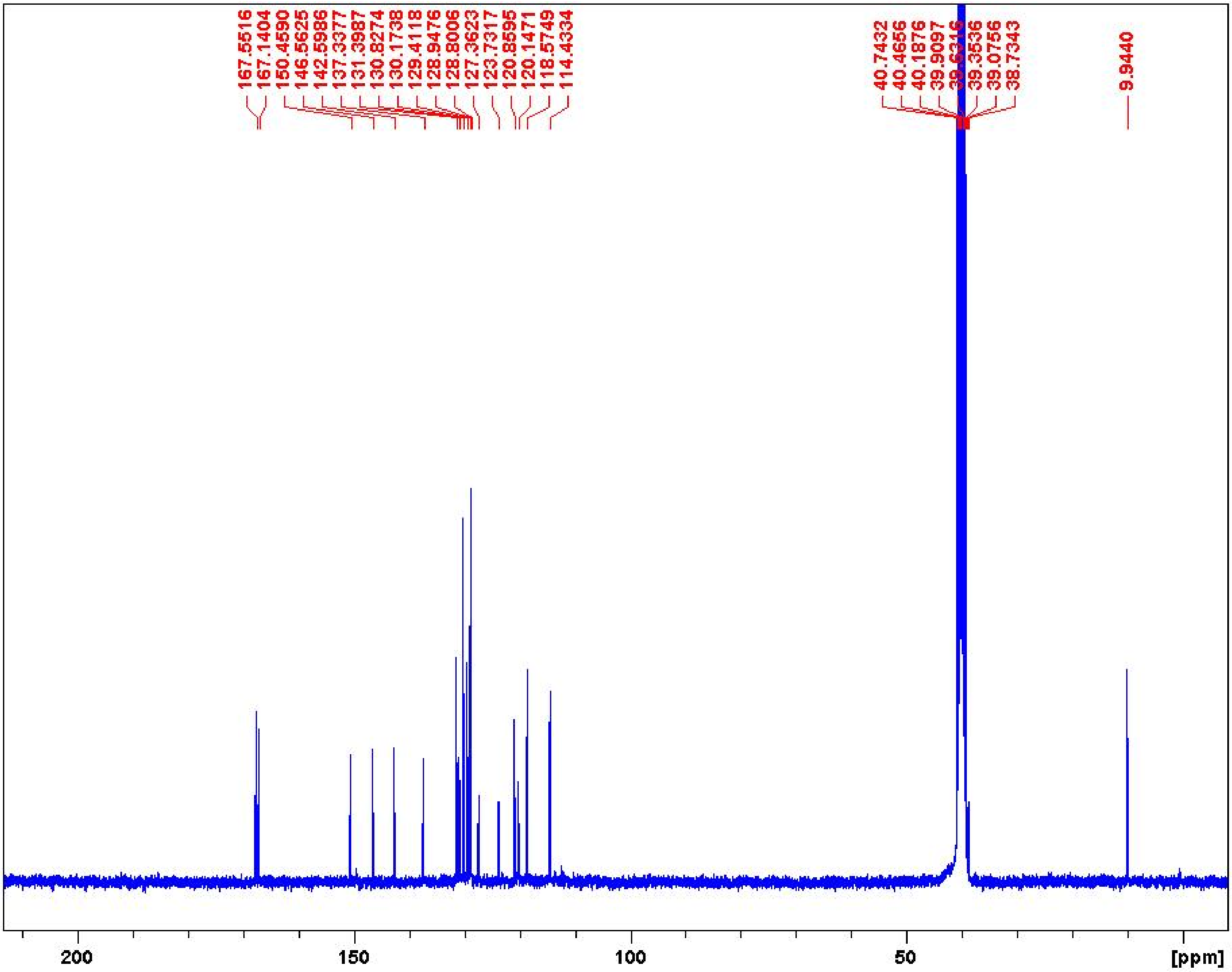

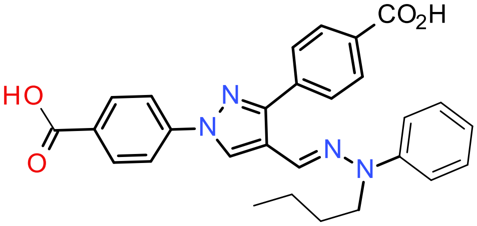

4-[4-[(E)-[butyl(phenyl)hydrazono]methyl]-1-(4-carboxyphenyl)pyrazol-3-yl]benzoic acid (13)

Yellow solid (428 mg, 89%). ^{1}H NMR, 300 MHz (DMSO-d_6): 9.06 (s, 1H), 8.17-8.05 (m, 6H), 7.89 (d, J = 8.2 Hz, 2H), 7.65 (s, 1H), 7.33-7.21 (m, 4H), 6.84 (t, J = 6.9 Hz, 1H), 3.92 (s, 2H), 1.55-1.53 (m, 2H), 1.42-1.34 (m, 2H), 0.93 (t, J = 7.1 Hz, 3H); ^{13}C NMR (75 MHz DMSO-d_6): 167.5, 167.1, 150.4, 146.9, 142.5, 137.2, 131.3, 130.8, 130.0, 129.3, 128.9, 128.7, 127.1, 123.7, 120.7, 120.1, 118.5, 114.4, 43.9, 26.4, 20.0, 14.2.

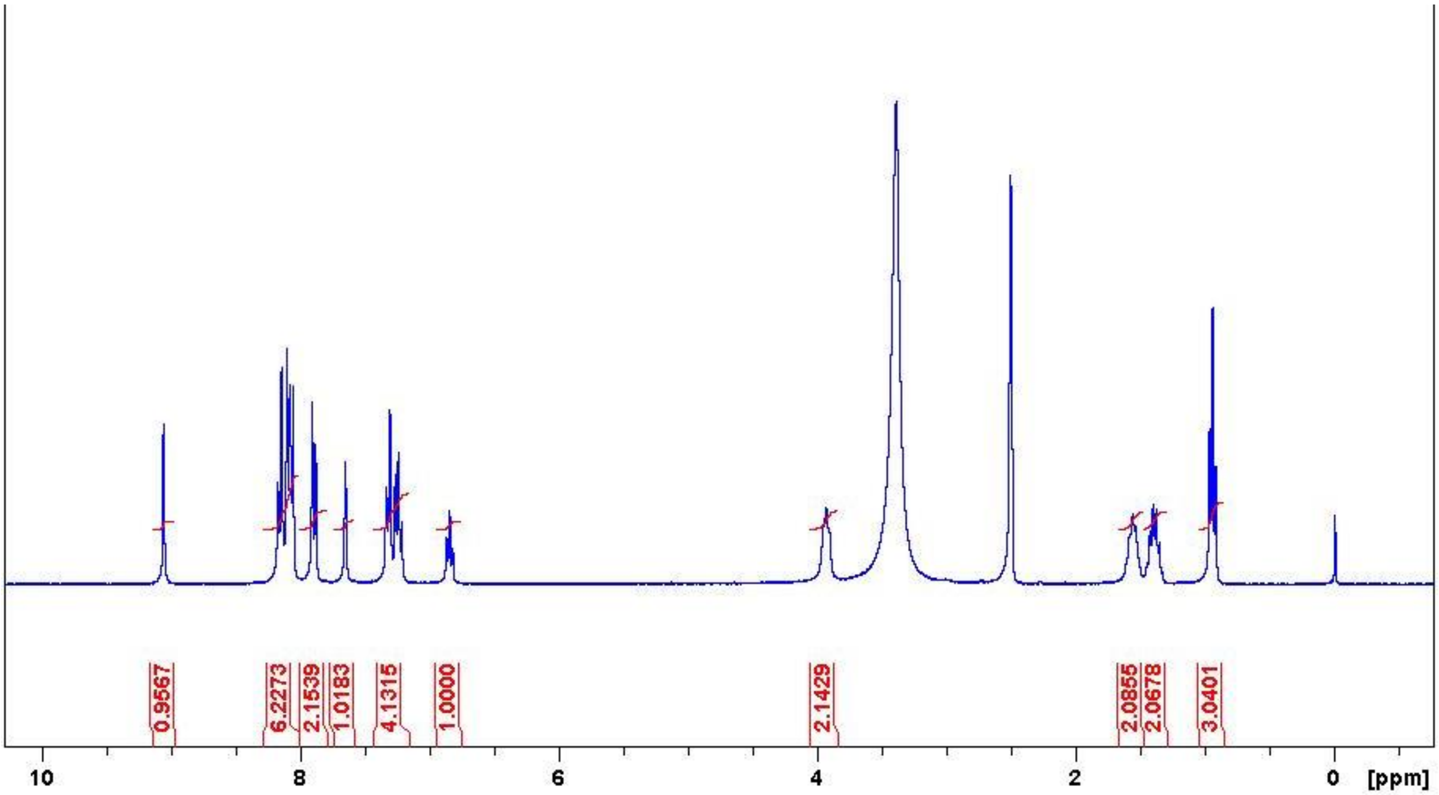
0.9567
6.2273
2.1539
1.0183
4.1315
1.0000
2.1429
2.0855
2.0678
3.0401
10
8
6
4
2
0
[ppm]

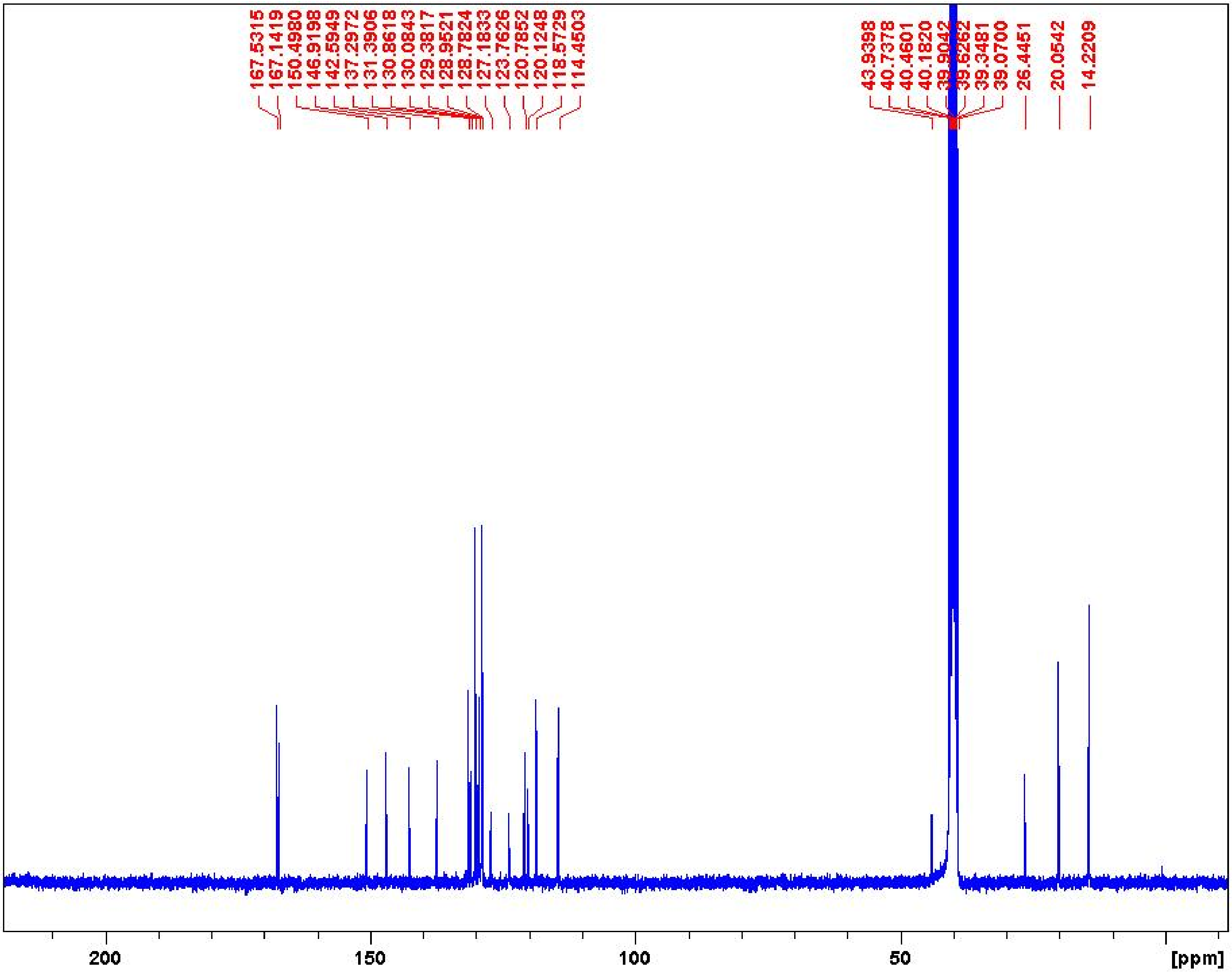

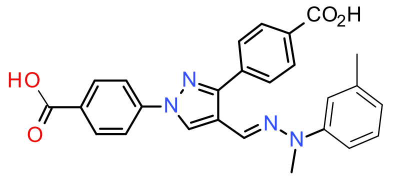

4-[1-(4-carboxyphenyl)-4-[(E)-[methyl(m-tolyl)hydrazono]methyl]pyrazol-3-yl]benzoic acid (14)

Yellow solid (408 mg, 90%). ^{1}H NMR (300 MHz DMSO-d_6): δ 9.00 (s, 1H), 8.15-8.06 (m, 6H), 7.94 (d, J = 8.1 Hz, 2H), 7.64 (s, 1H), 7.13-7.09 (m, 3H), 6.66 (s, 1H), 3.37 (s, 3H), 2.25 (s, 3H); ^{13}C NMR (75MHz, DMSO-d_6) δ = 167.5, 167.1, 150.3, 147.6, 142.5, 138.4, 137.4, 131.4, 130.7, 130.1, 129.1, 128.9, 128.8, 127.6, 124.3, 121.2, 120.7, 118.5, 115.5, 112.2, 32.9, 21.9.

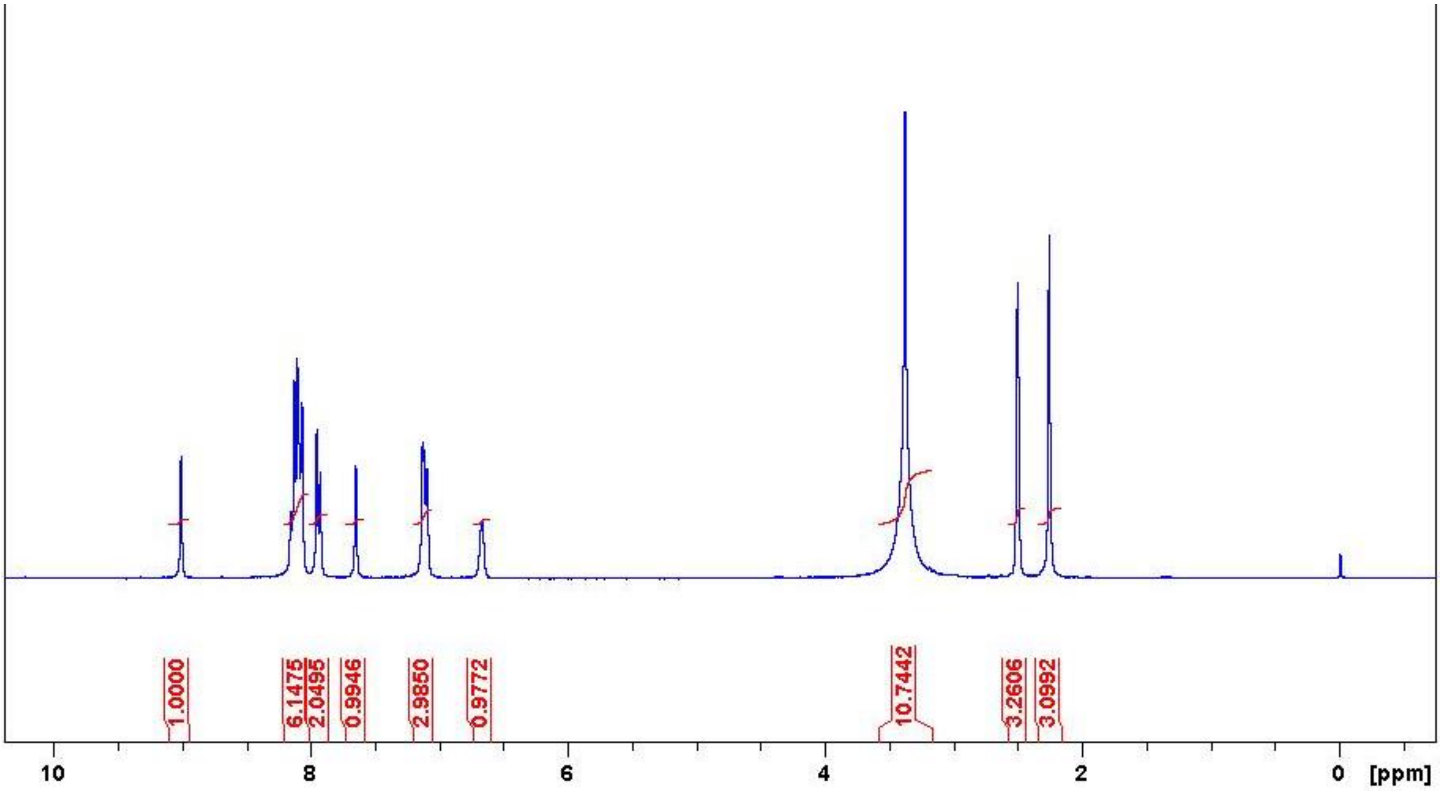
1.0000
6.1475
2.0495
0.9946
2.9850
0.9772
10.7442
3.2606
3.0992
10
8
6
4
2
0
[ppm]

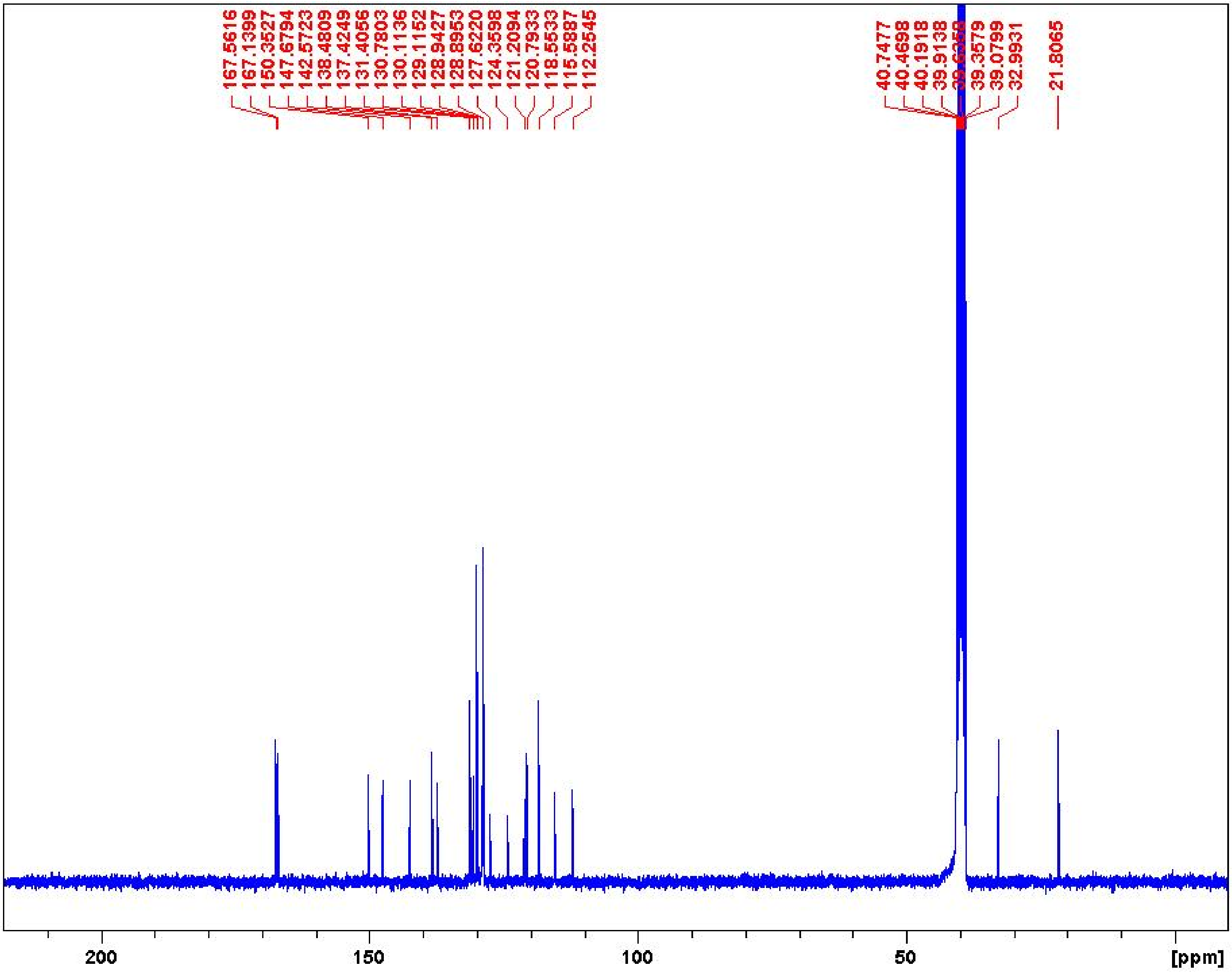

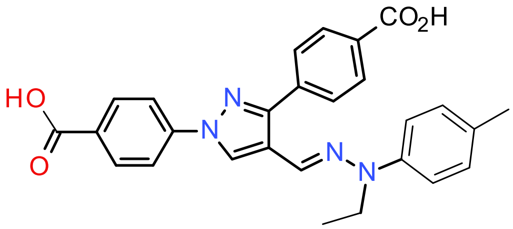

4-[1-(4-carboxyphenyl)-4-[(E)-[ethyl(p-tolyl)hydrazono]methyl]pyrazol-3-yl]benzoic acid (15)

Yellow solid (411 mg, 88%). ^{1}H NMR, 300 MHz (DMSO-d_6): δ 9.01 (s, 1H), 8.18-8.06 (m, 6H), 7.90 (d, J = 8.1 Hz, 2H), 7.63 (s, 1H), 7.20 (d, J = 8.3 Hz, 2H), 7.05 (d, J = 8.3 Hz, 2H), 3.98 (q, J = 6.4 Hz, 2H), 2.23 (s, 3H), 1.11 (t, J = 6.3 Hz, 3H); ^{13}C NMR (75MHz, DMSO-d_6) δ = 167.5, 167.1, 150.3, 144.4, 142.6, 137.3, 131.3, 130.7, 130.1, 129.8, 128.9, 128.8, 128.7, 127.2, 123.0, 120.9, 118.5, 114.6, 20.5, 9.9.

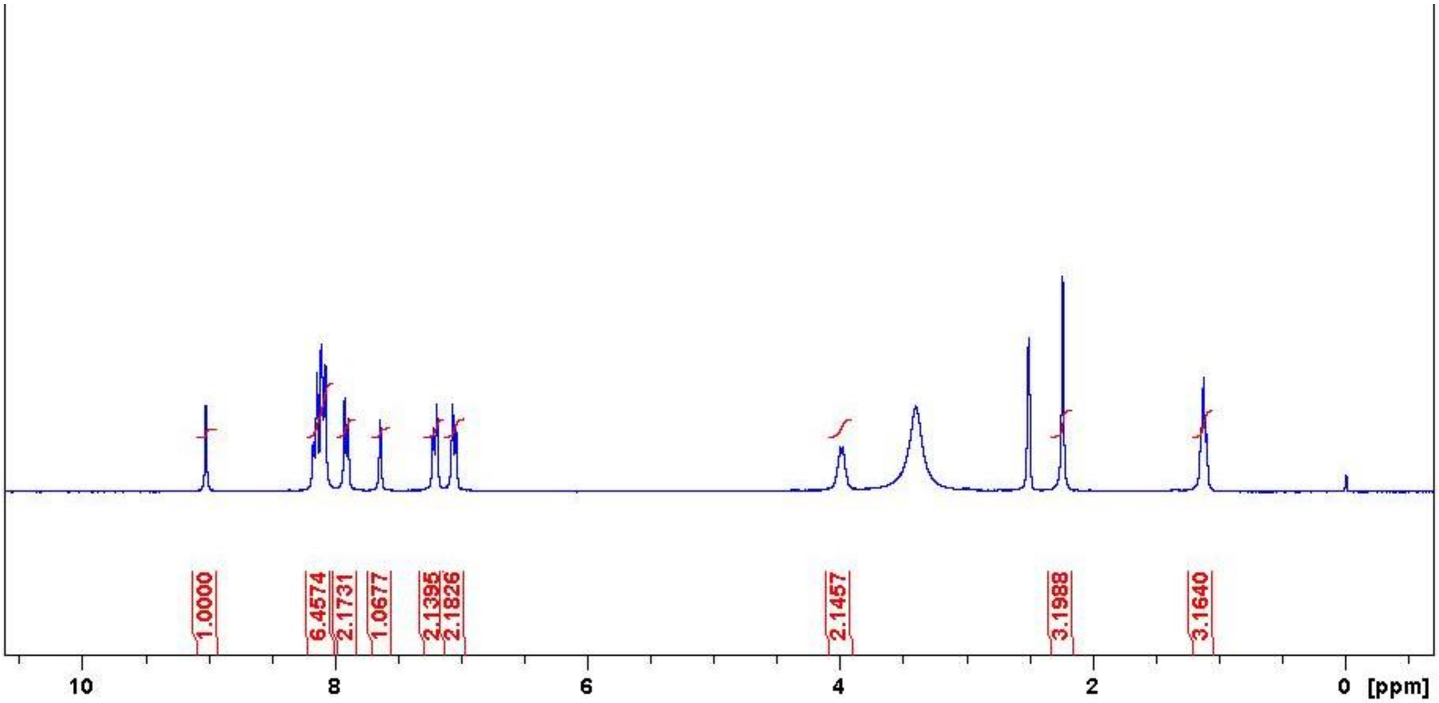
1.0000
6.4574
2.1731
1.0677
2.1395
2.1826
2.1457
3.1988
3.1640
10
8
6
4
2
0 [ppm]

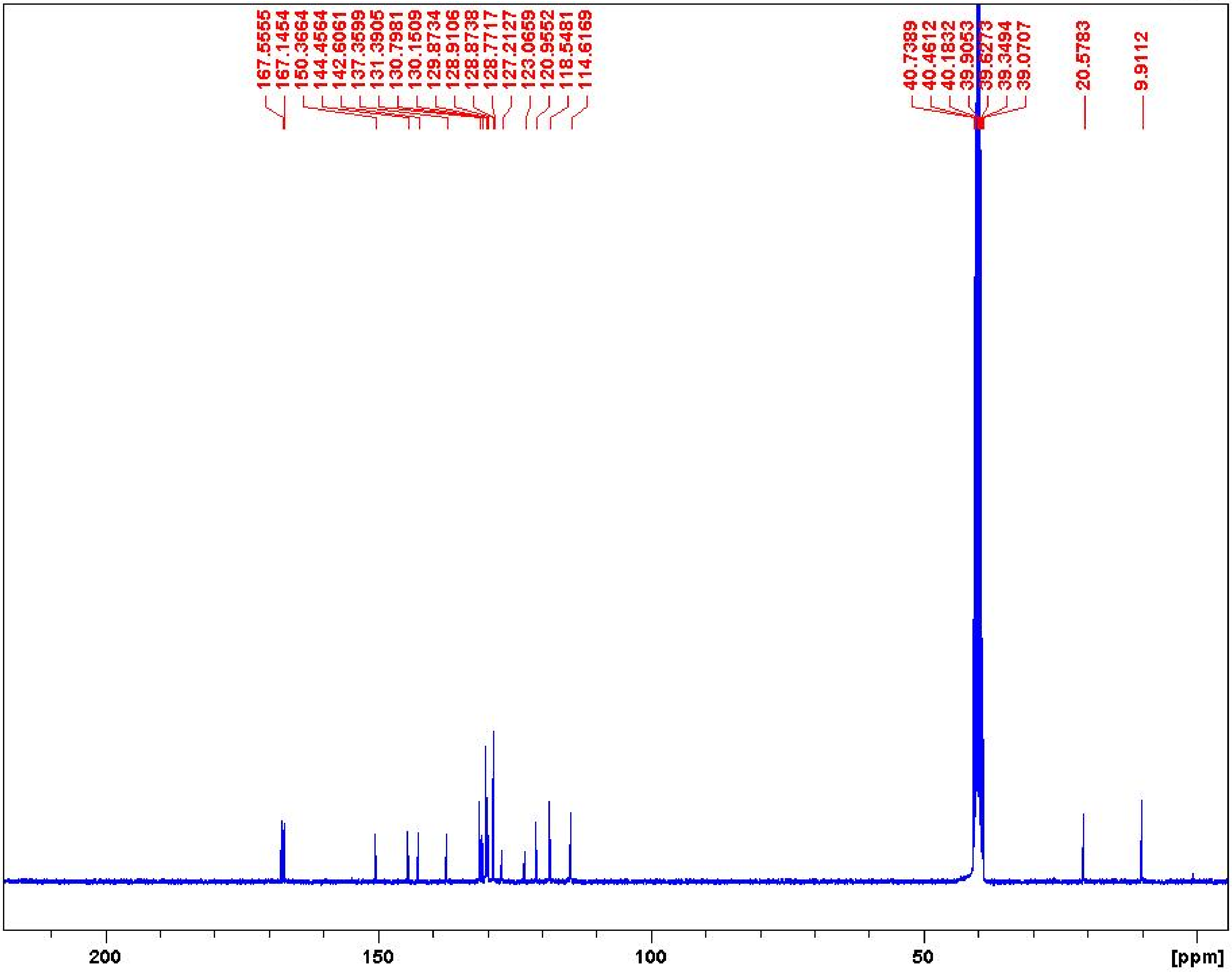
167.5555
167.1454
150.3664
144.4564
142.6061
137.3599
131.3905
130.7981
130.1509
129.8734
128.9106
128.8738
128.7717
127.2127
123.0659
120.9552
118.5481
114.6169
40.7389
40.4612
40.1832
39.9053
39.6273
39.3494
39.0707
20.5783
9.9112
200
150
100
50
[ppm]

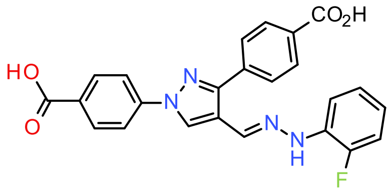

4,4'-(4-{(*E*)-[2-(2-fluorophenyl)hydrazinylidene]methyl}-1*H*-pyrazole-1,3-diyl)dibenzoic acid (16)

Brownish solid (346 mg, 78%). ^{1}H NMR, 300 MHz (DMSO-d_6): δ 10.52 (s, 1H), 9.12 (s, 1H), 8.16-8.07 (m, 6H), 7.99 (s, 1H), 7.90 (d, J = 8.1 Hz, 2H), 7.22-7.15 (m, 1H), 6.86 (d, J = 11.7 Hz, 1H), 6.73 (d, J = 8.1 Hz, 1H), 6.49 (t, J = 6.7 Hz, 1H); ^{13}C NMR (75MHz, DMSO-d_6) δ = 167.5, 167.1, 163.8 ($^{1}J_{C\text{-}F}$ = 238.8 Hz), 150.4, 147.7 ($^{3}J_{C\text{-}F}$ = 11.1 Hz), 142.4, 136.9, 131.3, 131.03 (d, $^{3}J_{C\text{--}F}$ = 10.0 Hz), 131.0, 130.1, 130.0, 129.1, 128.8, 127.7, 119.6, 118.6, 108.4, 104.5 ($^{3}J_{C\text{-}F}$ = 21.2 Hz), 98.9 ($^{3}J_{C\text{-}F}$ = 26.0 Hz).

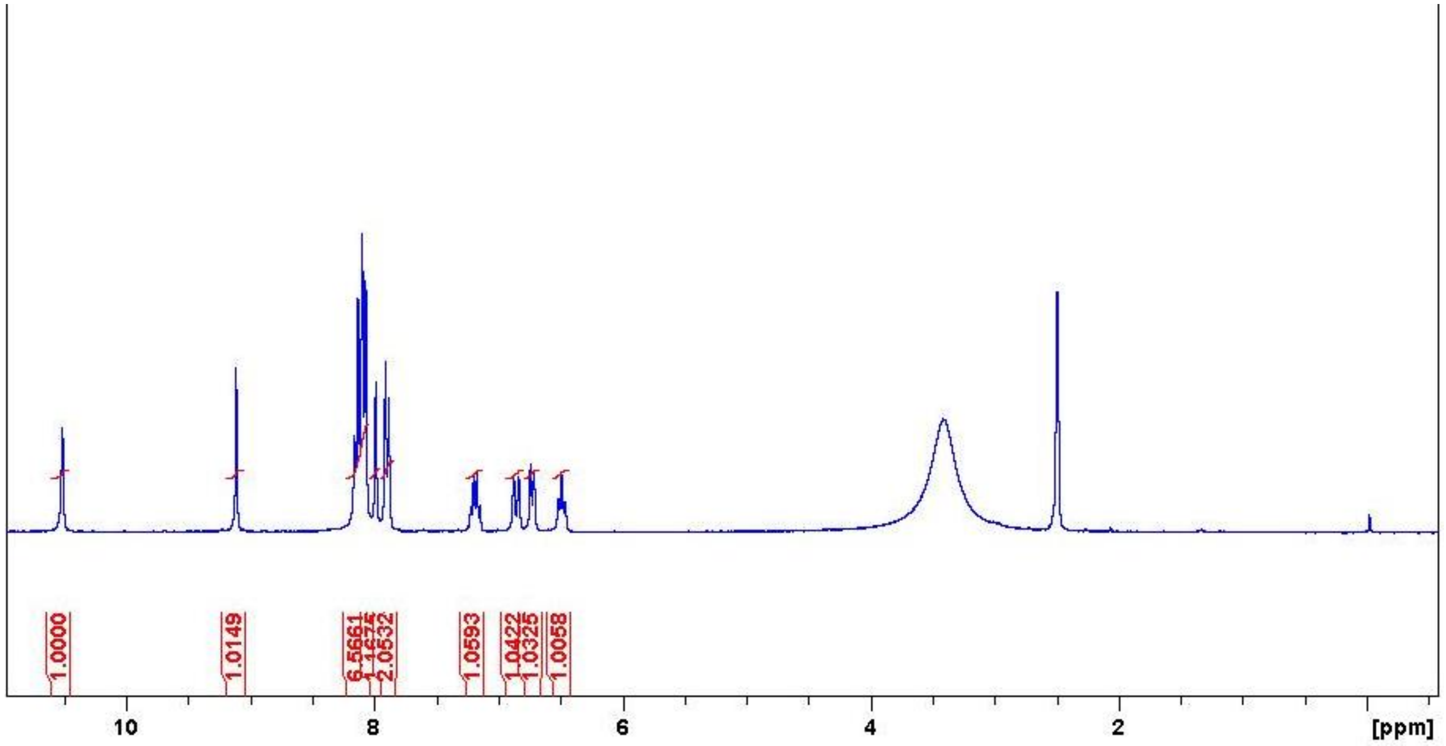

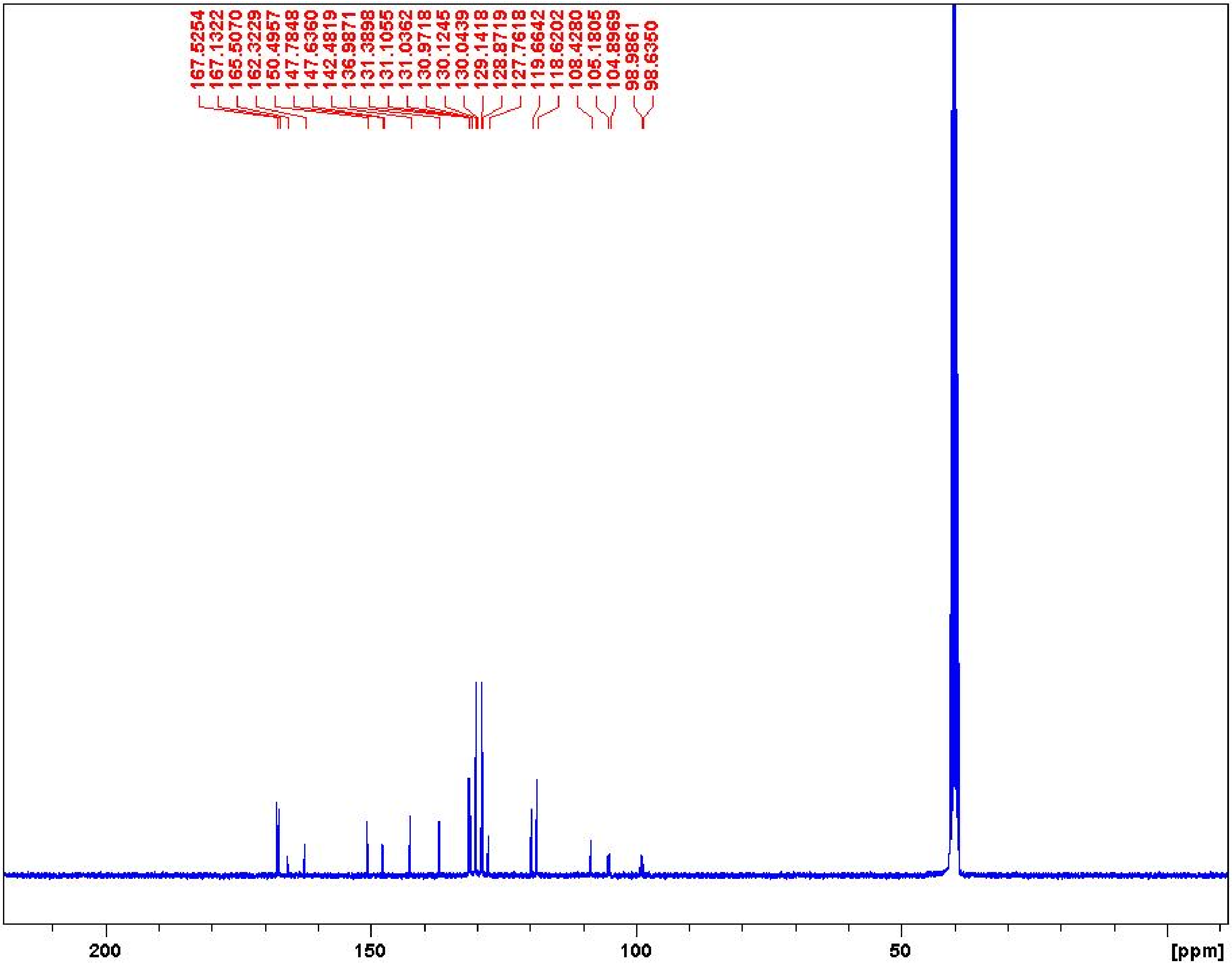

4,4'-(4-{(*E*)-[2-(3-fluorophenyl)hydrazinylidene]methyl}-1*H*-pyrazole-1,3-diyl)dibenzoic acid (17)

Brownish solid (346 mg, 78%). ^{1}H NMR, 300 MHz (DMSO-d$_6$): δ 10.47 (s, 1H), 9.08 (s, 1H), 8.15-7.90 (m, 4H), 7.97 (s, 1H), 7.75 (d, J = 7.4 Hz, 2H), 7.55-7.48 (m, 2H), 7.19 (q, J = 6.8 Hz, 1H), 6.88 (d, J = 11.7 Hz, 1H), 6.74 (d, J = 8.0 Hz, 1H), 6.49 (t, J = 8.3 Hz, 1H); ^{13}C NMR (75MHz, DMSO-d$_6$) δ = 167.1, 163.9 (d, ^{1}J =238.6 Hz), 151.6, 147.8 (d, ^{3}J = 11.1 Hz), 142.6, 132.7, 131.3, 131.0 (d, ^{3}J = 10.1 Hz), 130.4, 129.0, 128.8, 127.2, 119.2, 118.9, 118.4, 108.4, 105.0 (^{2}J = 20.5), 104.7, 98.8 (d, 2J = 26.1 Hz).

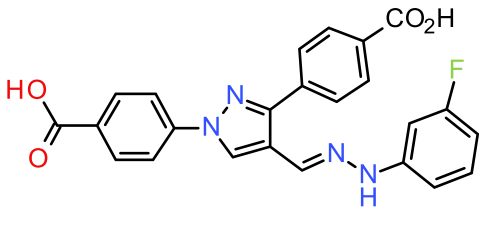

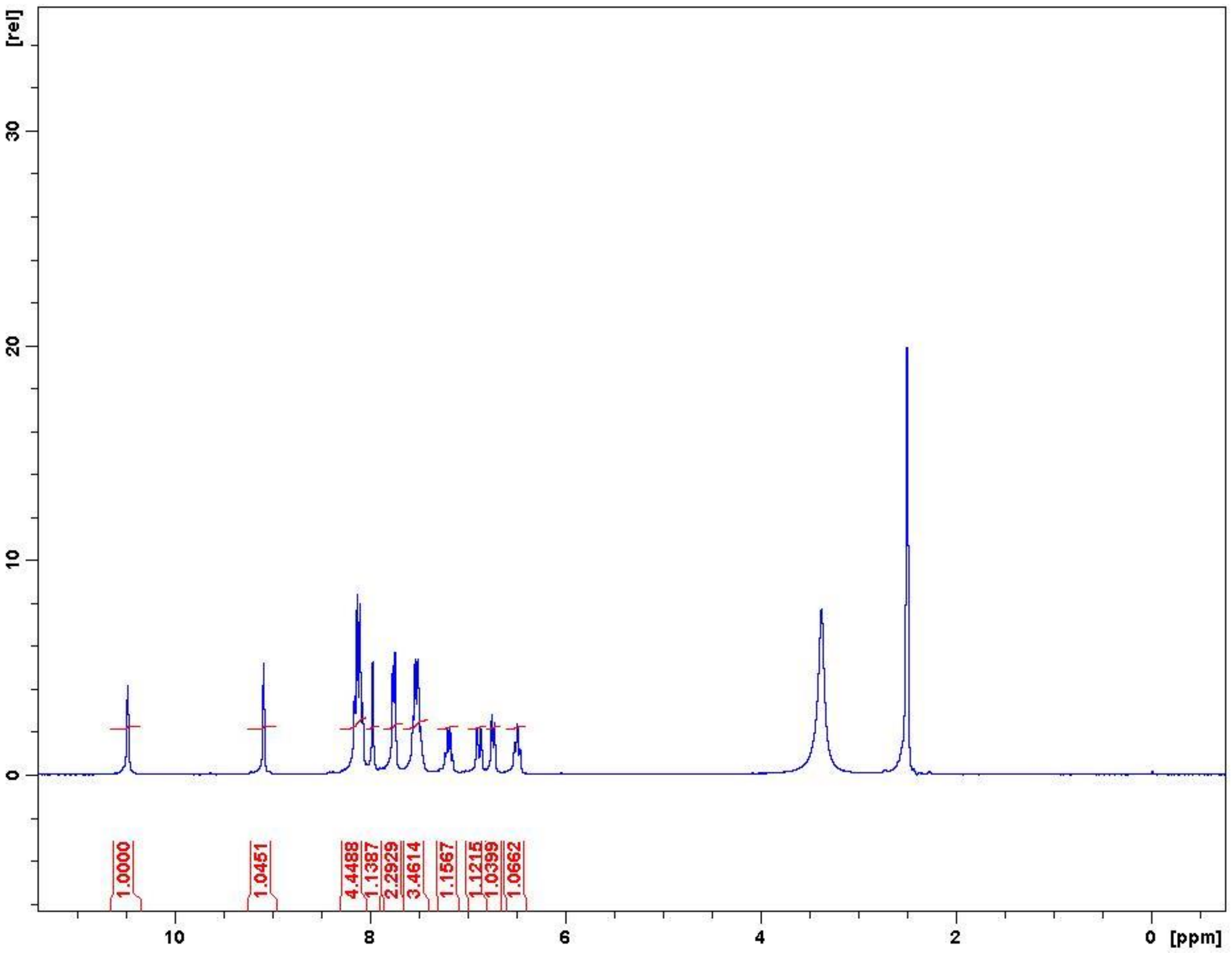
[rel]
30
20
10
0
1.0000
1.0451
4.4488
1.1387
2.2929
3.4614
1.1567
1.1215
1.0399
1.0662
10
8
6
4
2
0 [ppm]

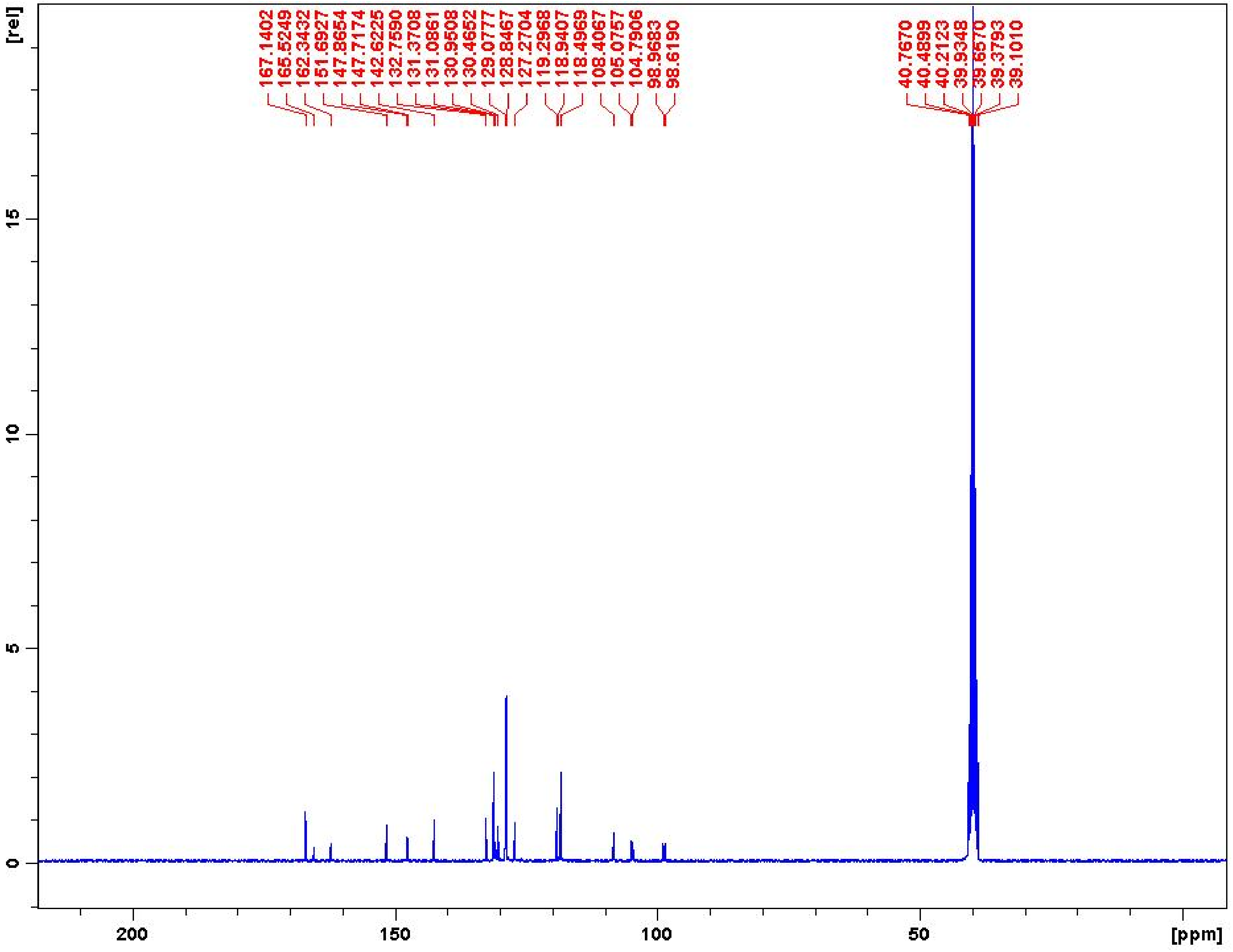

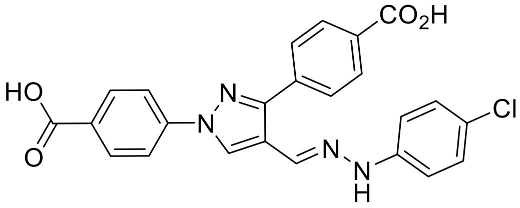

4,4'-(4-{(*E*)-[2-(4-chlorophenyl)hydrazinylidene]methyl}-1*H*-pyrazole-1,3-diyl)dibenzoic acid (18)

Brownish solid (349 mg, 76%). ^{1}H NMR, 300 MHz (DMSO-d$_6$): δ 10.42 (s, 1H), 9.07 (s, 1H), 8.16-8.07 (m, 6H), 7.98 (s, 1H), 7.90 (d, J = 8.2 Hz, 2H), 7.22 (d, J = 8.7 Hz, 2H), 7.02 (d, J = 8.7 Hz, 2H); ^{13}C NMR (75MHz, DMSO-d$_6$) δ = 167.5, 167.1, 150.4, 144.6, 142.4, 136.9, 131.3, 131.0, 130.0, 129.8, 129.7, 128.8, 127.5, 122.1, 119.7, 118.6, 113.6.

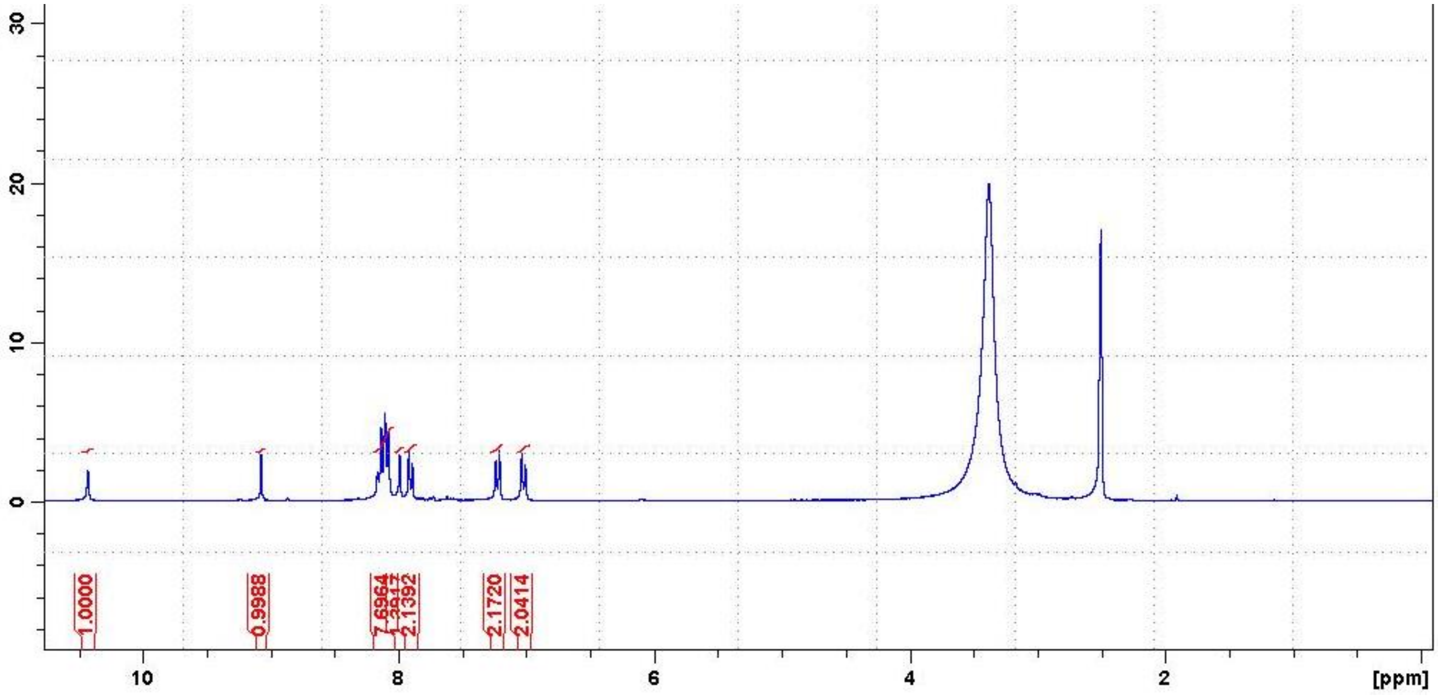

30
20
10
0
1.0000
0.9988
7.6964
1.3617
2.1392
2.1720
2.0414
10
8
6
4
2
[ppm]

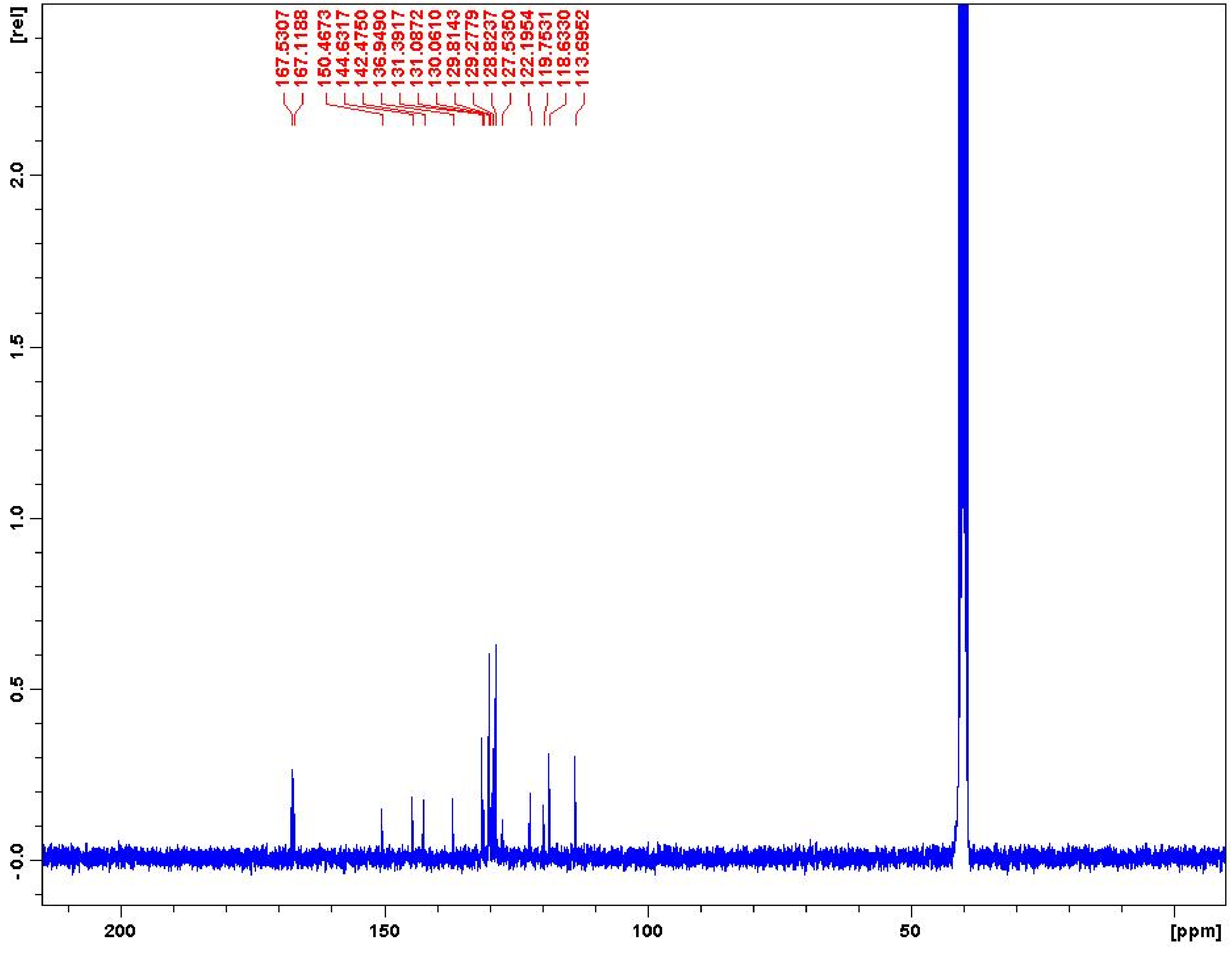

[rel]
167.5307
167.1188
150.4673
144.6317
142.4750
136.9490
131.3917
131.0872
130.0610
129.8143
129.2779
128.8237
127.5350
122.1954
119.7531
118.6330
113.6952
2.0
1.5
1.0
0.5
-0.0
200
150
100
50
[ppm]

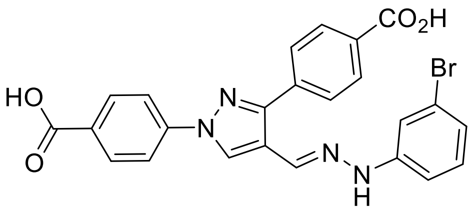

4,4'-(4-{(*E*)-[2-(3-bromophenyl)hydrazinylidene]methyl}-1*H*-pyrazole-1,3-diyl)dibenzoic acid (19)

Brownish solid (388 mg, 77%). ^{1}H NMR, 300 MHz (DMSO-d_6): δ 10.47 (s, 1H), 9.22 (s, 1H), 8.15-8.08 (m, 2H), 7.99 (s, 1H), 7.91 (d, J = 8.3 Hz, 2H), 7.21-7.20 (m, 1H), 7.11 (t, J = 7.9 Hz, 1H), 6.91-6.84 (m, 2H); ^{13}C NMR (75MHz, DMSO-d_6) δ = 167.5, 167.1, 150.5, 147.2, 142.4, 136.9, 131.3, 131.1, 130.9, 130.5, 130.0, 129.3, 128.9, 127.9, 123.0, 121.2, 119.5, 118.6, 114.3, 111.3.

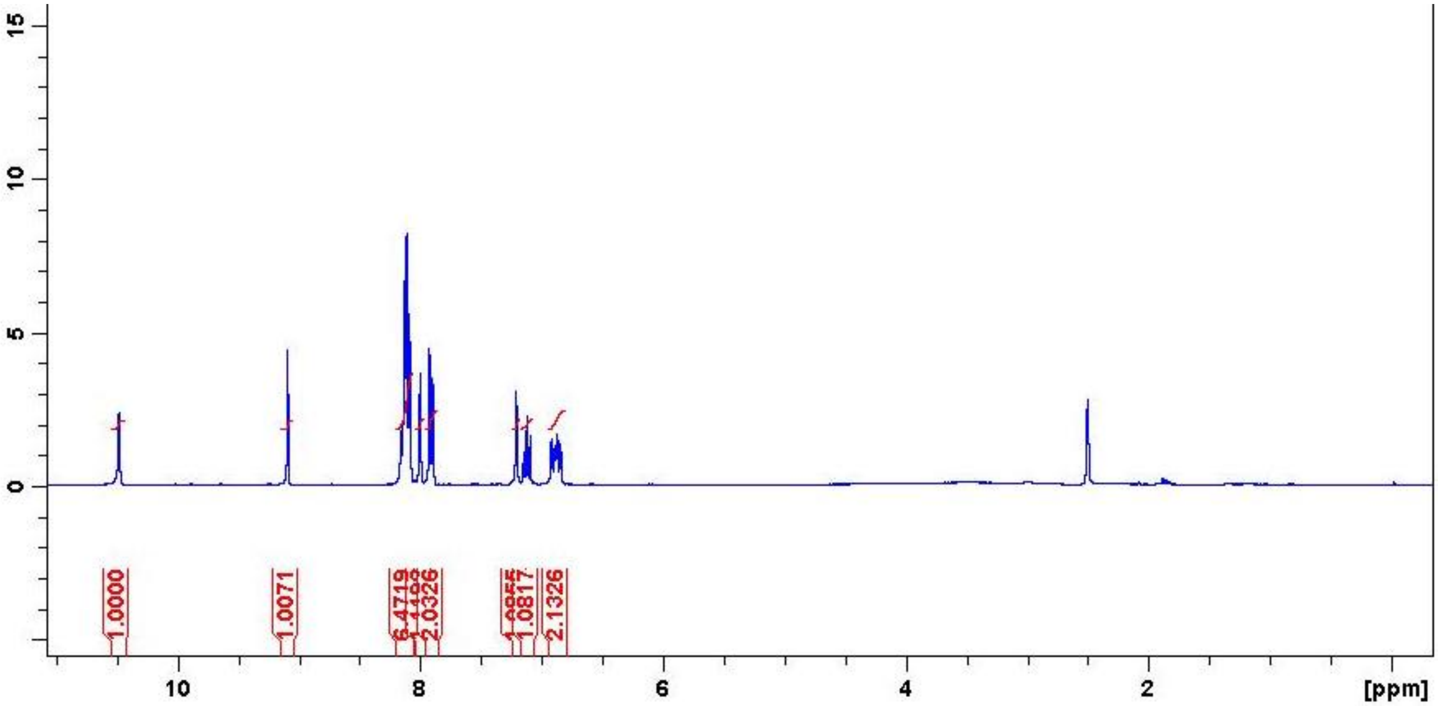

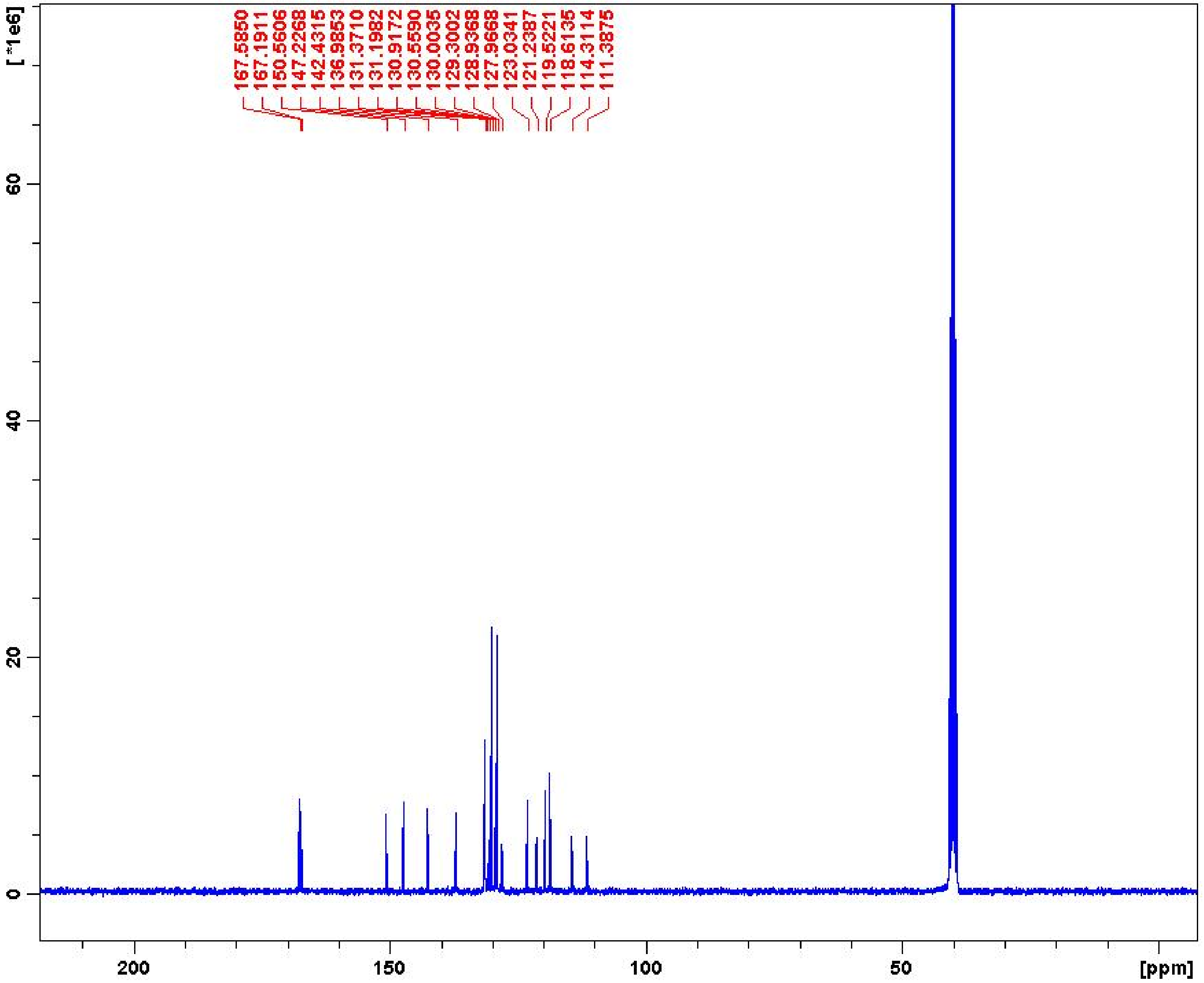

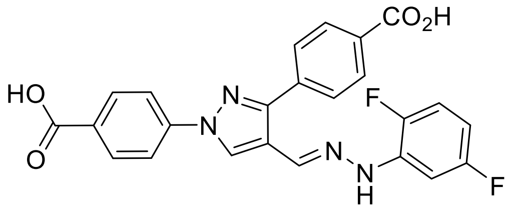

4,4'-(4-{(*E*)-[2-(2,5-difluorophenyl)hydrazinylidene]methyl}-1*H*-pyrazole-1,3-diyl)dibenzoic acid (20)

Brownish solid (337 mg, 73%). ^{1}H NMR, 300 MHz (DMSO-d_6): δ 10.42 (s, 1H), 9.18 (s, 1H), 8.33 (s, 1H), 8.17-8.07 (m, 6H), 7.89 (d, J = 8.2 Hz, 2H), 7.25-7.10 (m, 2H), 6.52-6.47 (m, 1H); ^{13}C NMR (75MHz, DMSO-d_6) δ = 167.5, 167.1, 160.1 ($^1J_{C\text{-}F}$ = 236.4 Hz), 150.6, 145.5 (d, J = 234.9 Hz), 142.4, 136.8, 135.3-135.1 (m), 133.2, 131.3, 131.1, 130.0, 129.3, 128.8, 127.9, 119.4, 118.6, 118.6-116.1 (m), 104.0 ($^{2,3}J_{C\text{-}F}$ = 25.0, 7.5 Hz), 100.8 ($^2J_{C\text{-}F}$ = 30.4 Hz).

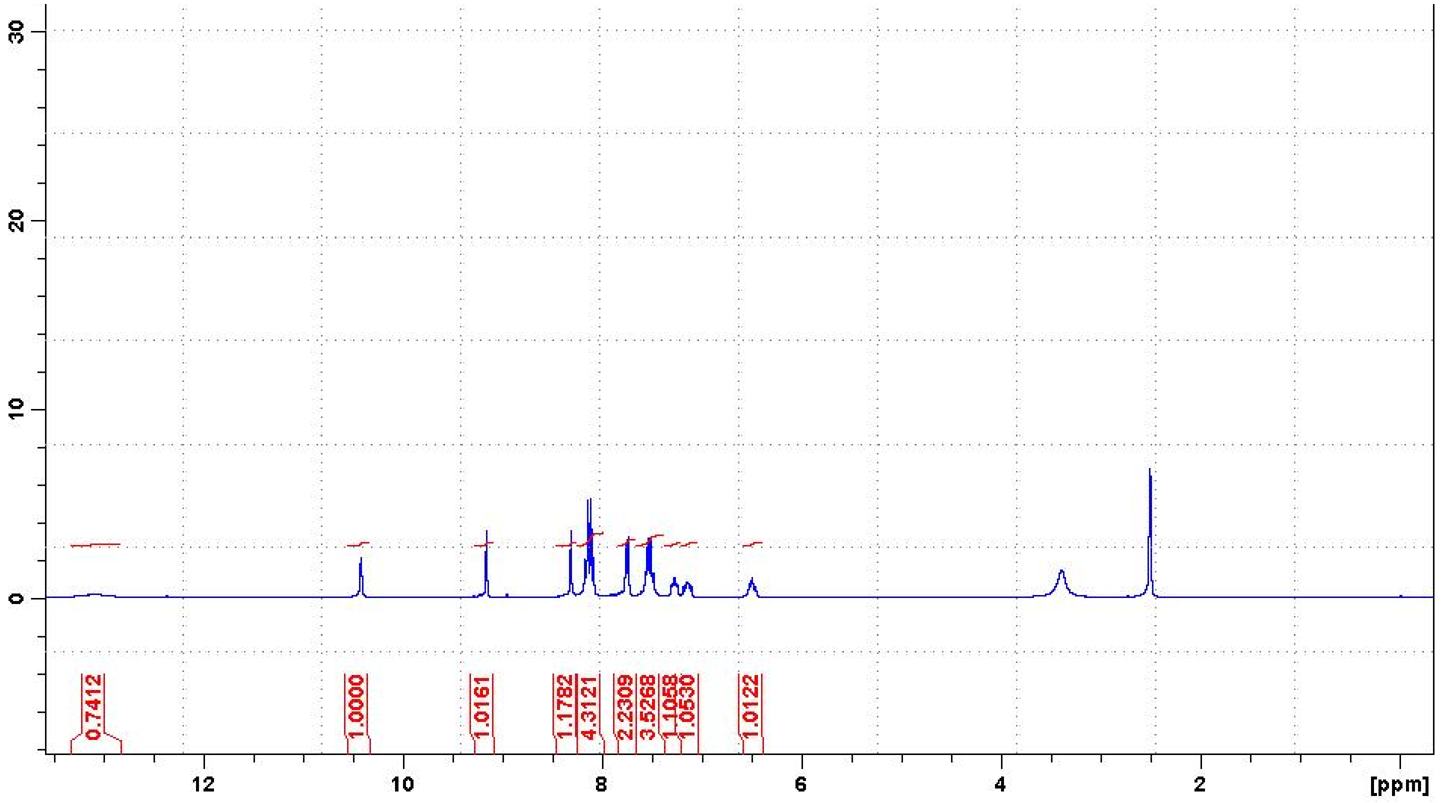

0.7412
1.0000
1.0161
1.1782
4.3121
2.2309
3.5268
1.1058
1.0530
1.0122
[ppm]

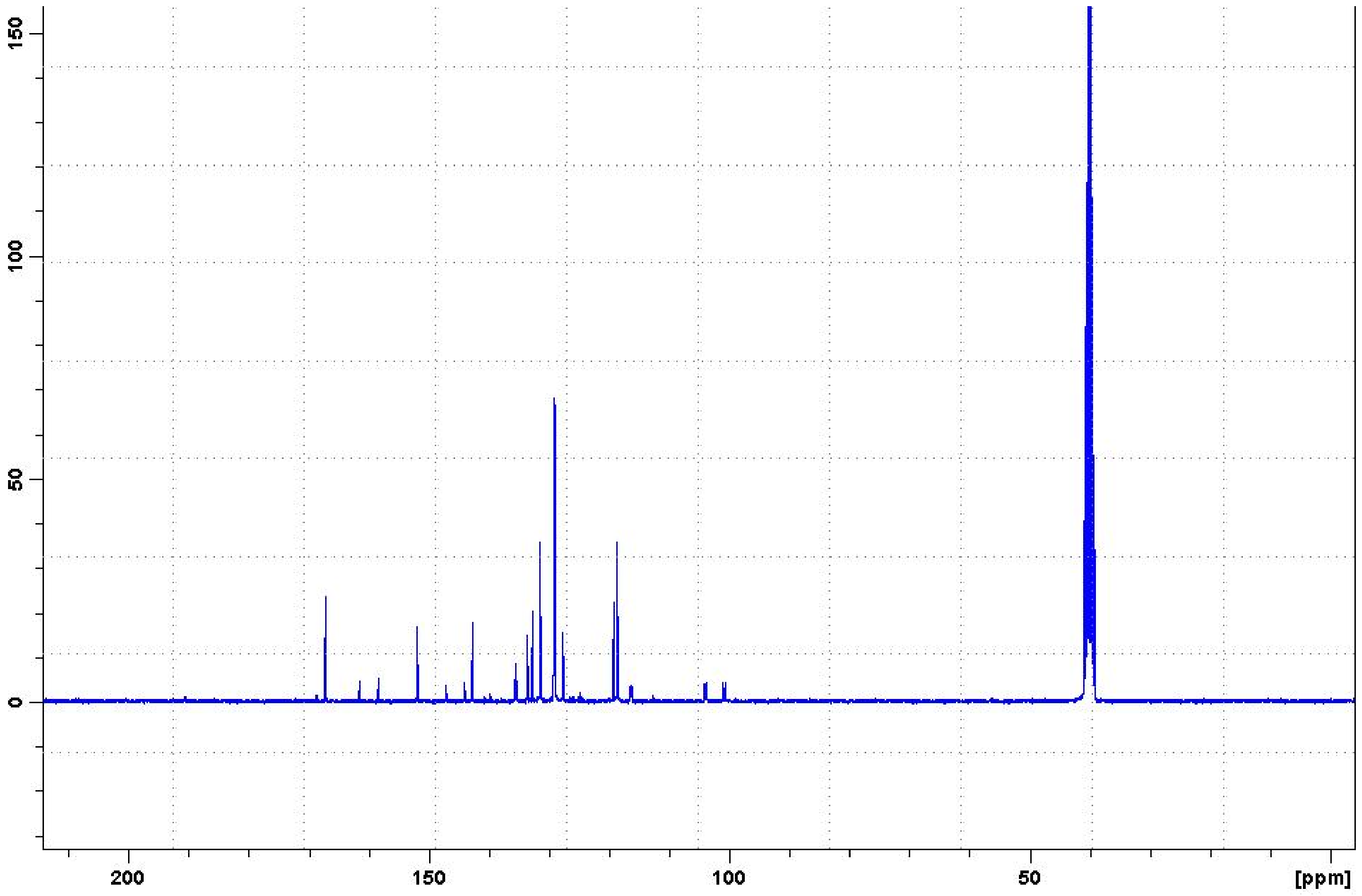

[ppm]

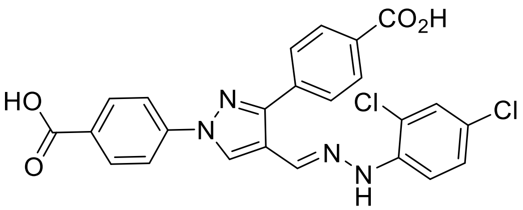

4,4'-(4-{(*E*)-[2-(2,4-dichlorophenyl)hydrazinylidene]methyl}-1*H*-pyrazole-1,3-diyl)dibenzoic acid (21)

Brownish solid (376 mg, 76%). ^{1}H NMR, 300 MHz (DMSO-d$_6$): δ 10.04 (s, 1H), 9.11 (s, 1H), 8.47 (s, 1H), 8.16-8.08 (m, 6H), 7.88 (d, J = 8.1 Hz, 2H), 7.54 (d, J = 8.8 Hz, 1H), 7.43 (d, J = 2.0 Hz, 1H), 7.25 (d, J = 8.8 Hz, 1H); ^{13}C NMR (75MHz, DMSO-d$_6$) δ = 167.5, 167.1, 150.7, 142.4, 141.0, 136.7, 133.7, 131.3, 131.1, 130.0, 129.3, 128.9, 128.7, 128.2, 127.6, 122.3, 119.5, 118.6, 116.7, 115.3.

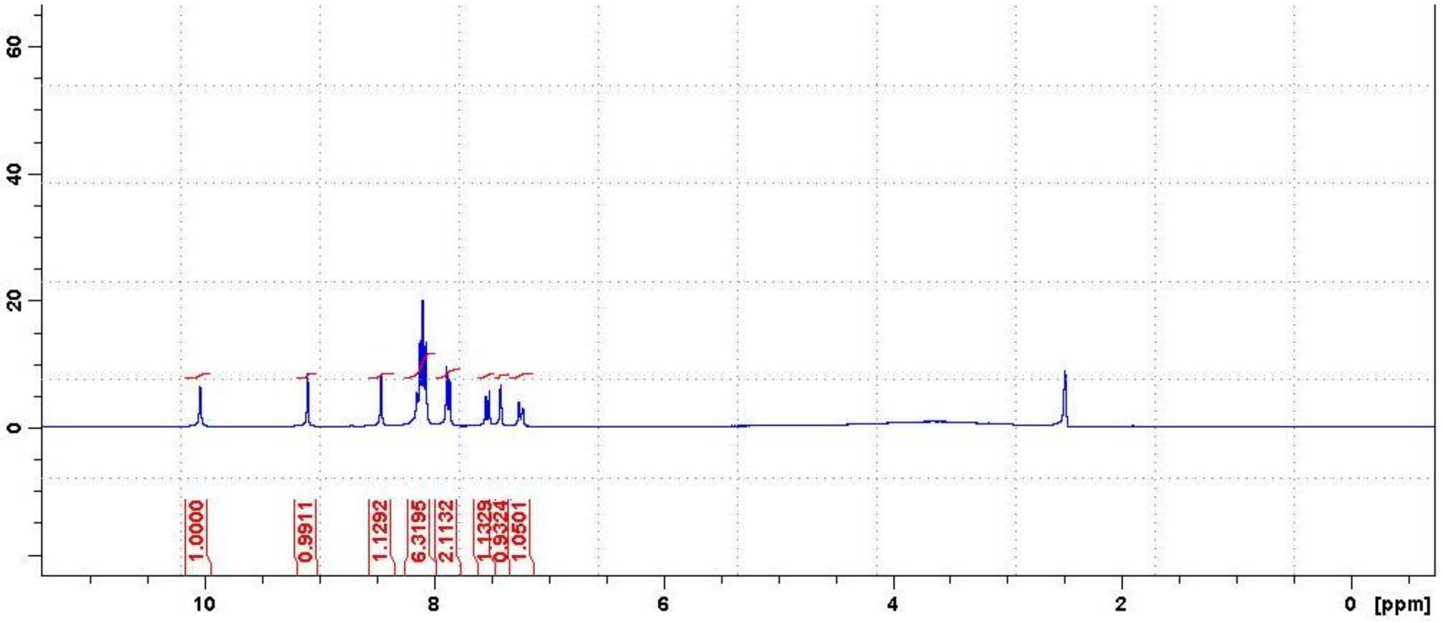

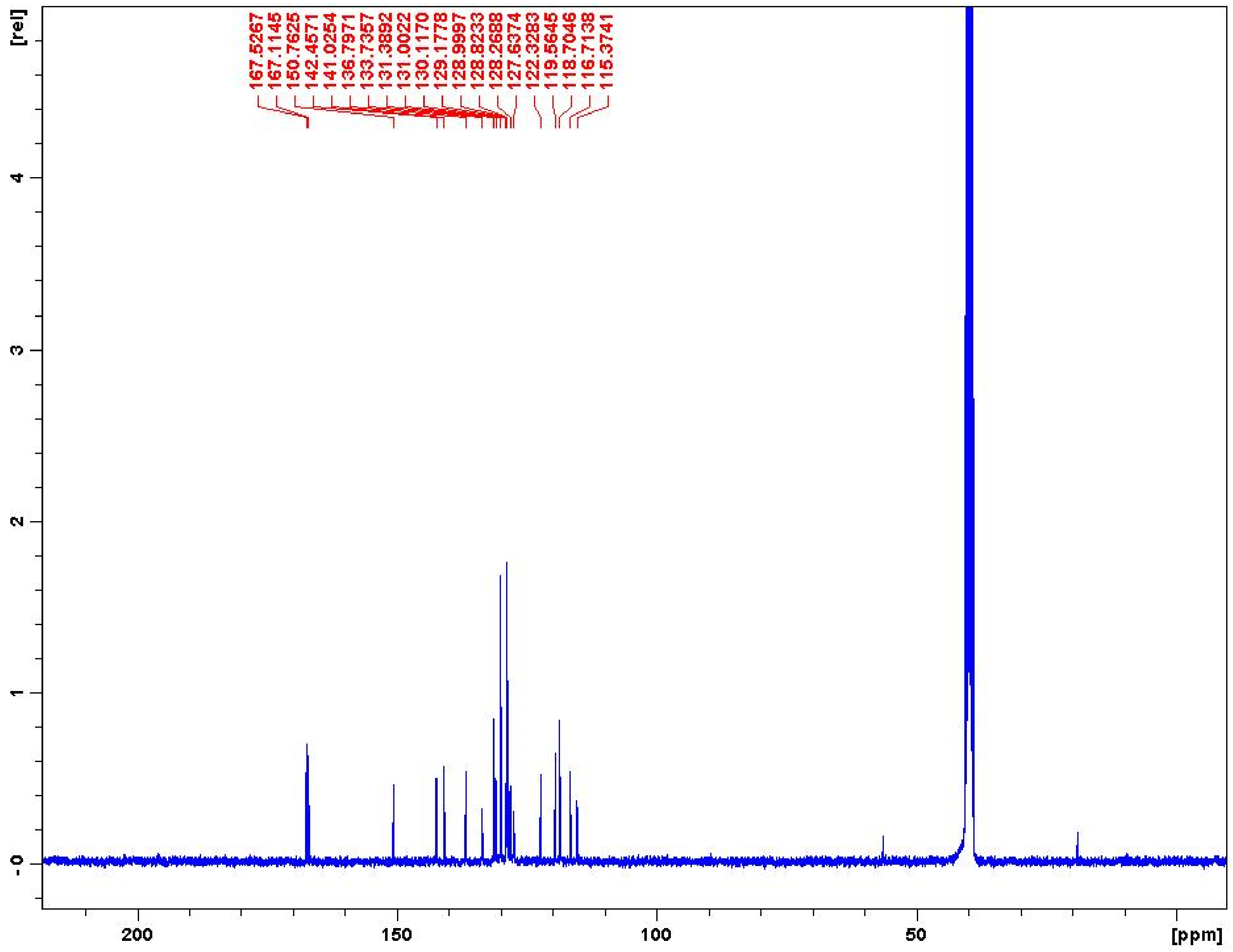

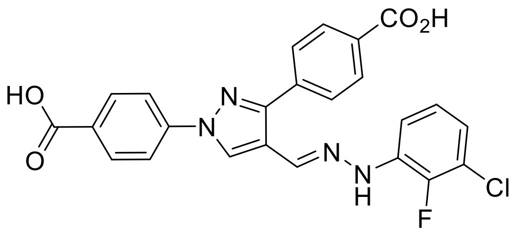

4-[1-(4-carboxyphenyl)-4-[(E)-[(3-chloro-2-fluoro-phenyl)hydrazono]methyl]pyrazol-3-yl]benzoic acid (22)

Brownish solid (358 mg, 75%). ^{1}H NMR, 300 MHz (DMSO-d_6): δ 10.40 (s, 1H), 9.09 (s, 1H), 8.32 (s, 1H), 8.11-8.07 (m, 6H), 7.88 (s, J = 7.8 Hz, 2H), 7.43 (t, J = 7.6 Hz, 1H), 7.05 (t, J = 7.7 Hz, 1H), 6.85 (t, J = 6.4 Hz, 1H); ^{13}C NMR (75MHz, DMSO-d_6) δ = 167.5, 167.1, 150.6, 144.7 (d, ^1J = 235.8 Hz), 142.4, 136.8, 135.3 (d, ^3J = 9.2), 133.2, 131.3, 131.1, 130.0, 129.3, 128.8, 127.7, 125.8 (d, J = 3.5 Hz), 119.9 (d, ^2J = 14.2 Hz), 119.4, 118.8, 118.6, 113.0.

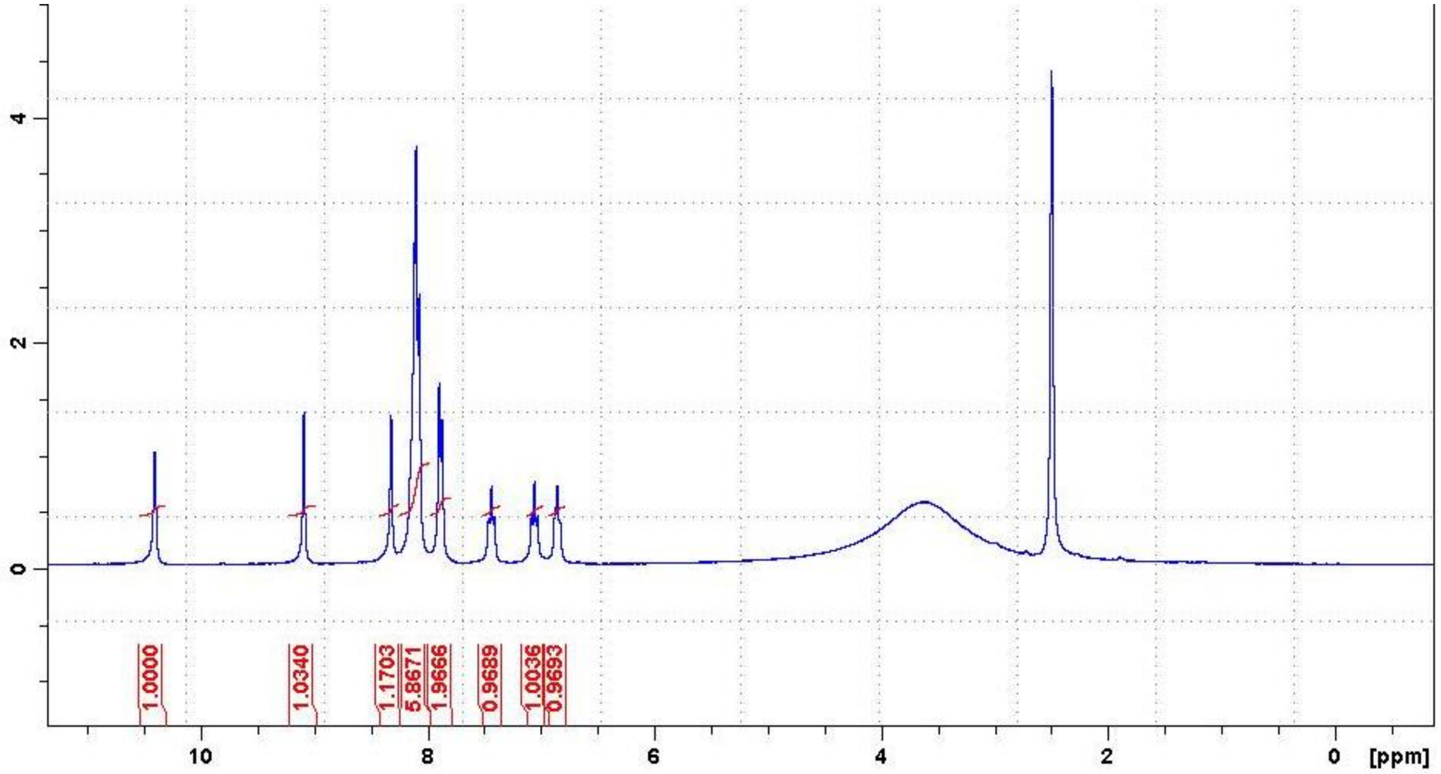
1.0000
1.0340
1.1703
5.8671
1.9666
0.9689
1.0036
0.9693
10
8
6
4
2
0
[ppm]

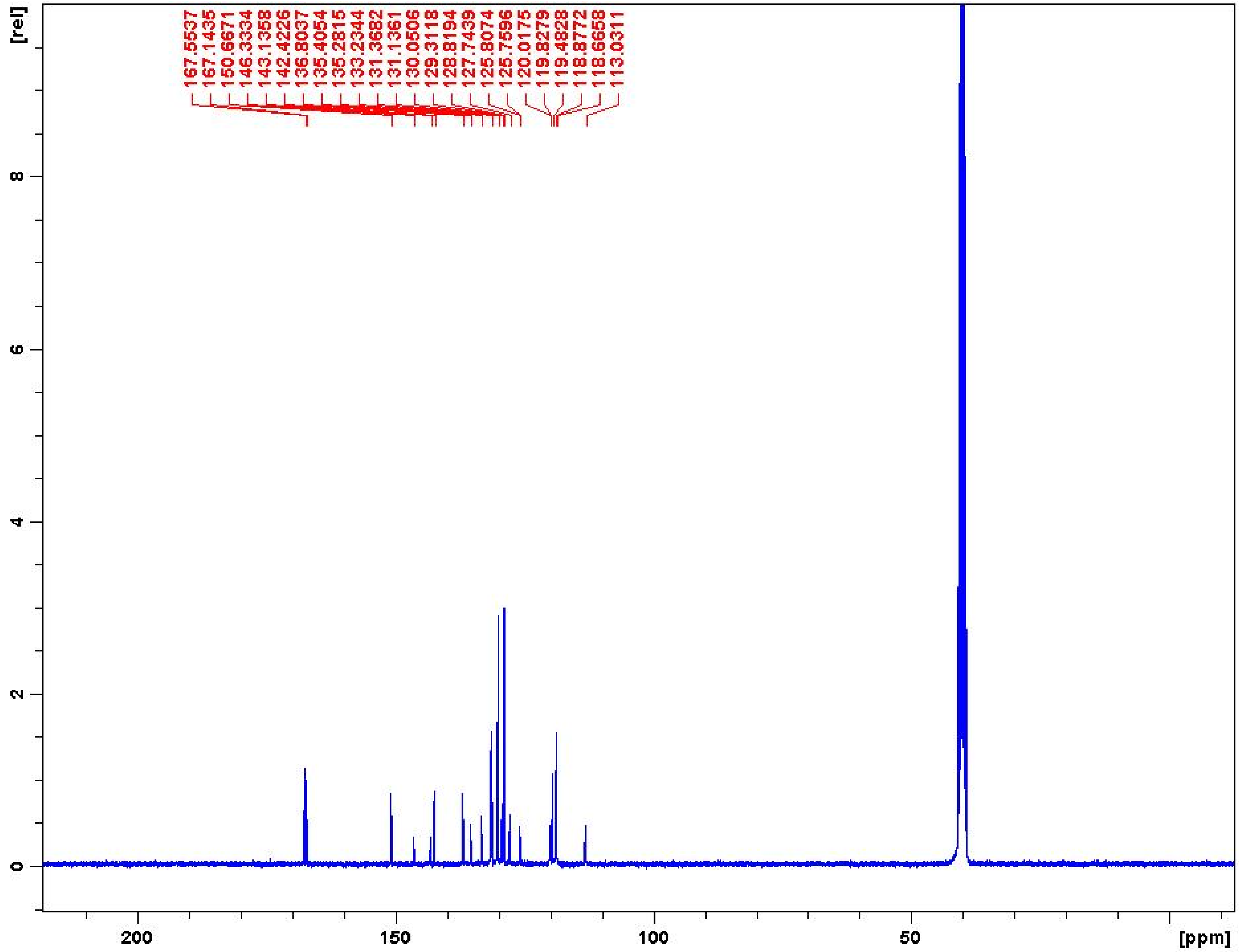
167.5537
167.1435
150.6671
146.3334
143.1358
142.4226
136.8037
135.4054
135.2815
133.2344
131.3682
131.1361
130.0506
129.3118
128.8194
127.7439
125.8074
125.7596
120.0175
119.8279
119.4828
118.8772
118.6658
113.0311
[rel]
200
150
100
50
[ppm]

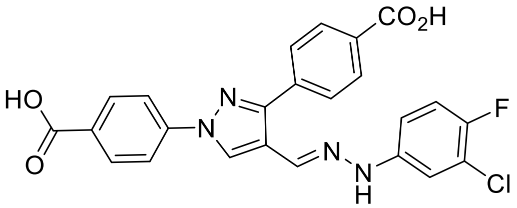

4,4'-(4-{(*E*)-[2-(3-chloro-4-fluorophenyl)hydrazinylidene]methyl}-1*H*-pyrazole-1,3-diyl)dibenzoic acid (23)

Brownish solid (368 mg, 77%). ^{1}H NMR, 300 MHz (DMSO-d$_6$): δ 10.83 (br s, 1H), 9.05 (s, 1H), 8.10-8.98 (m, 7H), 7.81 (d, J = 8.1 Hz, 2H), 7.25-7.19 (m, 2H), 6.98-6.94 (m, 1H); ^{13}C NMR (75MHz, DMSO-d$_6$) δ = 168.8, 168.4, 150.7, 151.1 ($^{1}J_{C-F}$ = 234.6 Hz), 150.7, 143.4, 141.2, 135.8, 135.1, 134.0, 131.0, 129.9, 128.2, 127.1, 120.3 ($^{2}J_{C-F}$ = 18.2 Hz), 119.2, 118.1, 117.6 ($^{2}J_{C-F}$ = 22.1 Hz), 112.6, 111.9.

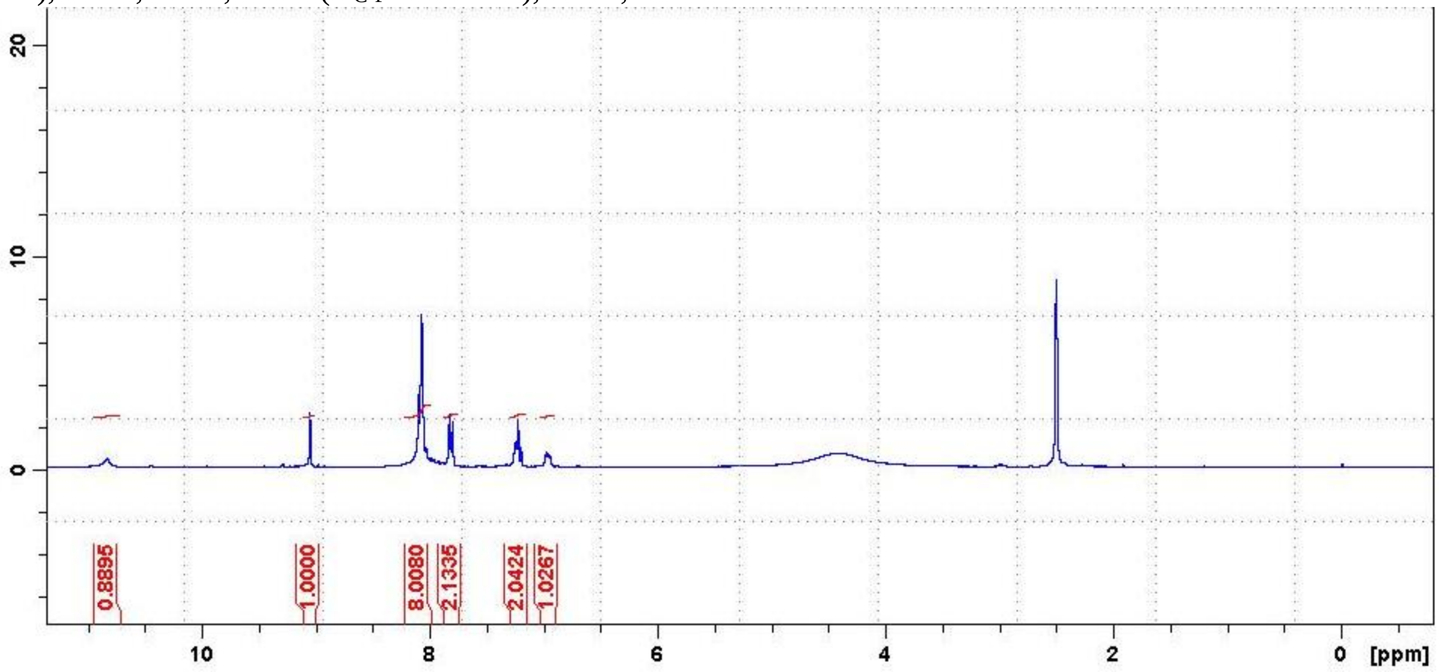

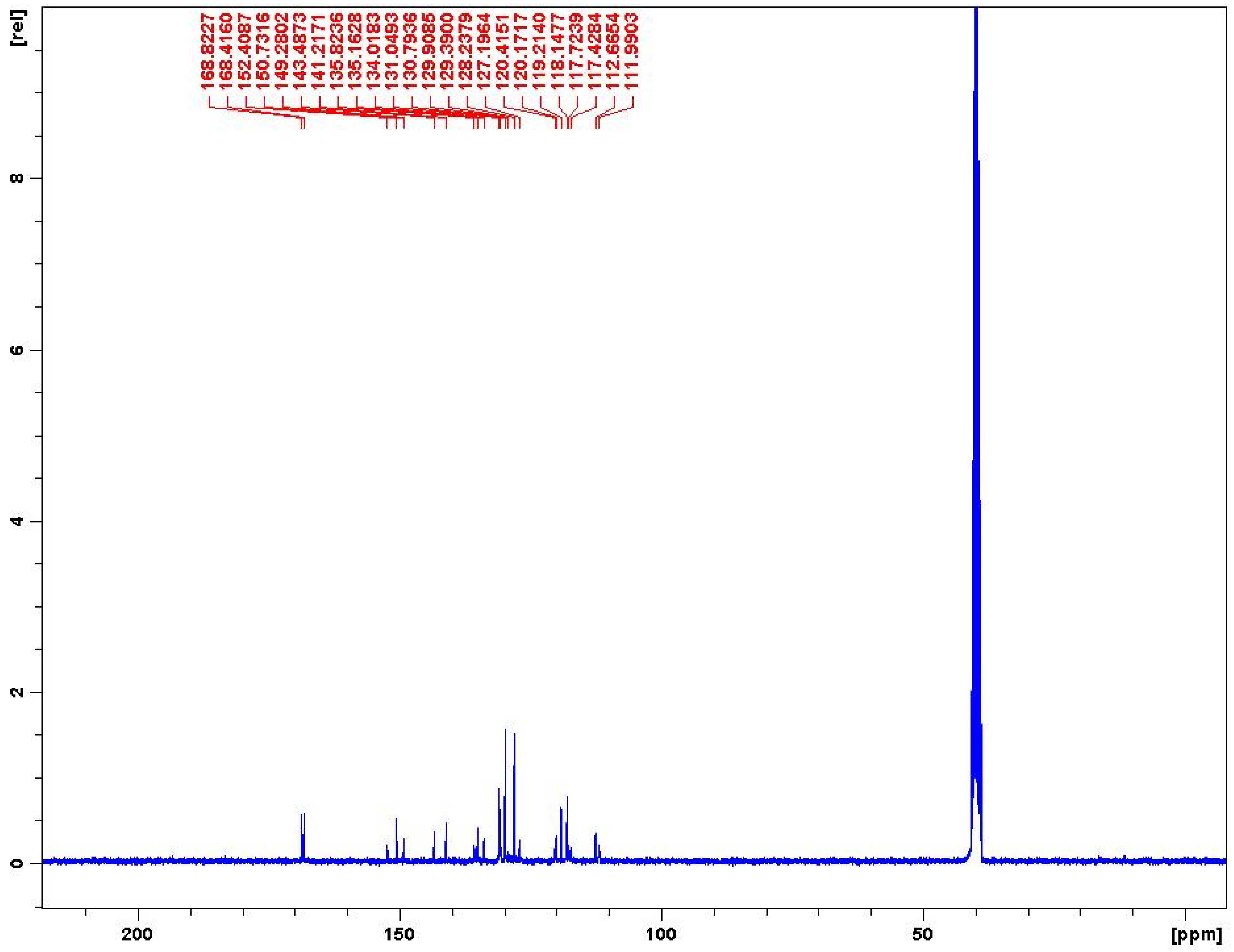

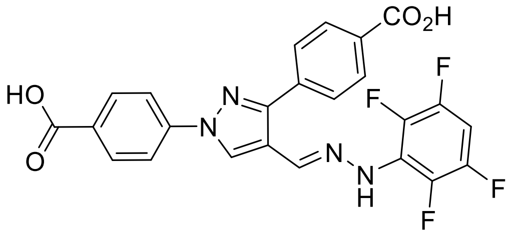

4,4'-(4-{(*E*)-[2-(2,3,5,6-tetrafluorophenyl)hydrazinylidene]methyl}-1*H*-pyrazole-1,3-diyl)dibenzoic acid (24)

Brownish solid (398 mg, 80%). ^{1}H NMR, 300 MHz (DMSO-d_6): δ 10.38 (s, 1H), 8.81 (s, 1H), 8.36 (s, 1H), 8.05-7.98 (m, 6H), 7.81 (d, J = 6.9 Hz, 2H), 7.25-7.13 (m, 1H); ^{13}C NMR (75MHz, DMSO-d_6) δ = 168.4, 168.1, 157.5, 151.0, 140.9, 138.5, 136.1, 135.8, 134.7, 131.0, 129.8, 128.2, 127.2, 118.3, 95.7 ($^{2}J_{C\text{-}F}$ = 25.0 Hz).

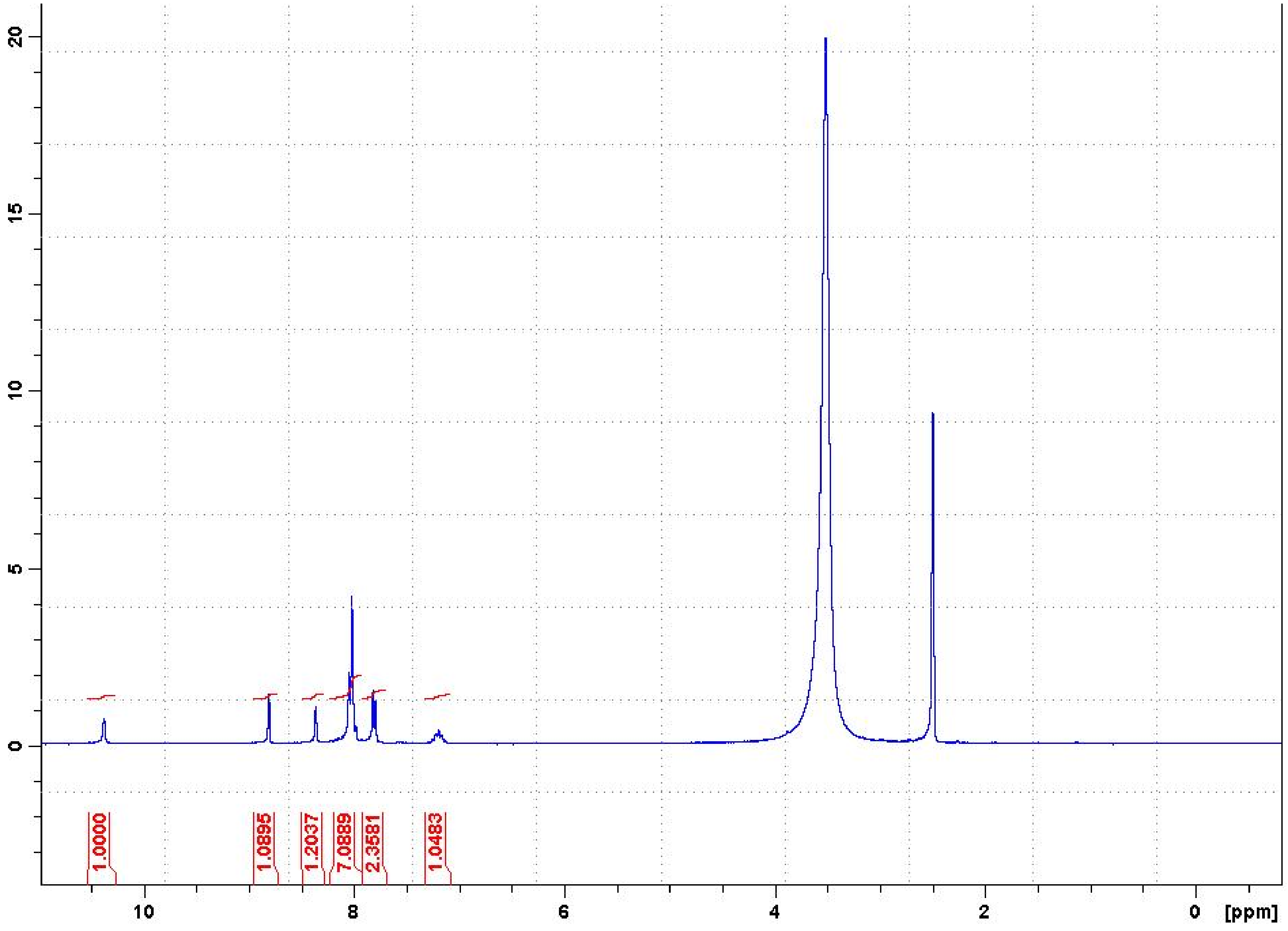

20
15
10
5
0
1.0000
1.0895
1.2037
7.0889
2.3581
1.0483
10
8
6
4
2
0
[ppm]

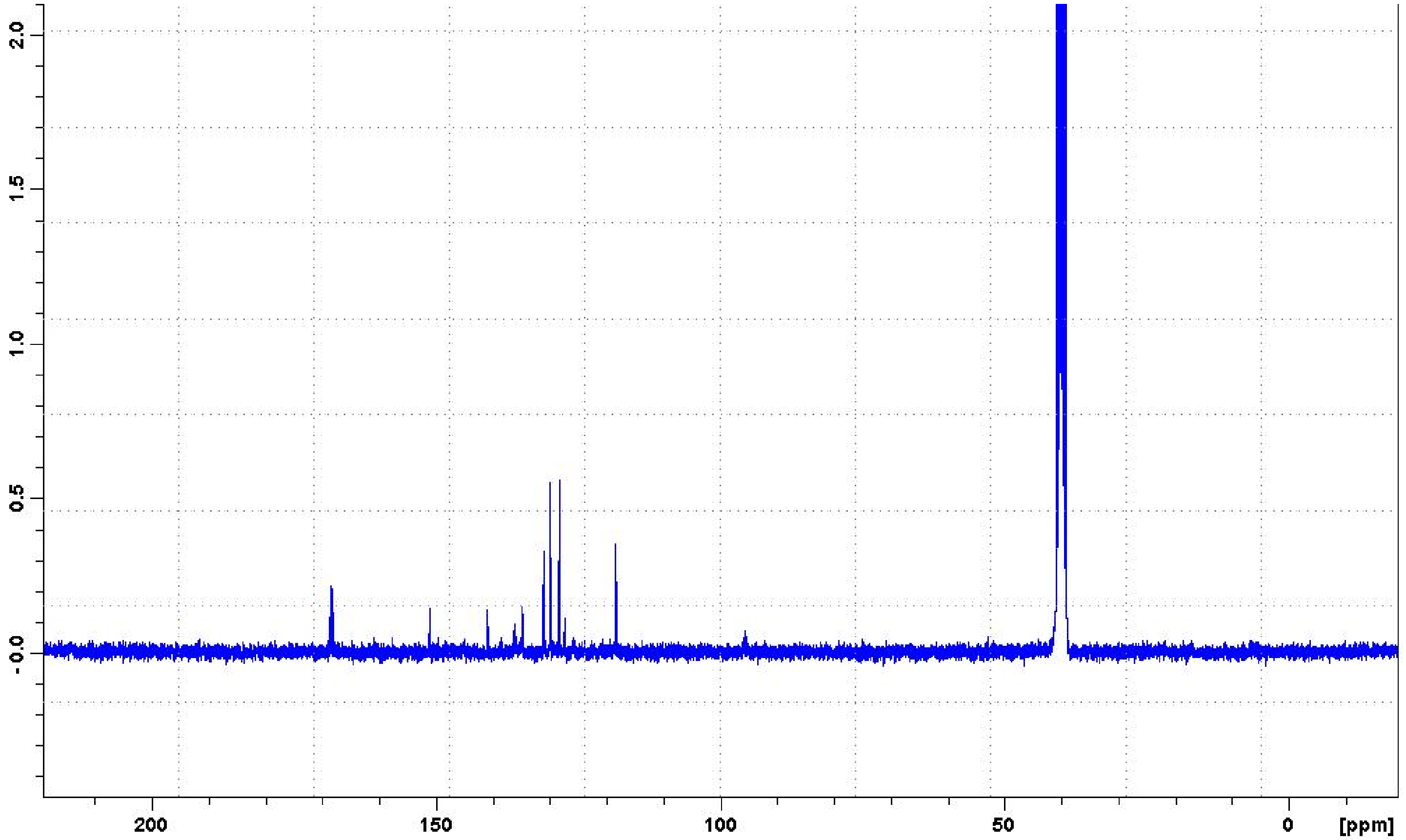

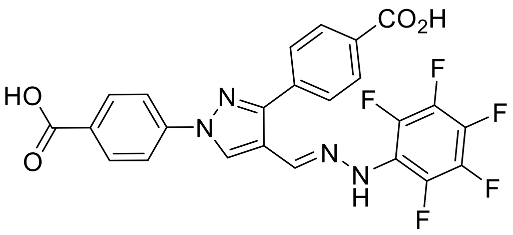

4,4'-(4-{(*E*)-[2-(2,3,4,5,6-pentafluorophenyl)hydrazinylidene]methyl}-1*H*-pyrazole-1,3-diyl)dibenzoic acid (25)

Brownish solid (423 mg, 82%). ^{1}H NMR, 300 MHz (DMSO-d$_6$): δ 10.13 (br s, 1H), 8.72 (s, 1H), 8.21 (s, 1H), 8.01 (s, 7H), 7.84-7.81 (m, 2H); ^{13}C NMR (75 MHz, DMSO-d$_6$) δ = 167.6, 167.2, 150.5, 142.1, 139.9-139.7 (m), 138.9, 136.4, 135.5, 135.3, 131.2, 129.8, 129.6-129.2 (m), 129.4, 128.6, 127.4, 121.6, 118.7, 118.5.

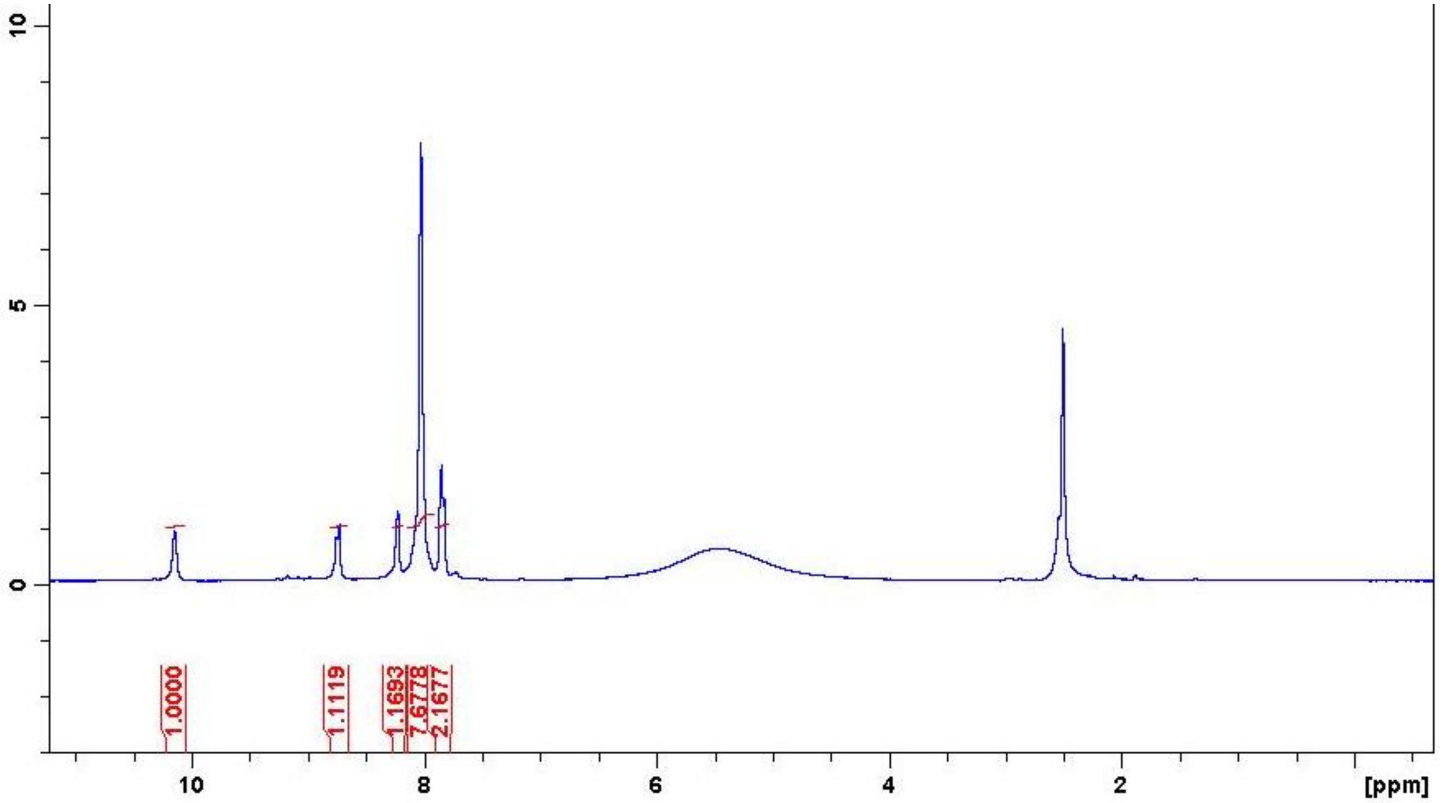

10
5
0
1.0000
1.1119
1.1693
7.6778
2.1677
10
8
6
4
2
[ppm]

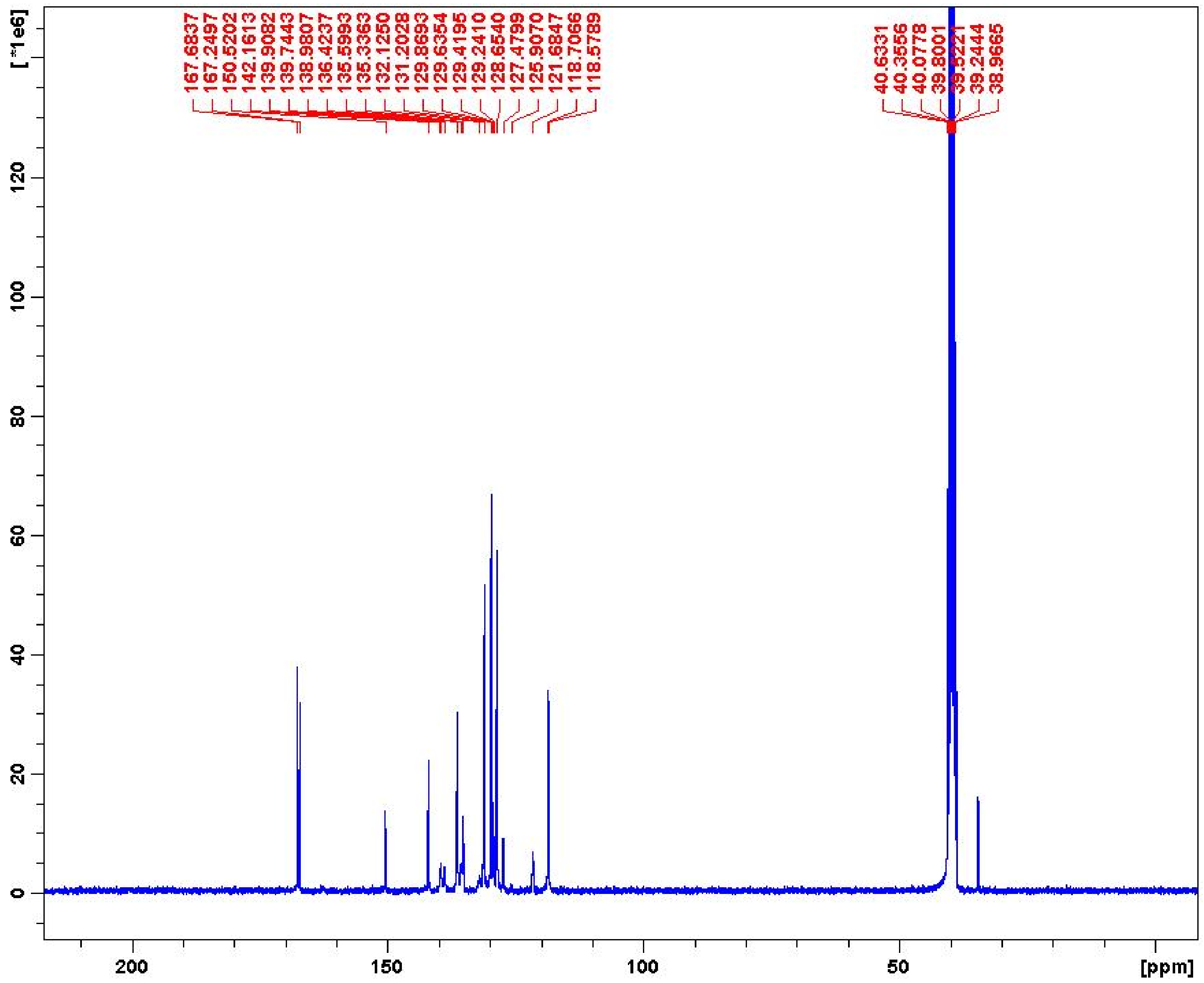

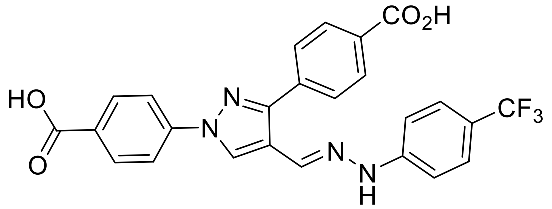

4,4'-(4-{(*E*)-[2-(4-trifluoromethylphenyl)hydrazinylidene]methyl}-1*H*-pyrazole-1,3-diyl)dibenzoic acid (26)

Brownish solid (370 mg, 75%). ^1H NMR, 300 MHz (DMSO-d_6): δ 10.78 (s, 1H), 9.07 (s, 1H), 8.14-8.07 (m, 8H), 7.90 (d, J = 8.3 Hz, 2H), 7.48 (d, J = 8.6 Hz, 2H), 7.13 (d, J = 8.5 Hz, 2H); ^{13}C NMR (75 MHz, DMSO-d_6) δ = 167.5, 167.1, 150.6, 148.6, 142.4, 136.8, 131.5, 131.3, 131.0, 130.0, 129.1, 128.8, 127.8, 126.7, 123.6, 119.4, 118.6, 111.8.

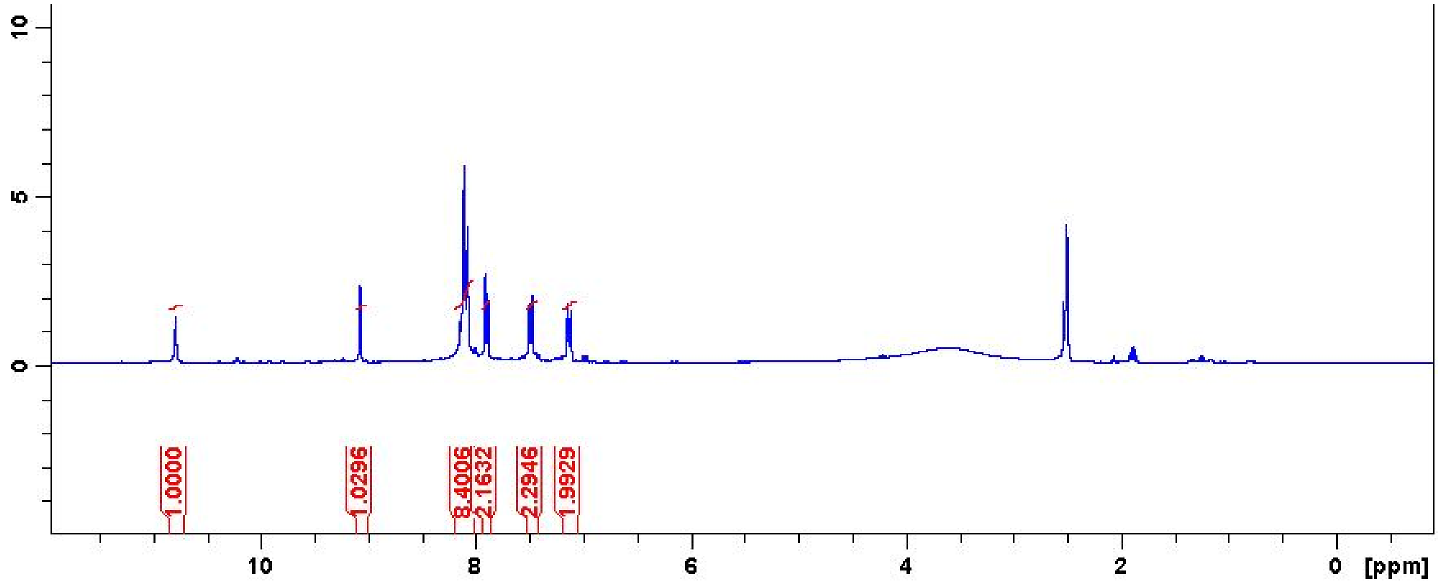
1.0000
1.0296
8.4006
2.1632
2.2946
1.9929
[ppm]

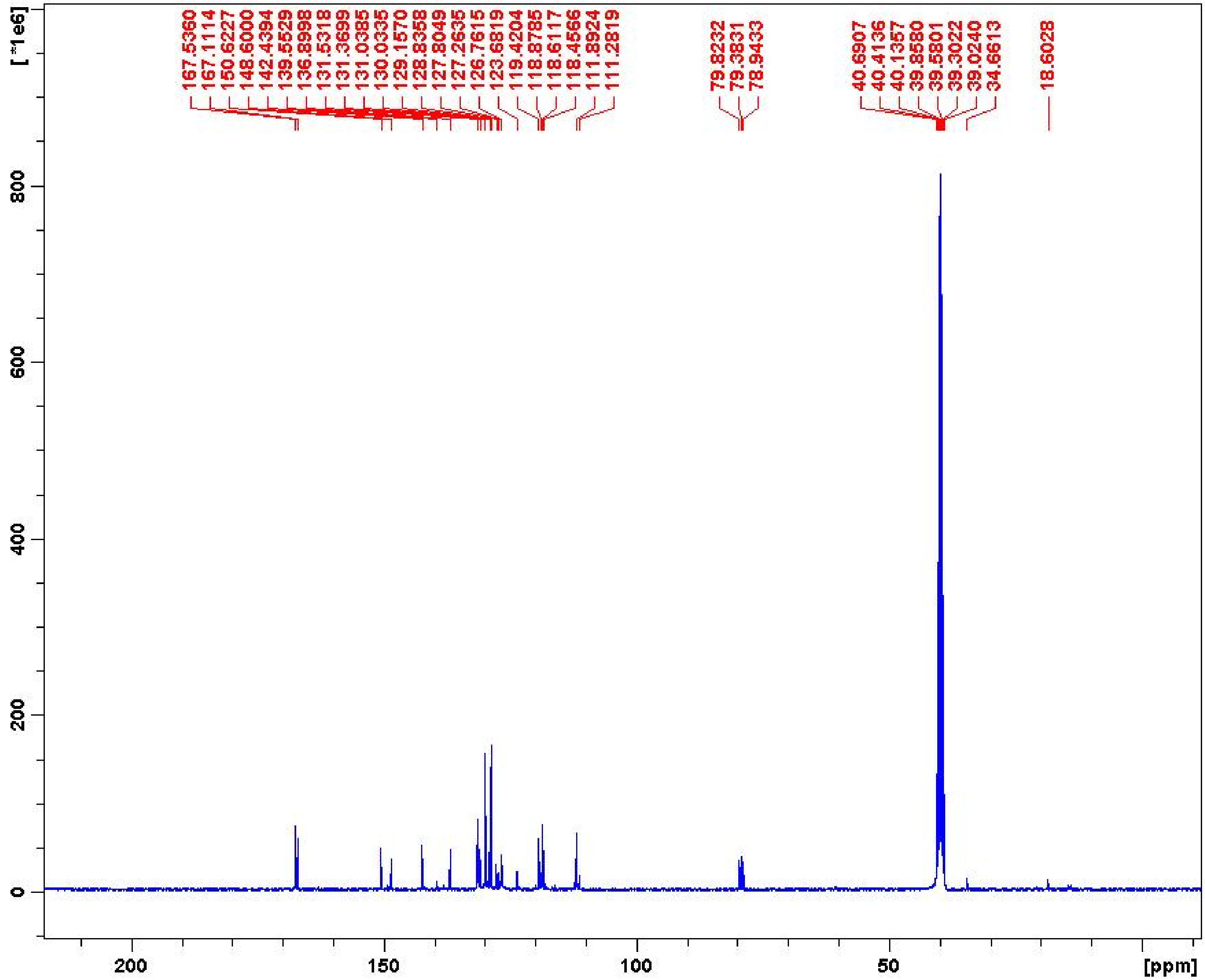
[*1e6]
167.5360
167.1114
150.6227
148.6000
142.4394
139.5529
136.8998
131.5318
131.3699
131.0385
130.0335
129.1570
128.8358
127.8049
127.2635
126.7615
123.6819
119.4204
118.8785
118.6117
118.4566
111.8924
111.2819
79.8232
79.3831
78.9433
40.6907
40.4136
40.1357
39.8580
39.5801
39.3022
39.0240
34.6613
18.6028
[ppm]

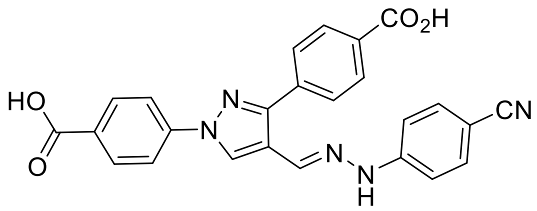

4-[1-(4-carboxyphenyl)-4-[(E)-[(4-cyanophenyl)hydrazono]methyl]pyrazol-3-yl]benzoic acid (27)

Brownish solid (342 mg, 76%). ^{1}H NMR, 300 MHz (DMSO-d_6): δ 10.92 (s, 1H), 9.12 (s, 1H), 8.16-8.07 (m, 8H), 7.90 (d, J = 8.3 Hz, 2H), 7.59 (d, J = 8.7 Hz, 2H), 7.10 (d, J = 8.6 Hz, 2H); ^{13}C NMR (75 MHz, DMSO-d_6) δ = 167.4, 167.0, 150.7, 148.9, 142.4, 136.8, 134.0, 132.6, 131.4, 131.1, 130.0, 129.2, 128.8, 128.0, 120.5, 119.2, 118.7, 112.4, 99.5.

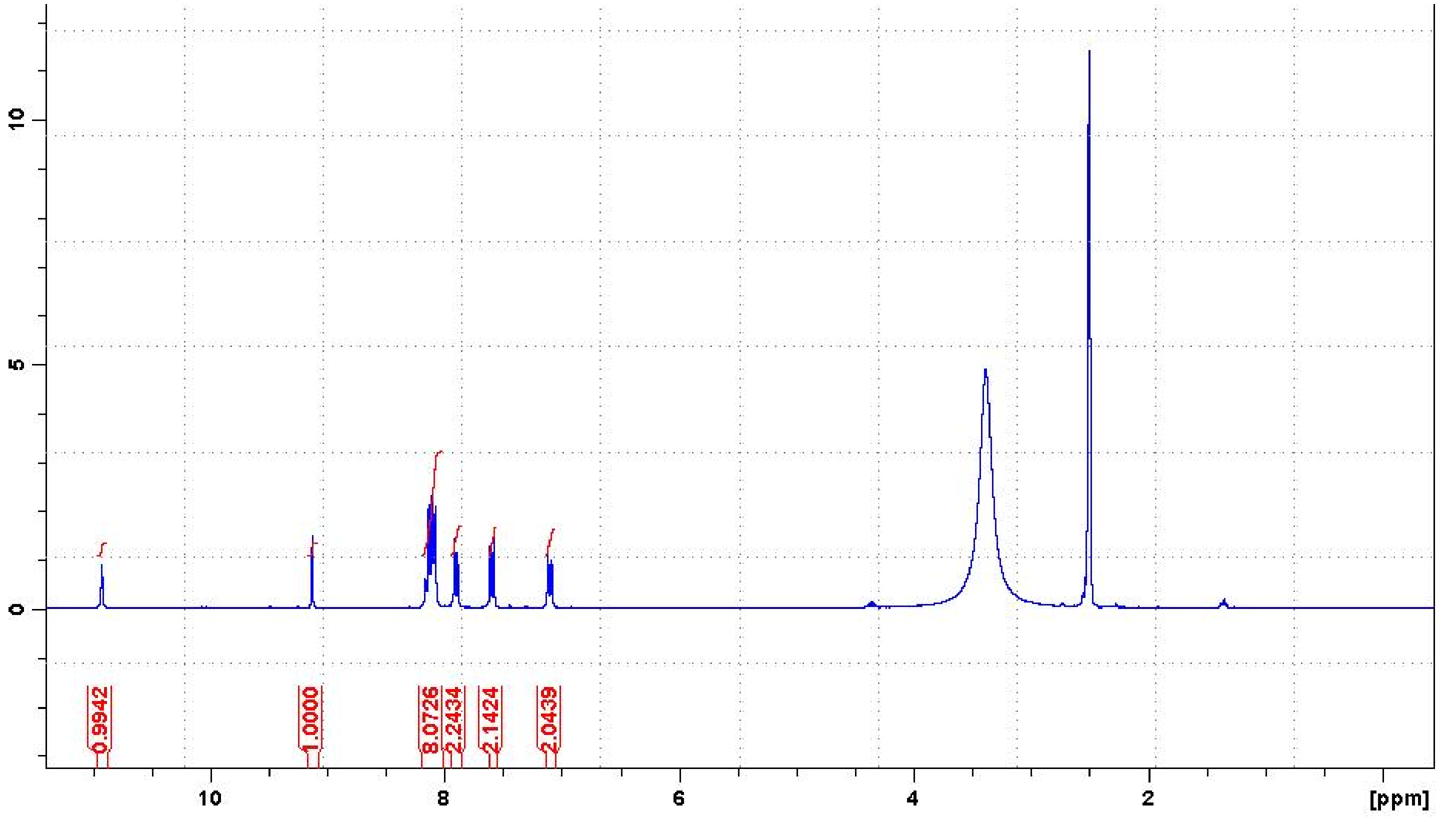

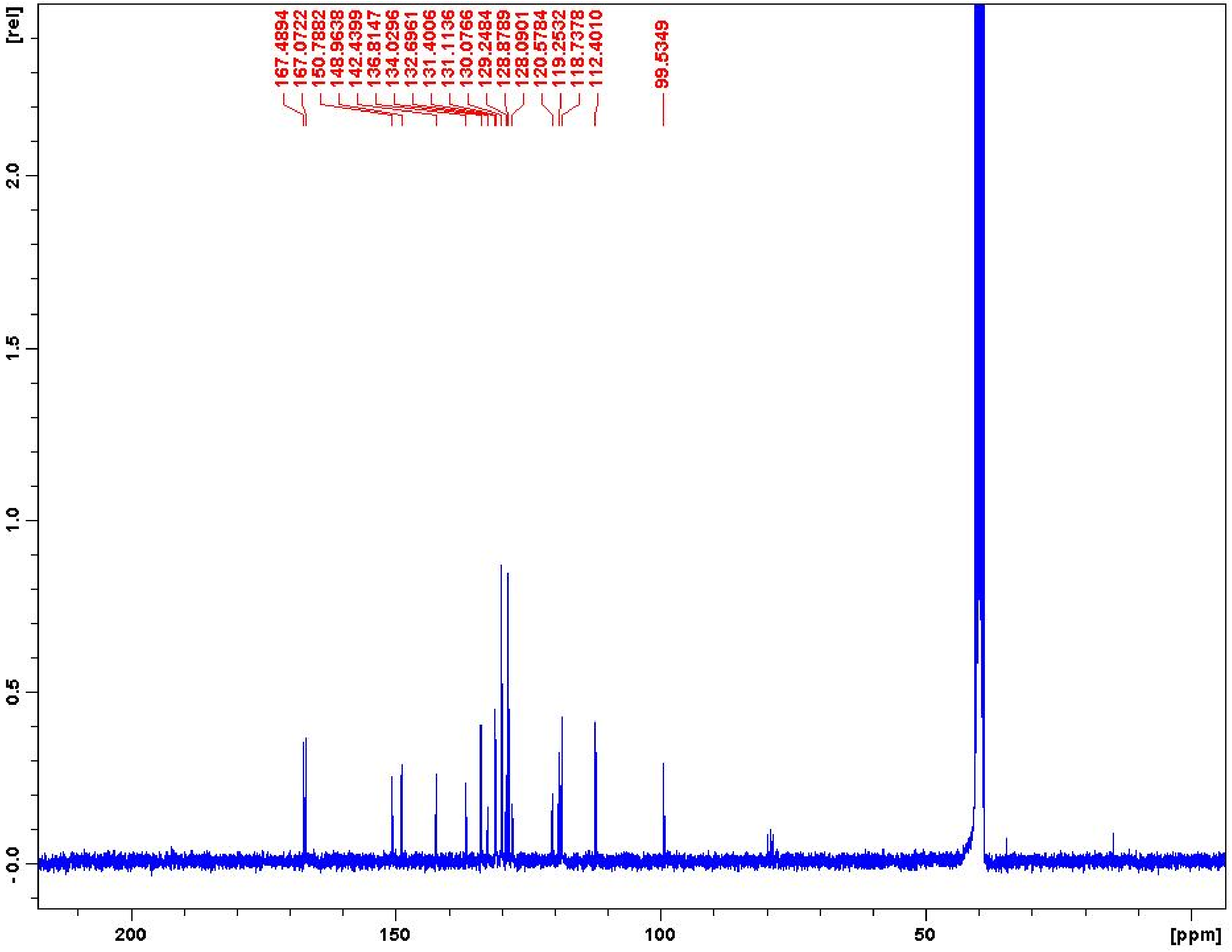

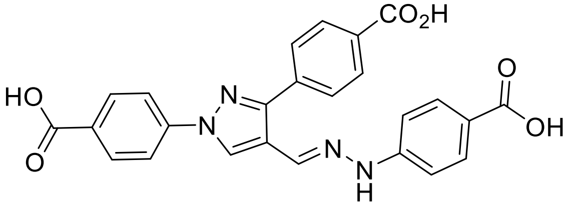

4,4'-(4-{(*E*)-[2-(4-corboxyphenyl)hydrazinylidene]methyl}-1*H*-pyrazole-1,3-diyl)dibenzoic acid (28)

Brownish solid (347 mg, 74%). ^{1}H NMR, 300 MHz (DMSO-d_6): δ 12.8 (br s, 3H), 10.77 (s, 1H), 9.12 (s, 1H), 8.17-8.07 (m, 8H), 7.91 (s, 1H), 7.79-7.77 (d, J = 8.58 Hz, 2H), 7.05 (d, J = 8.4 Hz, 2H); ^{13}C NMR (75 MHz, DMSO-d_6) δ = 167.7, 167.5, 167.1, 150.6, 149.1, 142.4, 136.9, 131.5, 131.4, 131.0, 130.0, 129.9, 129.1, 129.0, 128.9, 120.5, 119.4, 118.6, 111.4.

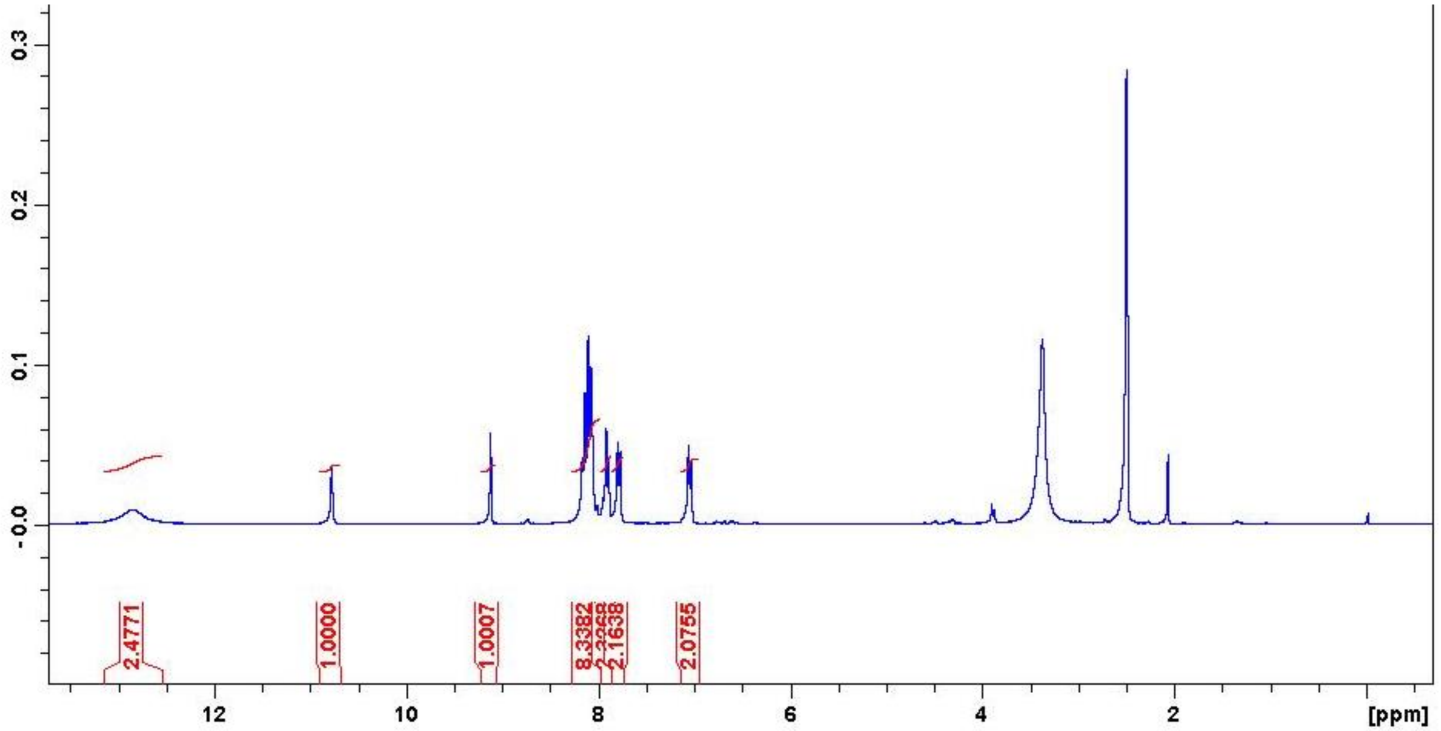

0.3
0.2
0.1
-0.0
2.4771
1.0000
1.0007
8.3382
2.3268
2.1638
2.0755
12
10
8
6
4
2
[ppm]

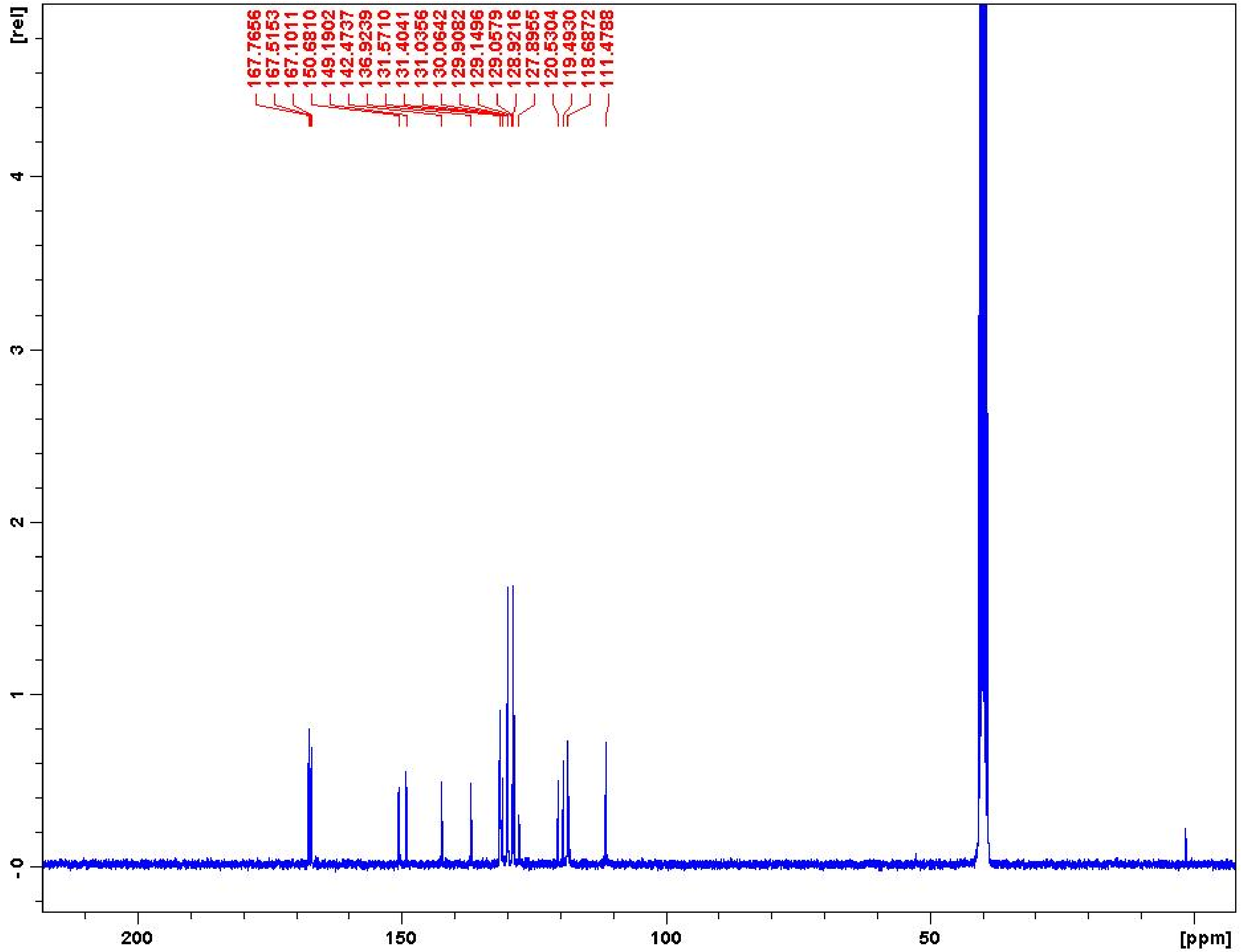

[rel]
167.7656
167.5153
167.1011
150.6810
149.1902
142.4737
136.9239
131.5710
131.4041
131.0356
130.0642
129.9082
129.1496
129.0579
128.9216
127.8955
120.5304
119.4930
118.6872
111.4788
4
3
2
1
-0
200
150
100
50
[ppm]

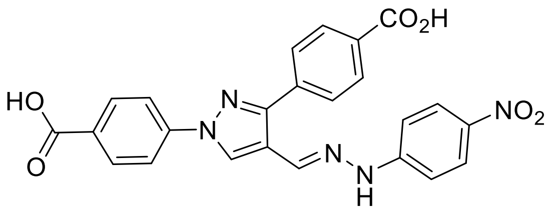

4,4'-(4-{(*E*)-[2-(4-nitrophenyl)hydrazinylidene]methyl}-1*H*-pyrazole-1,3-diyl)dibenzoic acid (29)

Reddish solid (372 mg, 79%). ^{1}H NMR, 300 MHz (DMSO-d$_6$): δ 11.31 (s, 1H), 9.17 (s, 1H), 8.15-8.08 (m, 9H), 7.89 (d, J = 8.0 Hz, 2H), 7.10 (d, J = 8.4 Hz, 2H); ^{13}C NMR (75 MHz, DMSO-d$_6$) δ = 167.7, 167.2, 151.0, 150.9, 142.2, 138.5, 136.5, 134.6, 131.6, 131.3, 130.0, 129.8, 128.8, 128.3, 126.5, 118.9, 118.7, 111.5.

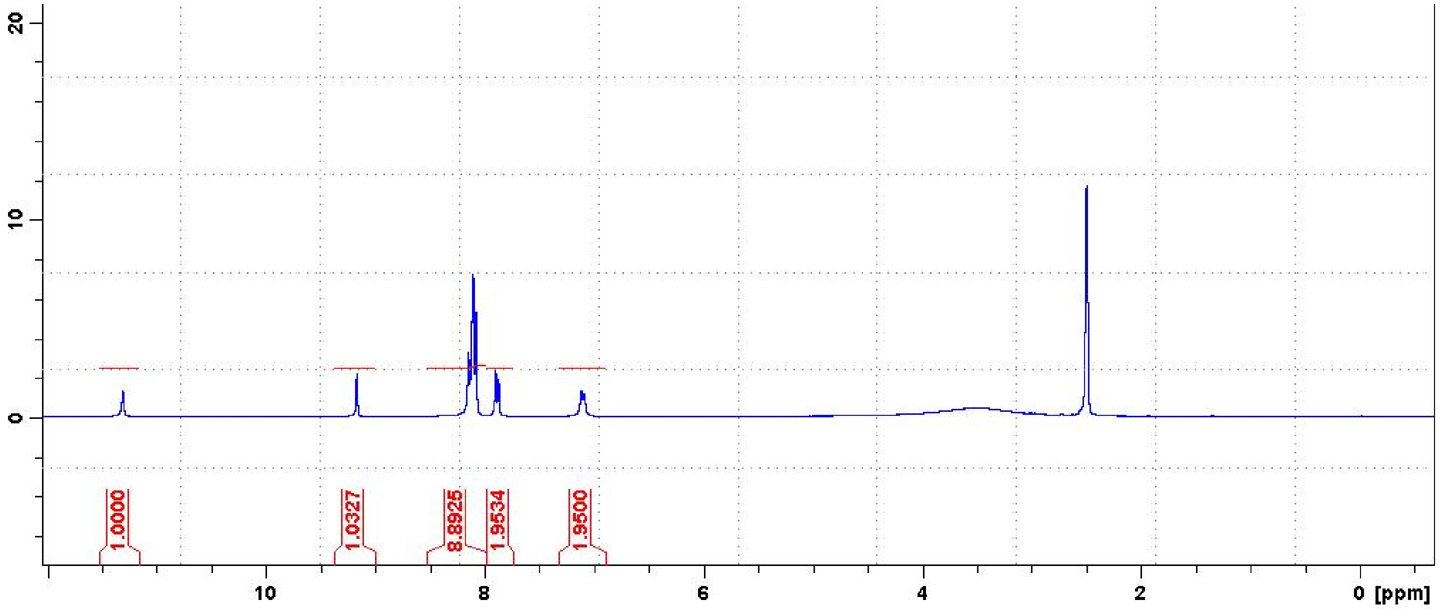

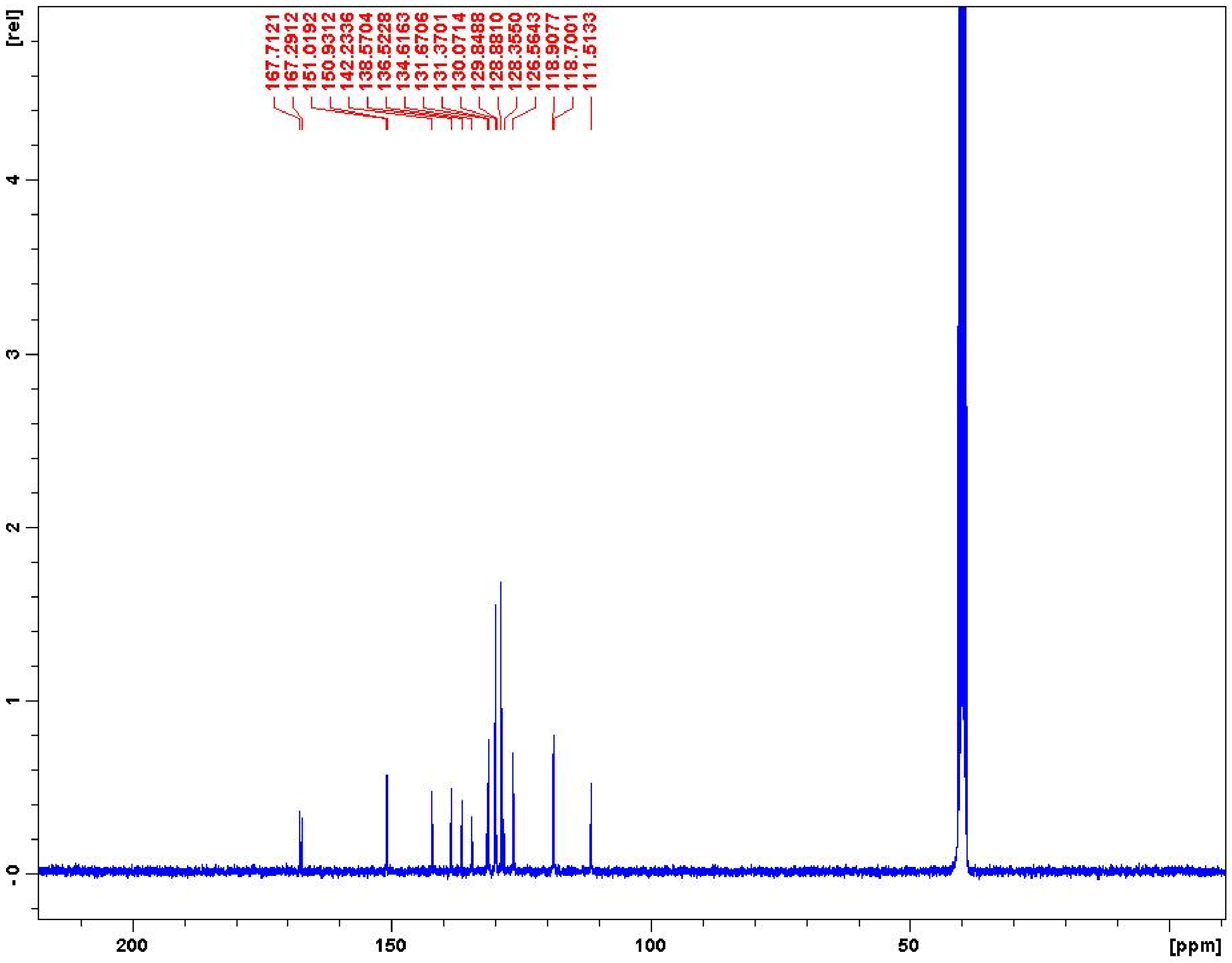
167.7121
167.2912
151.0192
150.9312
142.2336
138.5704
136.5228
134.6163
131.6706
131.3701
130.0714
129.8488
128.8810
128.3550
126.5643
118.9077
118.7001
111.5133
[rel]
-0
1
2
3
4
200
150
100
50
[ppm]
